# Supplementary material for: Detection of cell-type-specific risk-CpG sites in epigenome-wide association studies
Source: Nat Commun. 2019 Jul 15;10:3113. doi: 10.1038/s41467-019-10864-z (PMC6629651; doi:10.1038/s41467-019-10864-z)
Supplement: Supplementary file 1 — Supplementary Information [file 41467_2019_10864_MOESM1_ESM.pdf]

Supplementary Materials to:

Detection of Cell-Type-Specific Risk-CpG Sites

in Epigenome-Wide Association Studie

Xiangyu Luo[1,2], Can Yang[3,*], Yingying Wei[2,*]

[1]Institute of Statistics and Big Data, Renmin University of China, Beijing, China.

[2]Department of Statistics, The Chinese University of Hong Kong, Hong Kong SAR, China.

[3]Department of Mathematics, The Hong Kong University of Science and Technology, Hong Kong SAR, China.

[*]Correspondence should be addressed to Can Yang (email: macyang@ust.hk) or to Yingying Wei (email: yweicuhk@gmail.com)

Supplementary Figure 1.

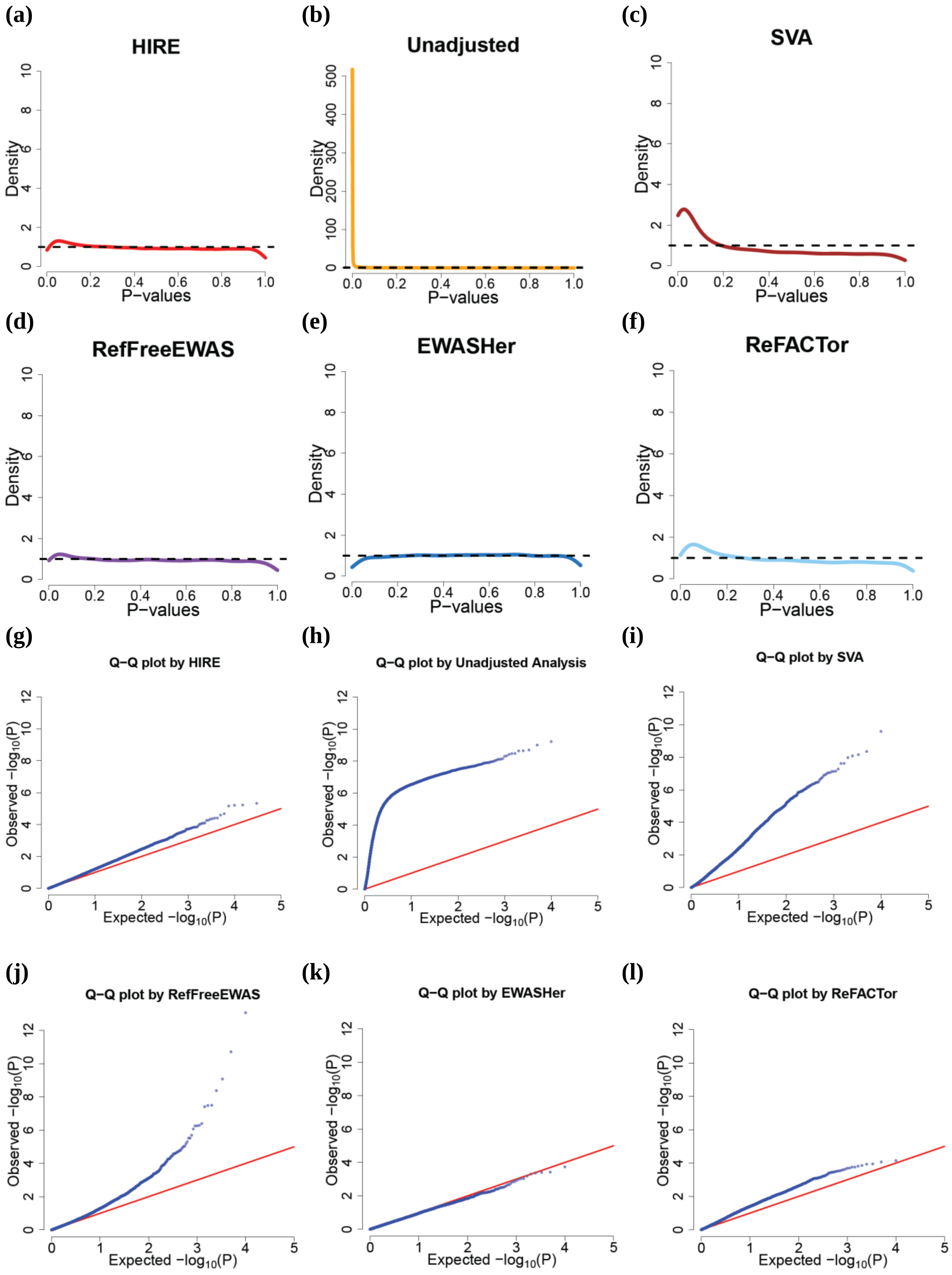

P-value density plots and Q-Q plots for HIRE and the commonly used methods in the “true null”setting with K = 3 and n = 180. P-value density plots for (a) HIRE, (b) unadjusted analysis, (c) SVA, (d) RefFreeEWAS, (e) EWASHer, and (f) ReFACTor. Q-Q plots for (g) HIRE, (h) unadjusted analysis, (i) SVA, (j) RefFreeEWAS, (k) EWASHer, and (l) ReFACTor.

Supplementary Figure 2.

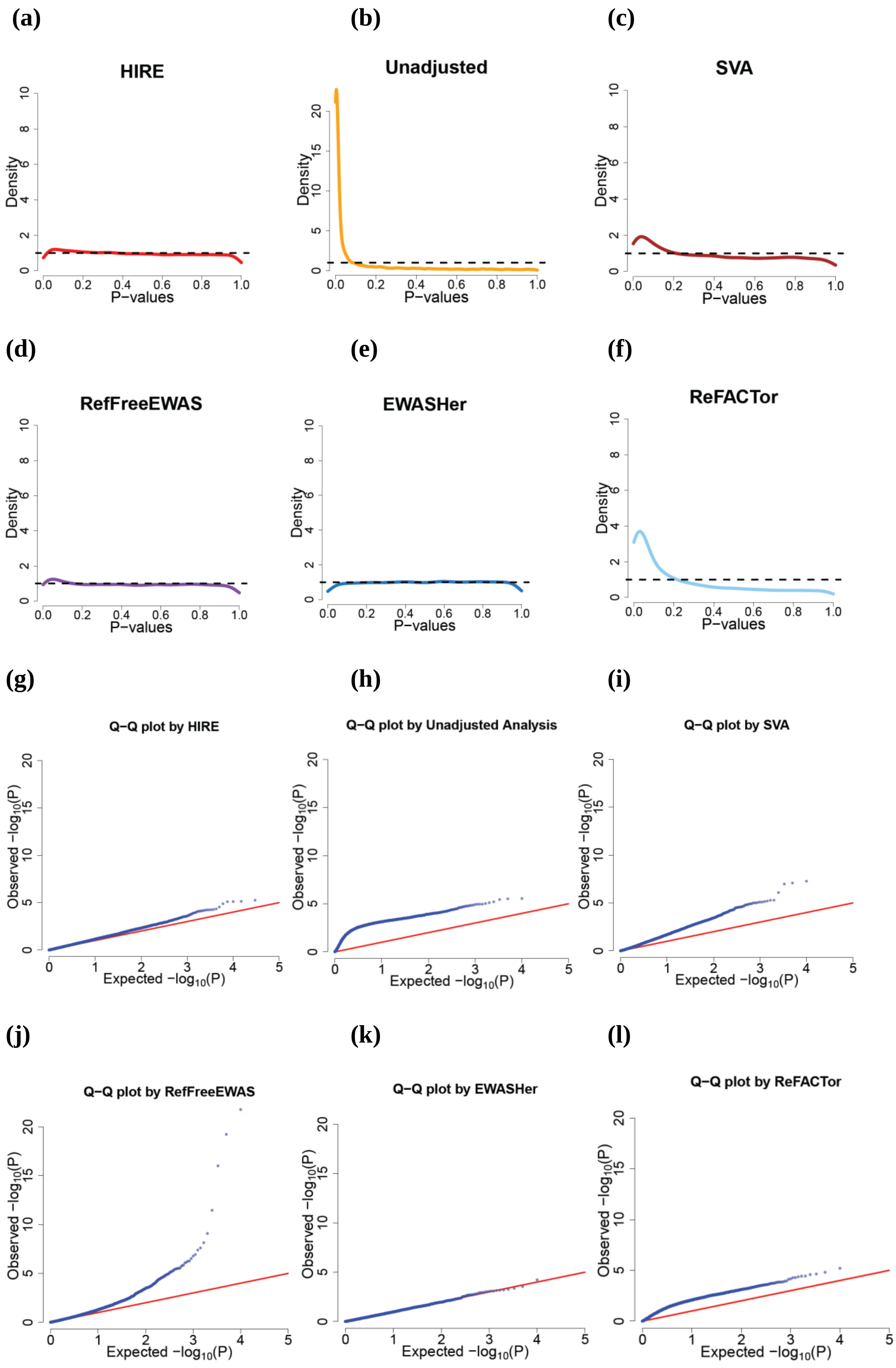

P-value density plots and Q-Q plots for HIRE and the commonly used methods in the “true null”setting with K = 3 and n = 300. P-value density plots for (a) HIRE, (b) unadjusted analysis, (c) SVA, (d) RefFreeEWAS, (e) EWASHer, and (f) ReFACTor. Q-Q plots for (g) HIRE, (h) unadjusted analysis, (i) SVA, (j) RefFreeEWAS, (k) EWASHer, and (l) ReFACTor.

Supplementary Figure 3.

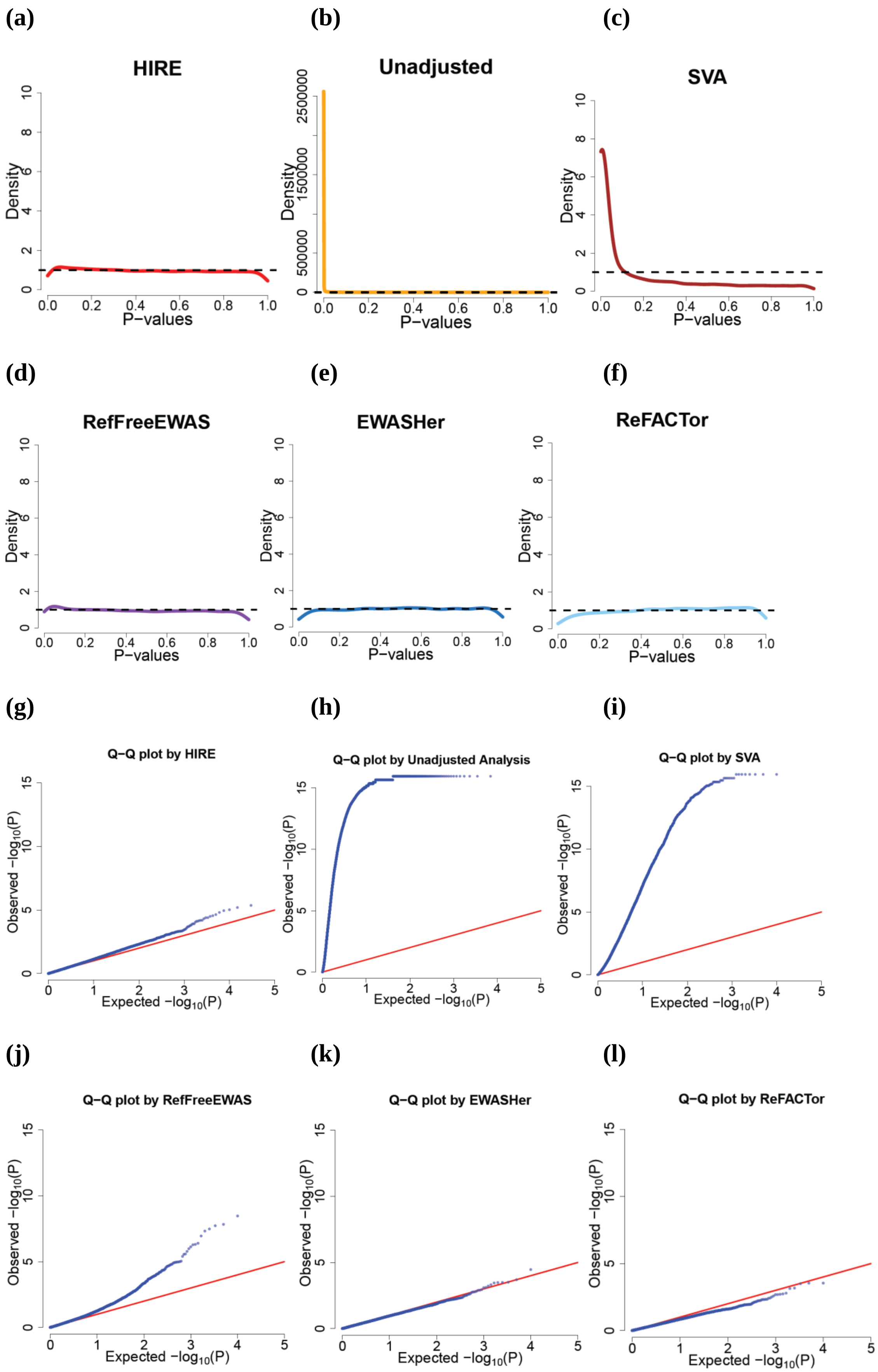

P-value density plots and Q-Q plots for HIRE and the commonly used methods in the “true null”setting with K = 3 and n = 600. P-value density plots for (a) HIRE, (b) unadjusted analysis, (c) SVA, (d) RefFreeEWAS, (e) EWASHer, and (f) ReFACTor. Q-Q plots for (g) HIRE, (h) unadjusted analysis, (i) SVA, (j) RefFreeEWAS, (k) EWASHer, and (l) ReFACTor.

Supplementary Figure 4.

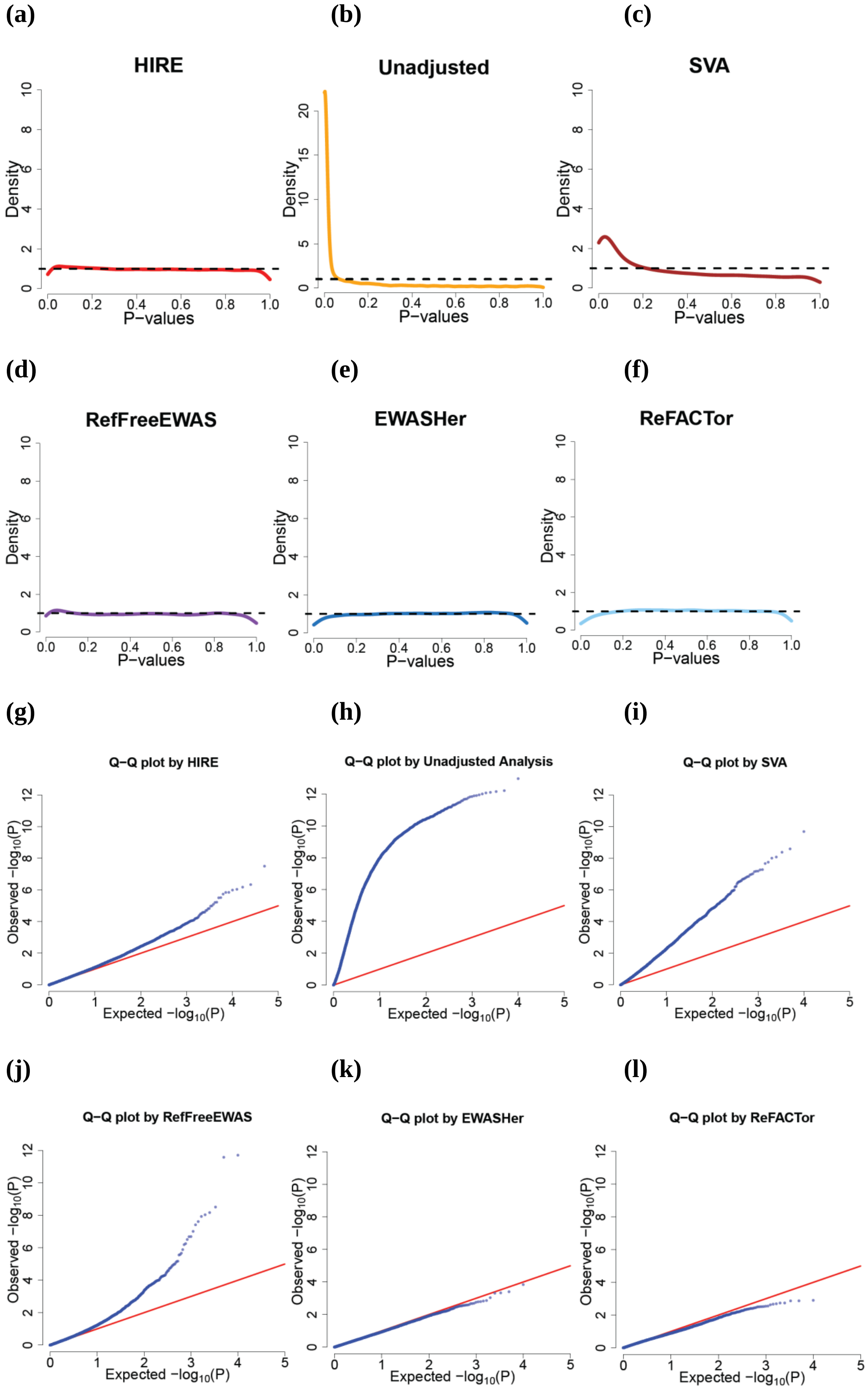

P-value density plots and Q-Q plots for HIRE and the commonly used methods in the “true null”setting with K = 5 and n = 180. P-value density plots for (a) HIRE, (b) unadjusted analysis, (c) SVA, (d) RefFreeEWAS, (e) EWASHer, and (f) ReFACTor. Q-Q plots for (g) HIRE, (h) unadjusted analysis, (i) SVA, (j) RefFreeEWAS, (k) EWASHer, and (l) ReFACTor.

Supplementary Figure 5.

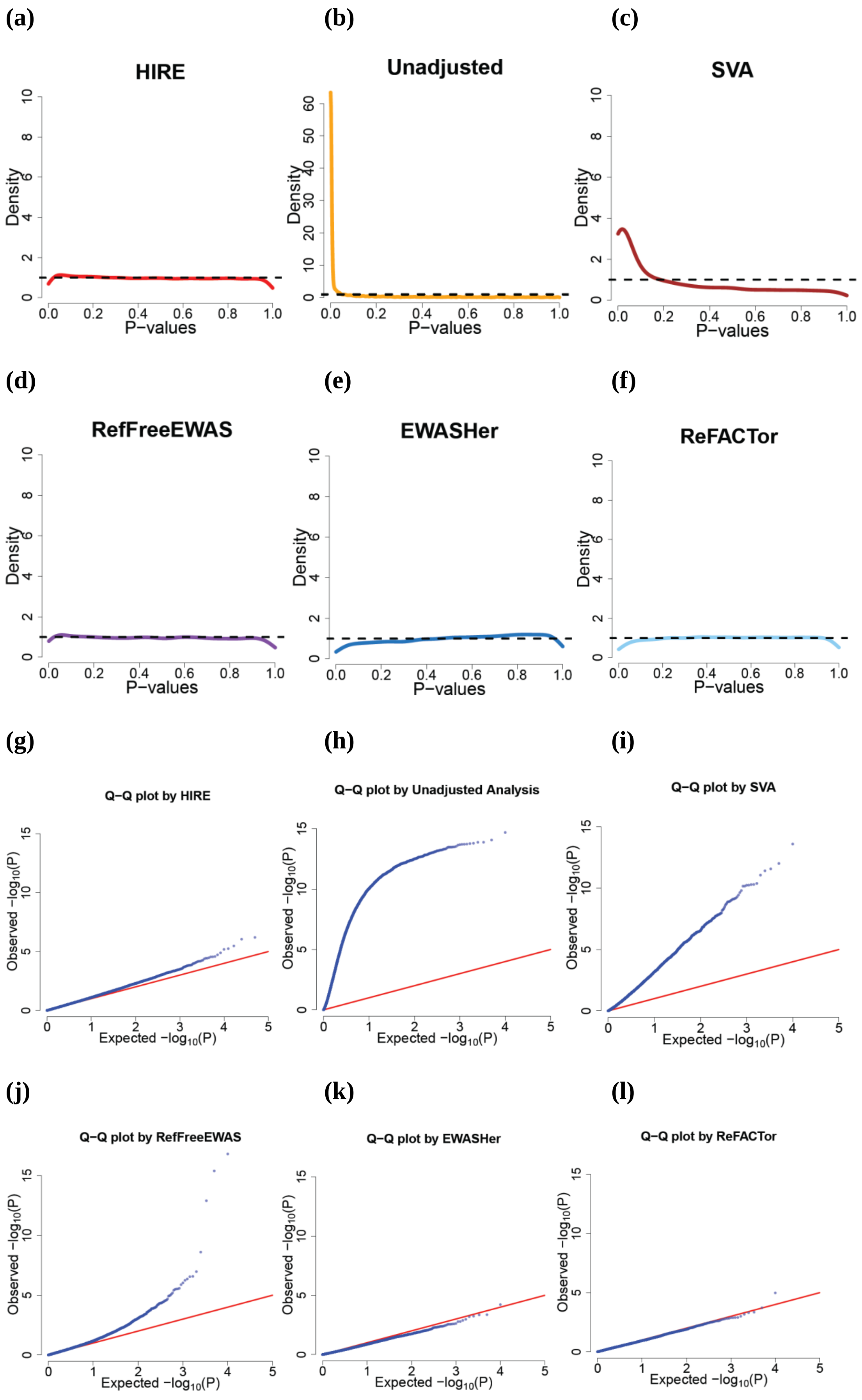

P-value density plots and Q-Q plots for HIRE and the commonly used methods in the “true null”setting with K = 5 and n = 300. P-value density plots for (a) HIRE, (b) unadjusted analysis, (c) SVA, (d) RefFreeEWAS, (e) EWASHer, and (f) ReFACTor. Q-Q plots for (g) HIRE, (h) unadjusted analysis, (i) SVA, (j) RefFreeEWAS, (k) EWASHer, and (l) ReFACTor.

Supplementary Figure 6.

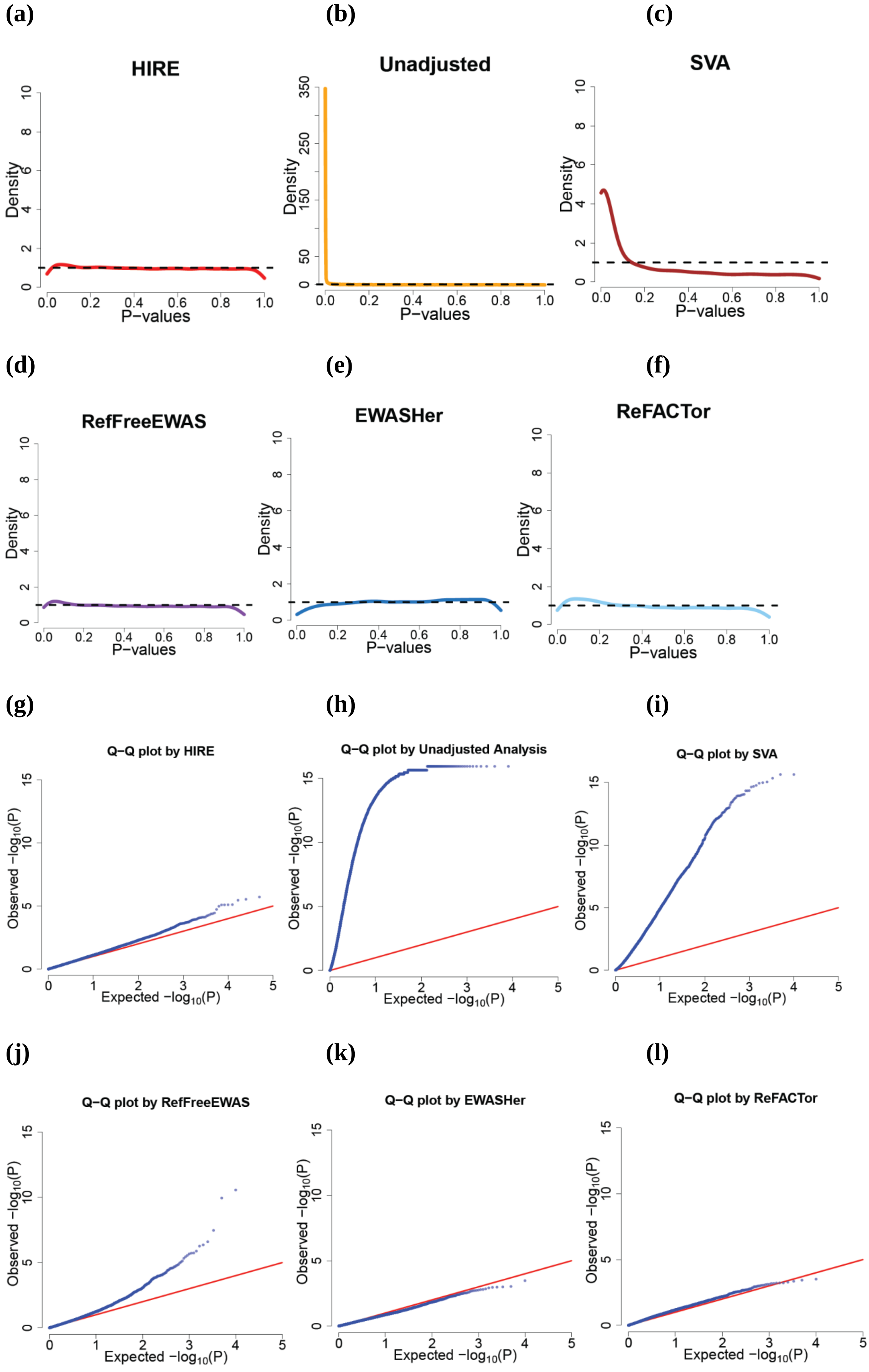

P-value density plots and Q-Q plots for HIRE and the commonly used methods in the “true null”setting with K = 5 and n = 600. P-value density plots for (a) HIRE, (b) unadjusted analysis, (c) SVA, (d) RefFreeEWAS, (e) EWASHer, and (f) ReFACTor. Q-Q plots for (g) HIRE, (h) unadjusted analysis, (i) SVA, (j) RefFreeEWAS, (k) EWASHer, and (l) ReFACTor.

Supplementary Figure 7.

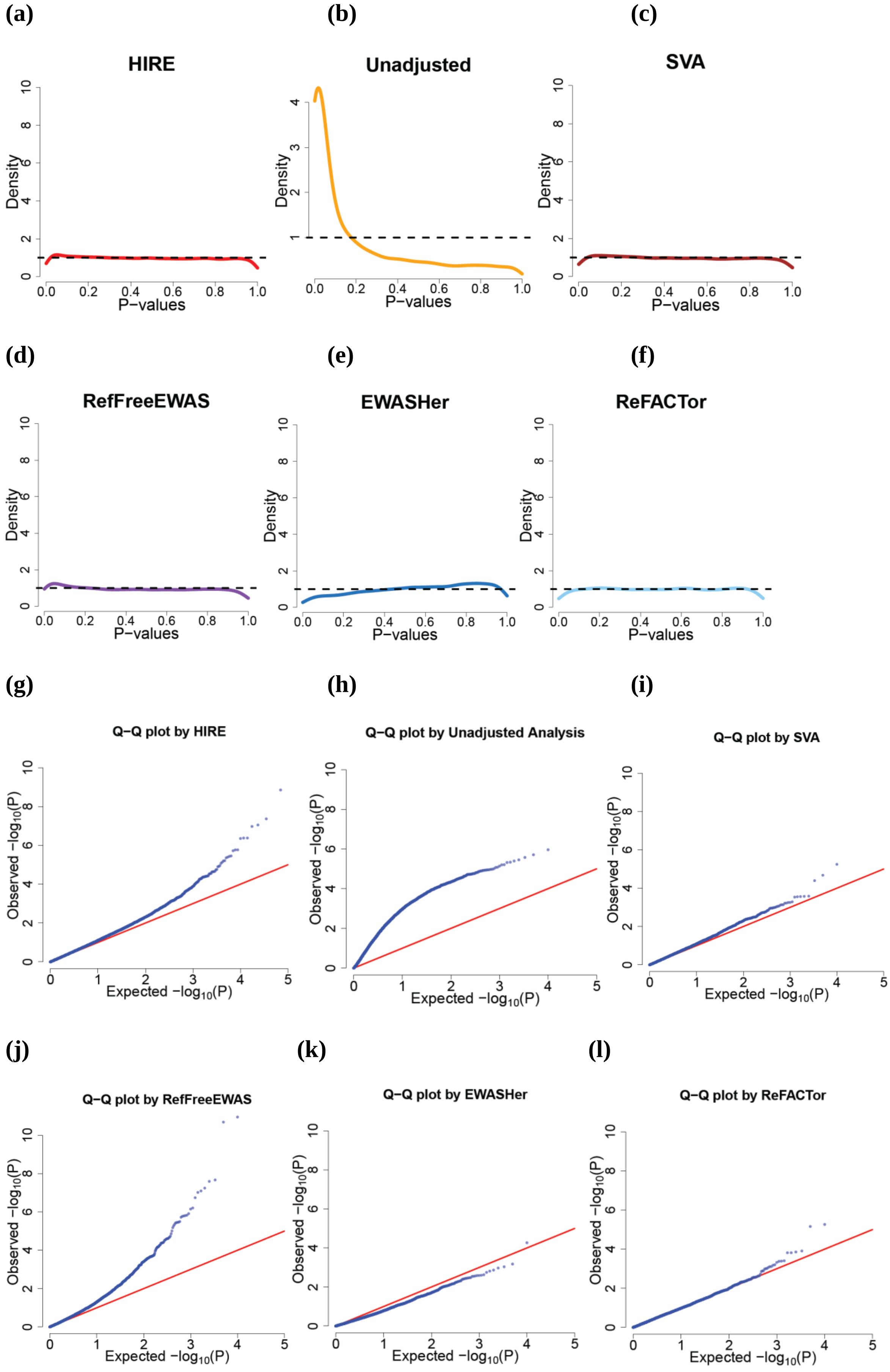

P-value density plots and Q-Q plots for HIRE and the commonly used methods in the “true null”setting with K = 7 and n = 180. P-value density plots for (a) HIRE, (b) unadjusted analysis, (c) SVA, (d) RefFreeEWAS, (e) EWASHer, and (f) ReFACTor. Q-Q plots for (g) HIRE, (h) unadjusted analysis, (i) SVA, (j) RefFreeEWAS, (k) EWASHer, and (l) ReFACTor.

Supplementary Figure 8.

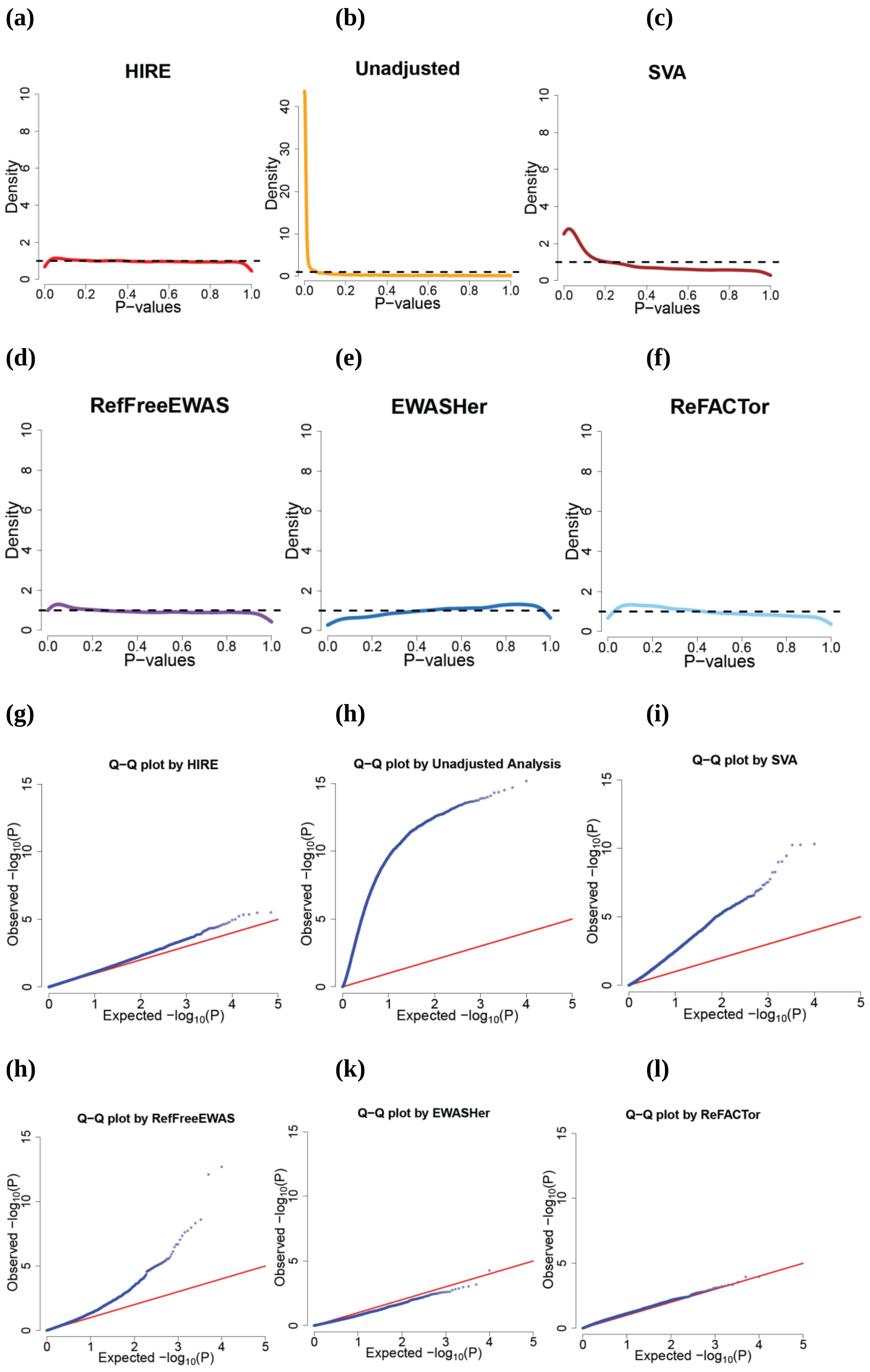

P-value density plots and Q-Q plots for HIRE and the commonly used methods in the “true null”setting with K = 7 and n = 300. P-value density plots for (a) HIRE, (b) unadjusted analysis, (c) SVA, (d) RefFreeEWAS, (e) EWASHer, and (f) ReFACTor. Q-Q plots for (g) HIRE, (h) unadjusted analysis, (i) SVA, (j) RefFreeEWAS, (k) EWASHer, and (l) ReFACTor.

Supplementary Figure 9.

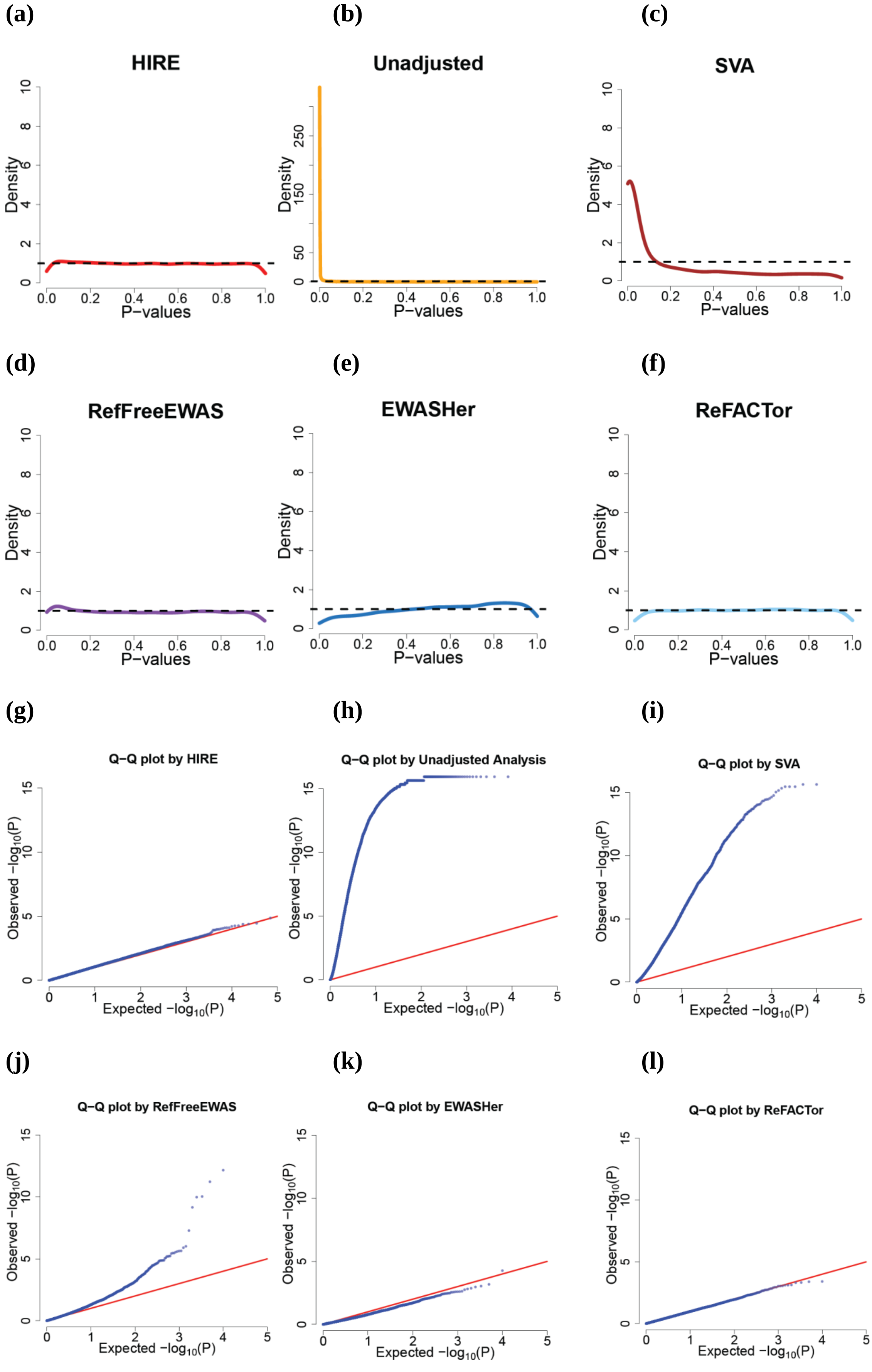

P-value density plots and Q-Q plots for HIRE and the commonly used methods in the “true null”setting with K = 7 and n = 600. P-value density plots for (a) HIRE, (b) unadjusted analysis, (c) SVA, (d) RefFreeEWAS, (e) EWASHer, and (f) ReFACTor. Q-Q plots for (g) HIRE, (h) unadjusted analysis, (i) SVA, (j) RefFreeEWAS, (k) EWASHer, and (l) ReFACTor.

Supplementary Figure 10.

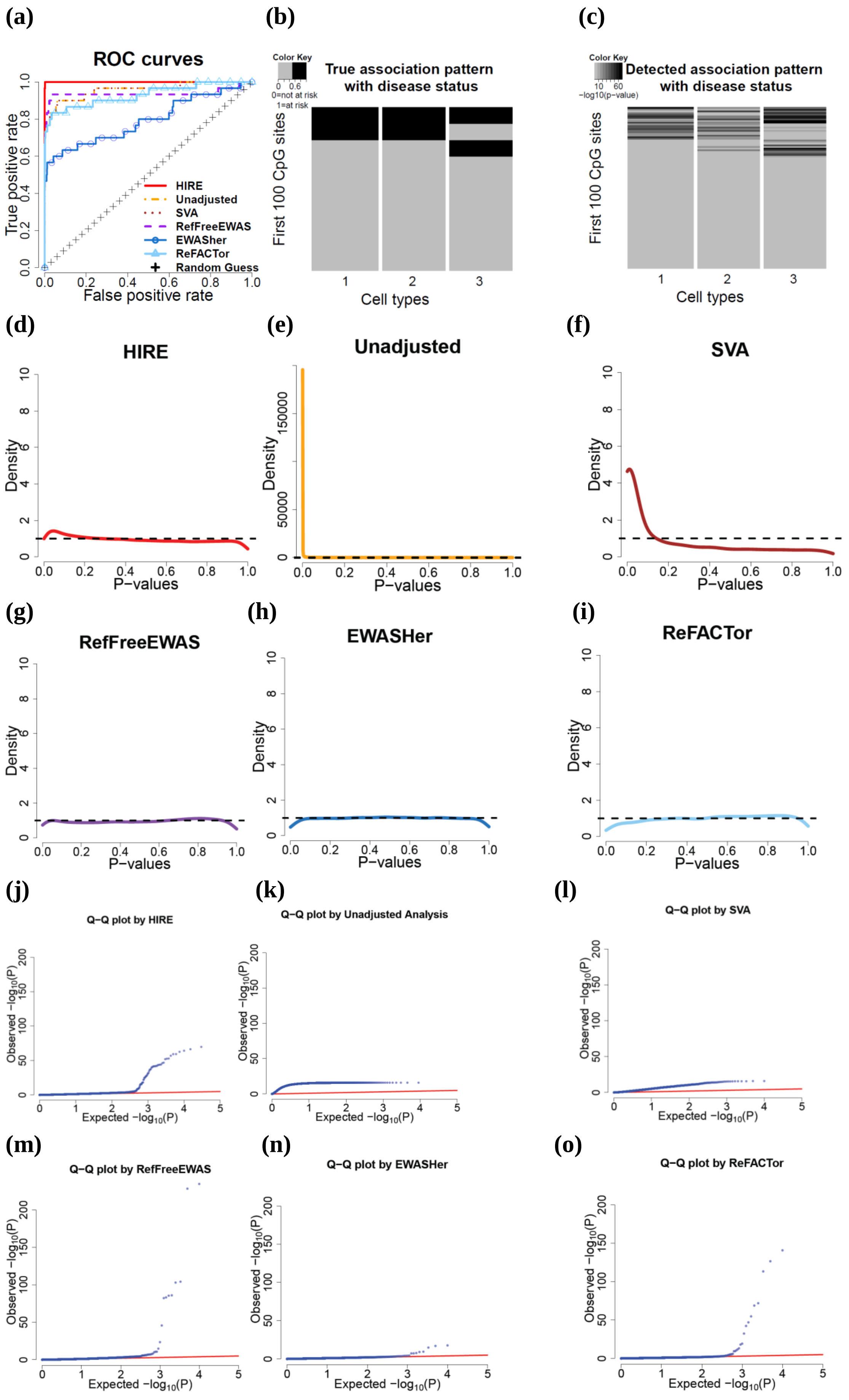

The association detection performance by HIRE and the commonly used methods in the “true alternative”setting with K = 3 and n = 300. (a) The ROC curves of HIRE and the commonly used methods. HIRE has the largest area under the curve among all of the methods. (b) The true cell-type-specific association pattern with disease status for the 10,000 simulated CpG sites, where the columns correspond to cell types and the rows represent the CpG sites. The dark cells correspond to risk-CpG sites, whereas the grey cells are CpG sites not associated with the disease status. (c) The detected cell-type-specific association pattern with disease status by HIRE. The darkness represents $-\log_{10}(p - value)$(d-i) The p-value density plots for association with disease status in the simulation dataset for (d) HIRE, (e) unadjusted analysis, (f) SVA, (g) RefFreeEWAS, (h) EWASHer, and (i) ReFACTor. (j-o) The Q-Q plots for association with disease status for (j) HIRE, (k) unadjusted analysis, (l) SVA, (m) RefFreeEWAS, (n) EWASHer, and (o) ReFACTor.

Supplementary Figure 11.

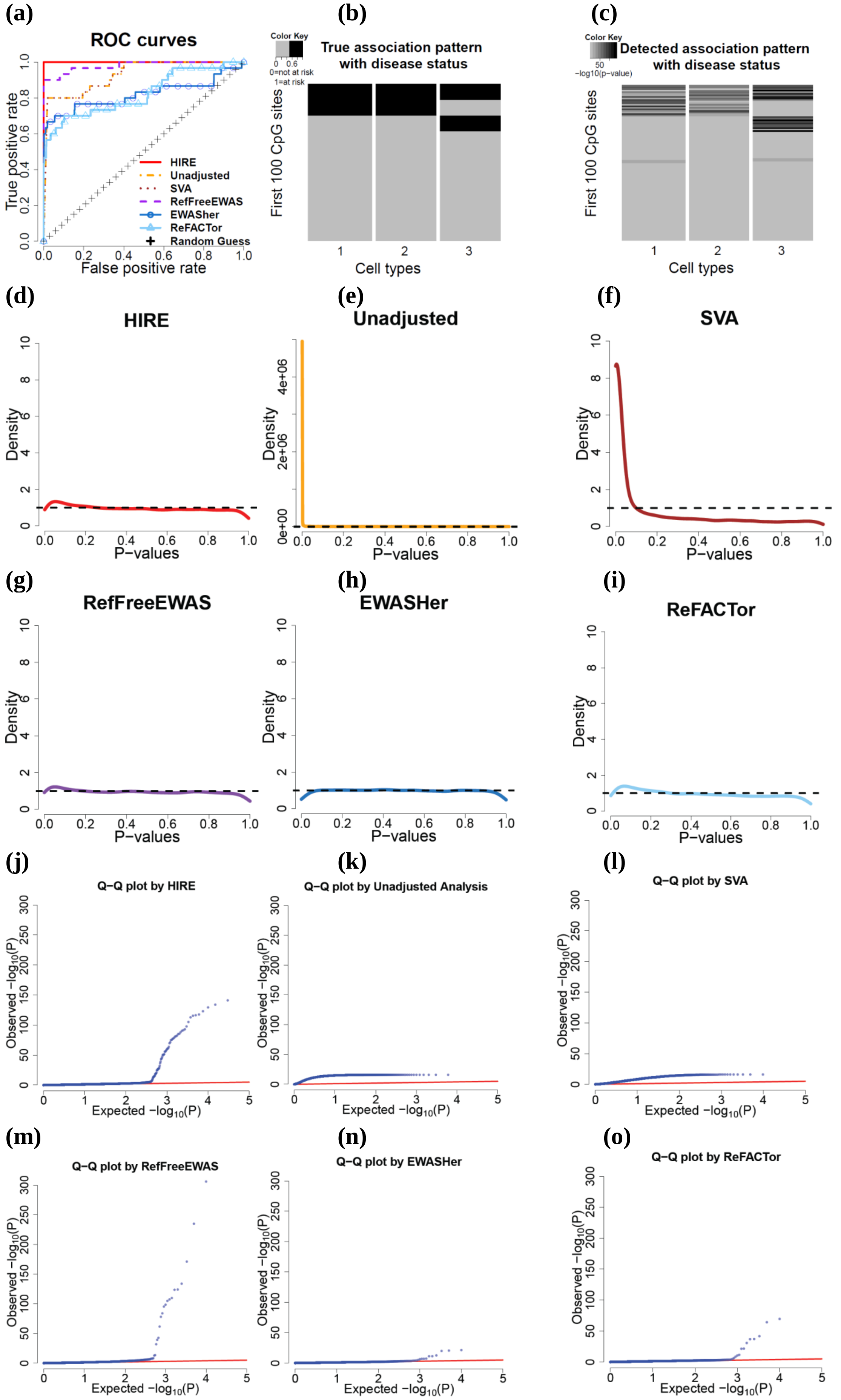

The association detection performance by HIRE and the commonly used methods in the “true alternative”setting with K = 3 and n = 600. (a) The ROC curves of HIRE and the commonly used methods. HIRE has the largest area under the curve among all of the methods. (b) The true cell-type-specific association pattern with disease status for the 10,000 simulated CpG sites, where the columns correspond to cell types and the rows represent the CpG sites. The dark cells correspond to risk-CpG sites, whereas the grey cells are CpG sites not associated with the disease status. (c) The detected cell-type-specific association pattern with disease status by HIRE. The darkness represents $-\log_{10}(p - value)$(d-i) The p-value density plots for association with disease status in the simulation dataset for (d) HIRE, (e) unadjusted analysis, (f) SVA, (g) RefFreeEWAS, (h) EWASHer, and (i) ReFACTor. (j-o) The Q-Q plots for association with disease status for (j) HIRE, (k) unadjusted analysis, (l) SVA, (m) RefFreeEWAS, (n) EWASHer, and (o) ReFACTor.

Supplementary Figure 12.

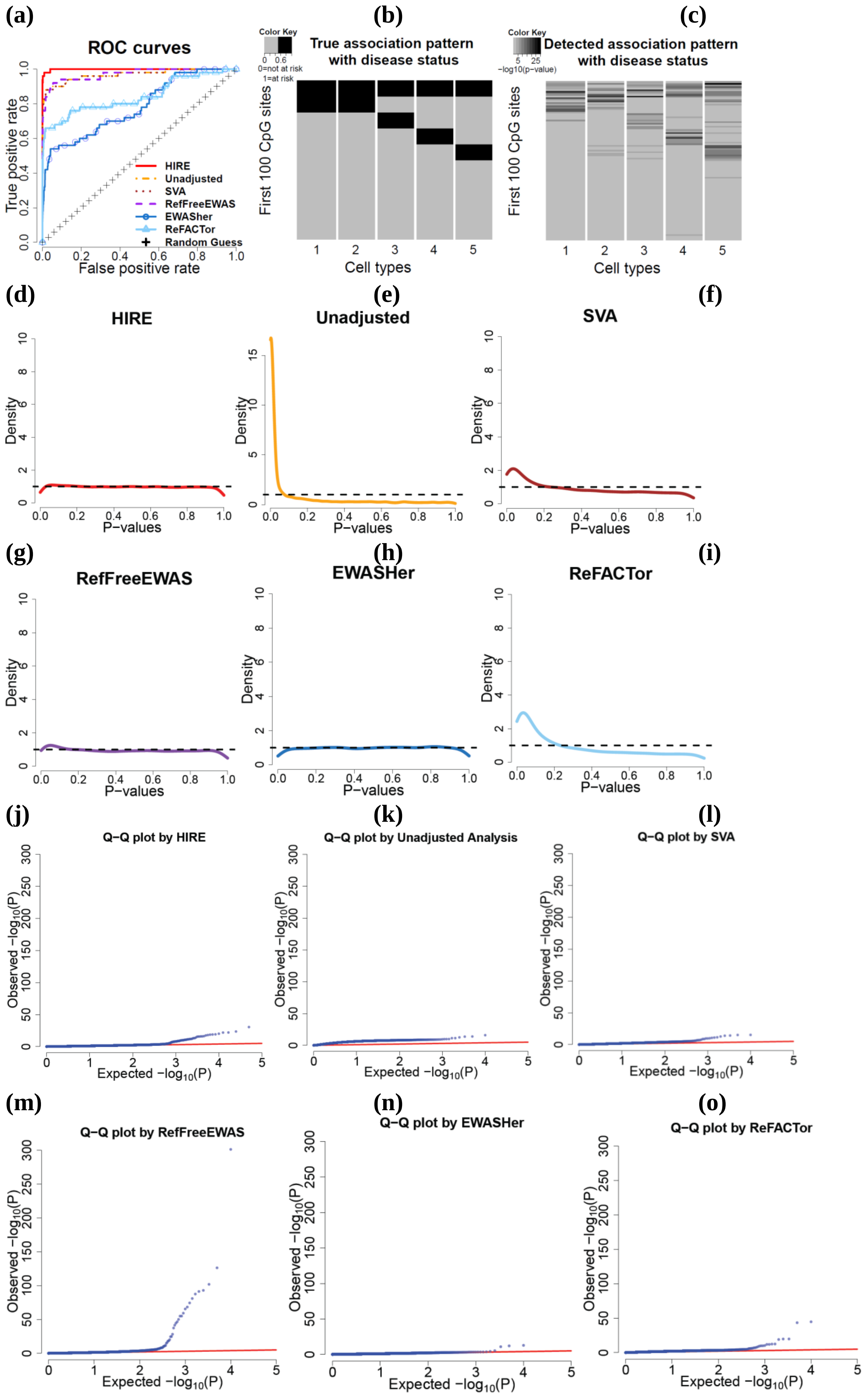

The association detection performance by HIRE and the commonly used methods in the “true alternative”setting with K = 5 and n = 180. (a) The ROC curves of HIRE and the commonly used methods. HIRE has the largest area under the curve among all of the methods. (b) The true cell-type-specific association pattern with disease status for the 10,000 simulated CpG sites, where the columns correspond to cell types and the rows represent the CpG sites. The dark cells correspond to risk-CpG sites, whereas the grey cells are CpG sites not associated with the disease status. (c) The detected cell-type-specific association pattern with disease status by HIRE. The darkness represents $-\log_{10}(p-value)$(d-i) The p-value density plots for association with disease status in the simulation dataset for (d) HIRE, (e) unadjusted analysis, (f) SVA, (g) RefFreeEWAS, (h) EWASHer, and (i) ReFACTor. (j-o) The Q-Q plots for association with disease status for (j) HIRE, (k) unadjusted analysis, (l) SVA, (m) RefFreeEWAS, (n) EWASHer, and (o) ReFACTor.

Supplementary Figure 13.

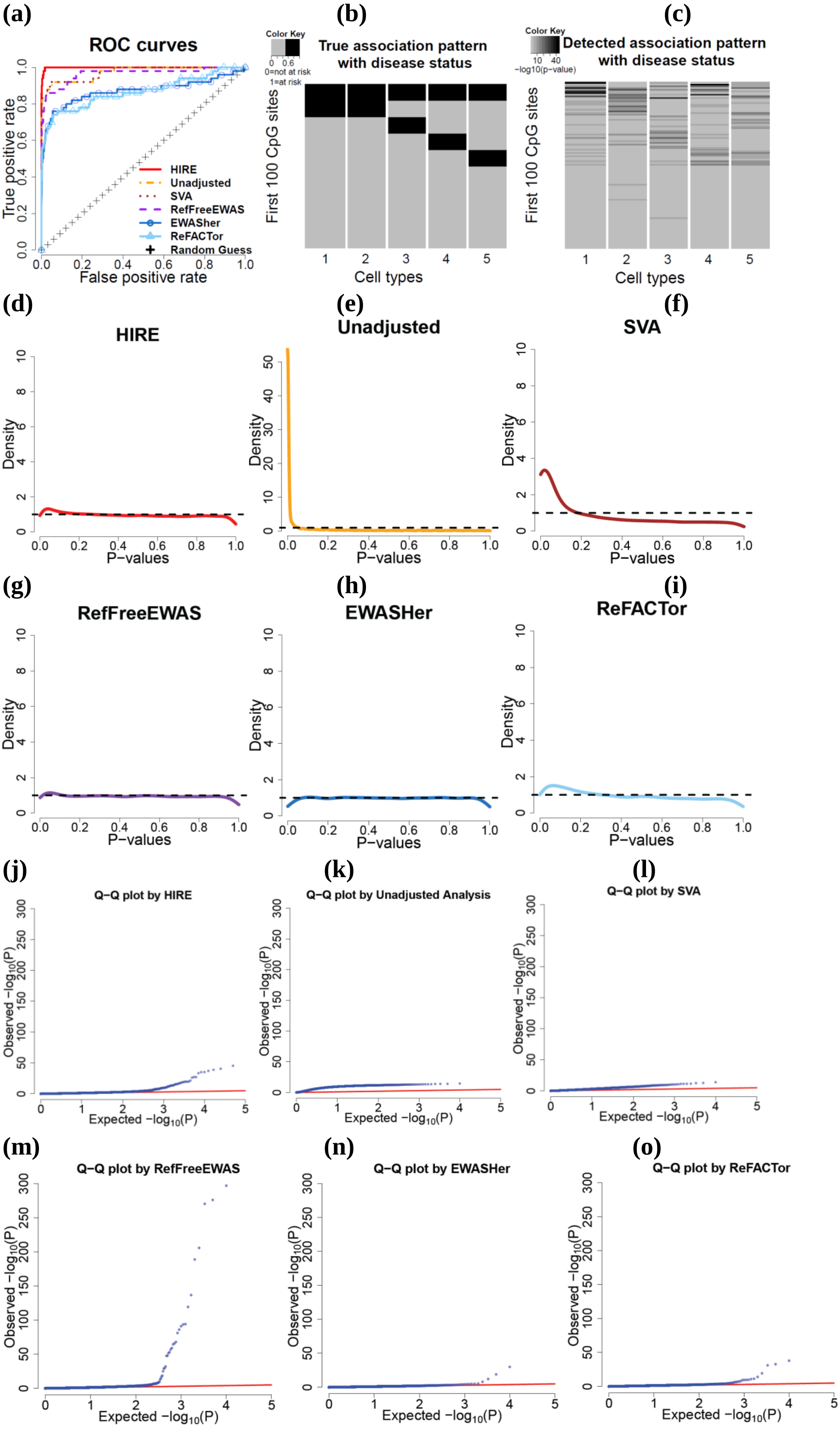

The association detection performance by HIRE and the commonly used methods in the "true alternative" simulation study with K = 5 and n = 300. (a) The ROC curves of HIRE and the commonly used methods. HIRE has the largest area under the curve among all of the methods. (b) The true cell-type-specific association pattern with disease status for the 10,000 simulated CpG sites, where the columns correspond to cell types and the rows represent the CpG sites. The dark cells correspond to risk-CpG sites, whereas the grey cells are CpG sites not associated with the disease status. (c) The detected cell-type-specific association pattern with disease status by HIRE. The darkness represents $-\log_{10}(p - value)$(d-i) The p-value density plots for association with disease status in the simulation dataset for (d) HIRE, (e) unadjusted analysis, (f) SVA, (g) RefFreeEWAS, (h) EWASHer, and (i) ReFACTor. (j-o) The Q-Q plots for association with disease status for (j) HIRE, (k) unadjusted analysis, (l) SVA, (m) RefFreeEWAS, (n) EWASHer, and (o) ReFACTor.

Supplementary Figure 14.

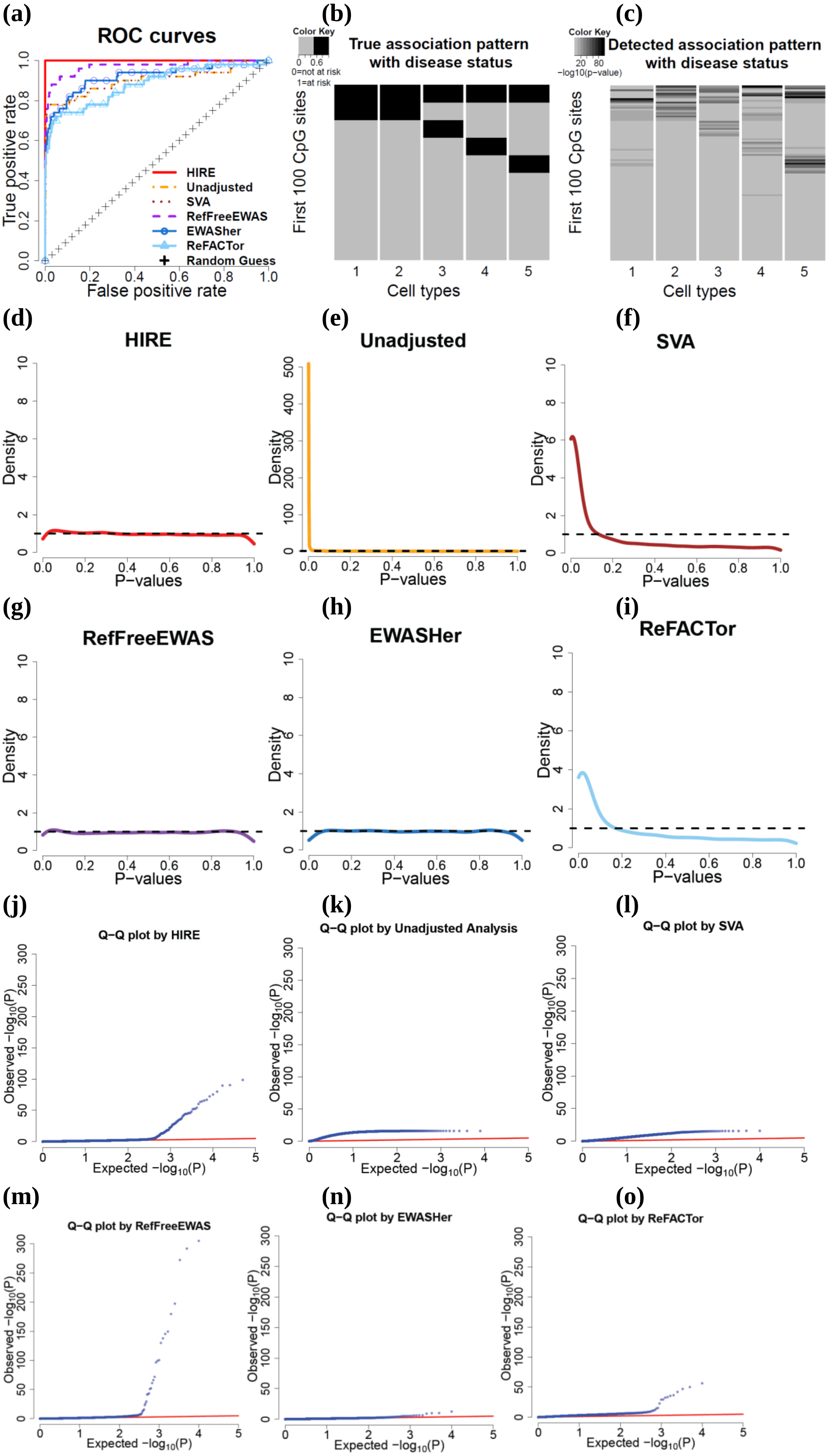

The association detection performance by HIRE and the commonly used methods in the “true alternative”setting with K = 5 and n = 600. (a) The ROC curves of HIRE and the commonly used methods. HIRE has the largest area under the curve among all of the methods. (b) The true cell-type-specific association pattern with disease status for the 10,000 simulated CpG sites, where the columns correspond to cell types and the rows represent the CpG sites. The dark cells correspond to risk-CpG sites, whereas the grey cells are CpG sites not associated with the disease status. (c) The detected cell-type-specific association pattern with disease status by HIRE. The darkness represents $-\log_{10}(p - value)$(d-i) The p-value density plots for association with disease status in the simulation dataset for (d) HIRE, (e) unadjusted analysis, (f) SVA, (g) RefFreeEWAS, (h) EWASHer, and (i) ReFACTor. (j-o) The Q-Q plots for association with disease status for (j) HIRE, (k) unadjusted analysis, (l) SVA, (m) RefFreeEWAS, (n) EWASHer, and (o) ReFACTor.

Supplementary Figure 15.

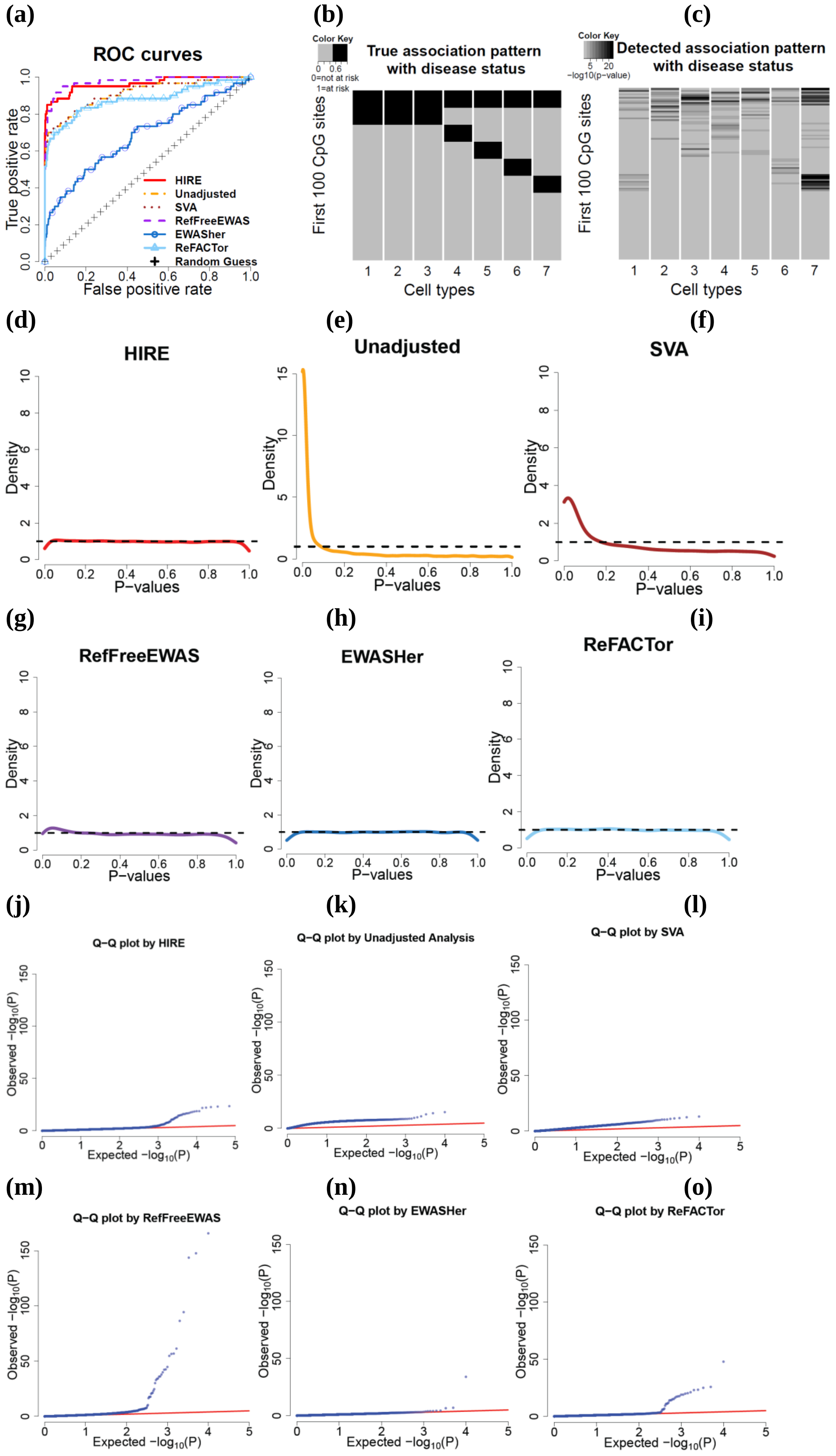

The association detection performance by HIRE and the commonly used methods in the “true alternative”setting with K = 7 and n = 180. (a) The ROC curves of HIRE and the commonly used methods. HIRE has the largest power given the false positive rate less than 0.05. (b) The true cell-type-specific association pattern with disease status for the 10,000 simulated CpG sites, where the columns correspond to cell types and the rows represent the CpG sites. The dark cells correspond to risk-CpG sites, whereas the grey cells are CpG sites not associated with the disease status. (c) The detected cell-type-specific association pattern with disease status by HIRE. The darkness represents $-\log_{10}(p - value)$(d-i) The p-value density plots for association with disease status in the simulation dataset for (d) HIRE, (e) unadjusted analysis, (f) SVA, (g) RefFreeEWAS, (h) EWASHer, and (i) ReFACTor. (j-o) The Q-Q plots for association with disease status for (j) HIRE, (k) unadjusted analysis, (l) SVA, (m) RefFreeEWAS, (n) EWASHer, and (o) ReFACTor.

Supplementary Figure 16.

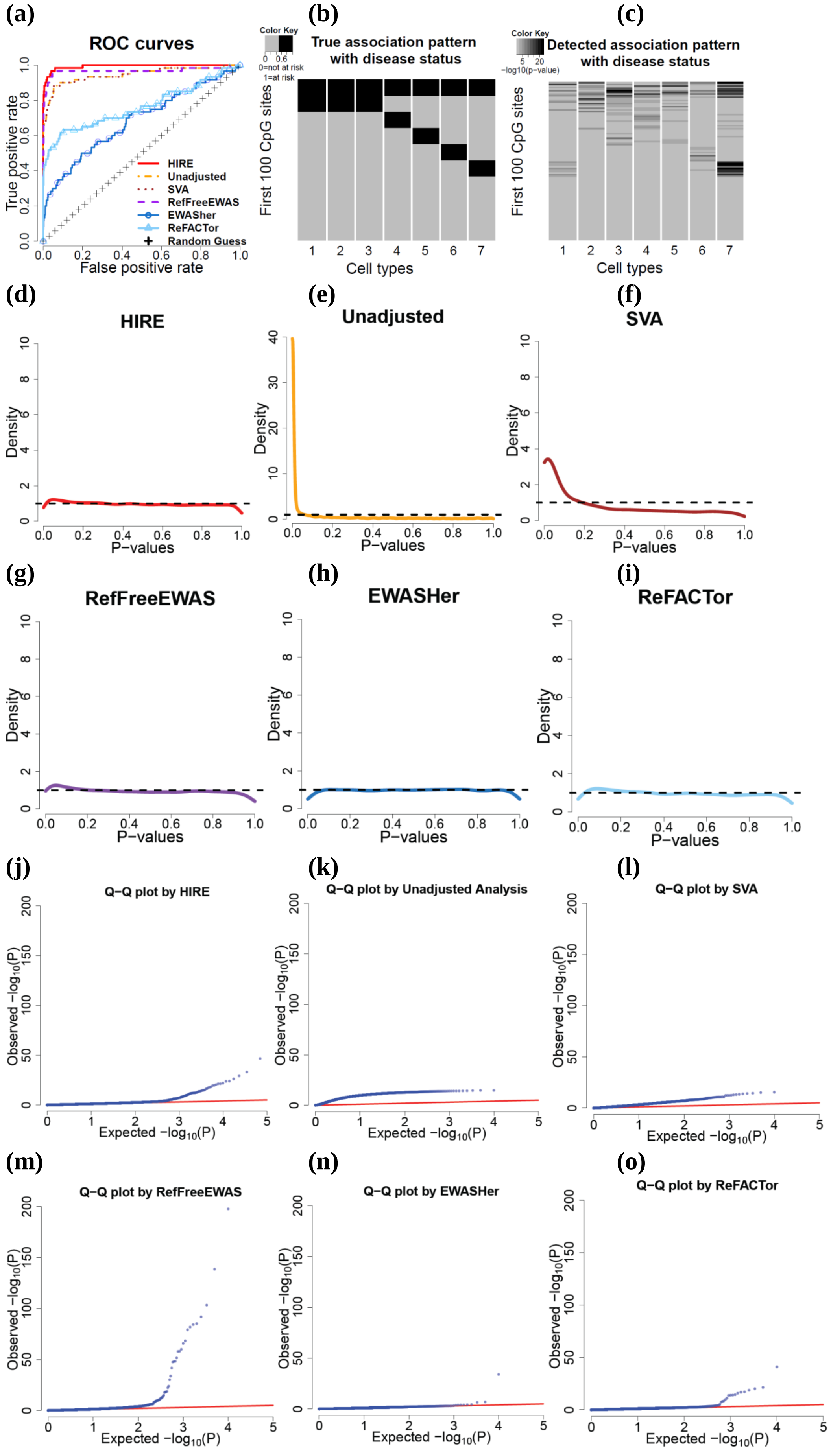

The association detection performance by HIRE and the commonly used methods in the “true alternative” setting with K = 7 and n = 300. (a) The ROC curves of HIRE and the commonly used methods. HIRE has the largest area under the curve among all of the methods. (b) The true cell-type-specific association pattern with disease status for the 10,000 simulated CpG sites, where the columns correspond to cell types and the rows represent the CpG sites. The dark cells correspond to risk-CpG sites, whereas the grey cells are CpG sites not associated with the disease status. (c) The detected cell-type-specific association pattern with disease status by HIRE. The darkness represents $-\log_{10}(p - value)$(d-i) The p-value density plots for association with disease status in the simulation dataset for (d) HIRE, (e) unadjusted analysis, (f) SVA, (g) RefFreeEWAS, (h) EWASHer, and (i) ReFACTor. (j-o) The Q-Q plots for association with disease status for (j) HIRE, (k) unadjusted analysis, (l) SVA, (m) RefFreeEWAS, (n) EWASHer, and (o) ReFACTor.

Supplementary Figure 17.

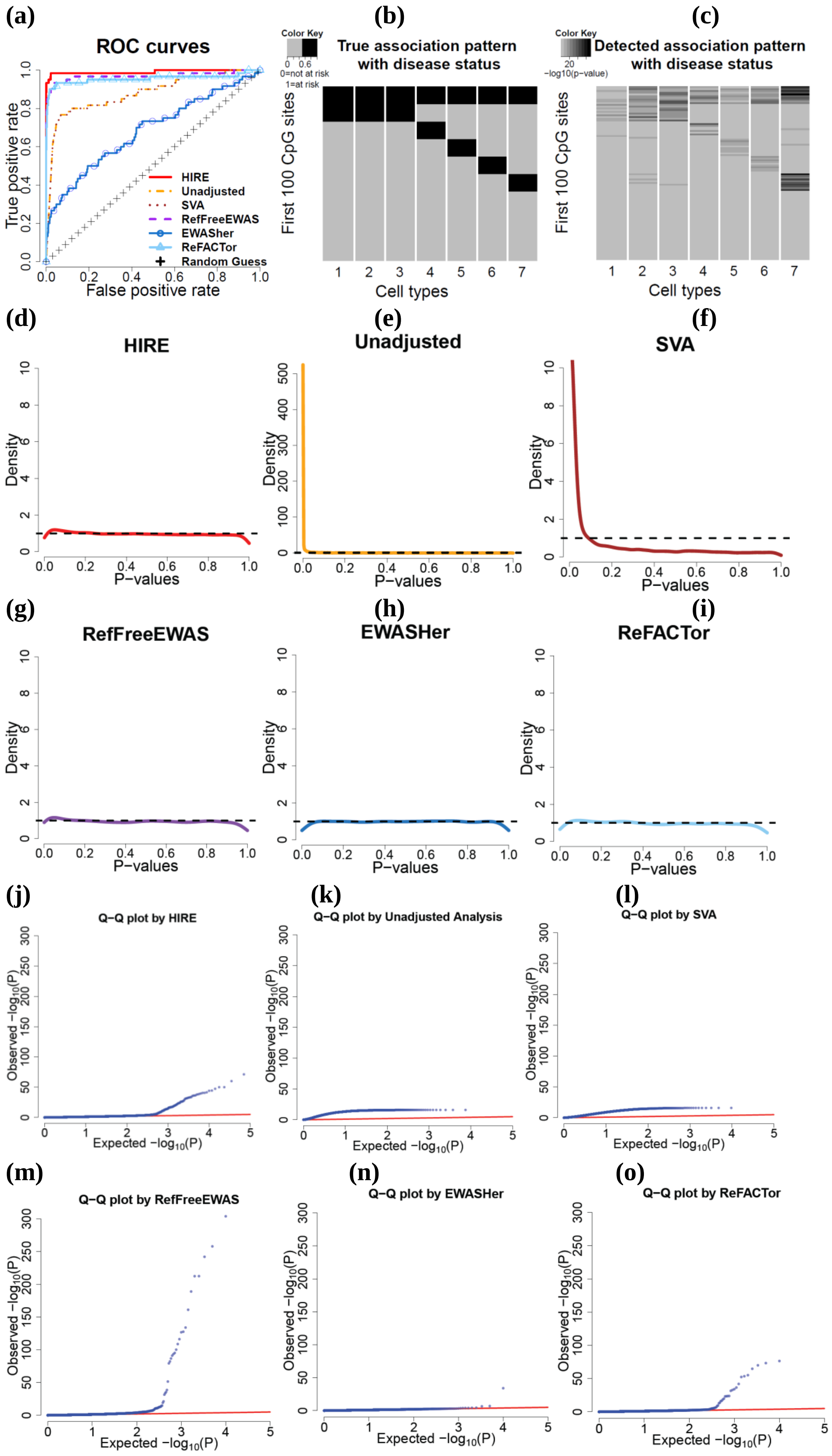

The association detection performance by HIRE and the commonly used methods in the "true alternative"setting with K = 7 and n = 600. (a) The ROC curves of HIRE and the commonly used methods. HIRE has the largest area under the curve among all of the methods. (b) The true cell-type-specific association pattern with disease status for the 10,000 simulated CpG sites, where the columns correspond to cell types and the rows represent the CpG sites. The dark cells correspond to risk-CpG sites, whereas the grey cells are CpG sites not associated with the disease status. (c) The detected cell-type-specific association pattern with disease status by HIRE. The darkness represents $-\log_{10}(p - value)$(d-i) The p-value density plots for association with disease status in the simulation dataset for (d) HIRE, (e) unadjusted analysis, (f) SVA, (g) RefFreeEWAS, (h) EWASHer, and (i) ReFACTor. (j-o) The Q-Q plots for association with disease status for (j) HIRE, (k) unadjusted analysis, (l) SVA, (m) RefFreeEWAS, (n) EWASHer, and (o) ReFACTor.

Supplementary Figure 18.

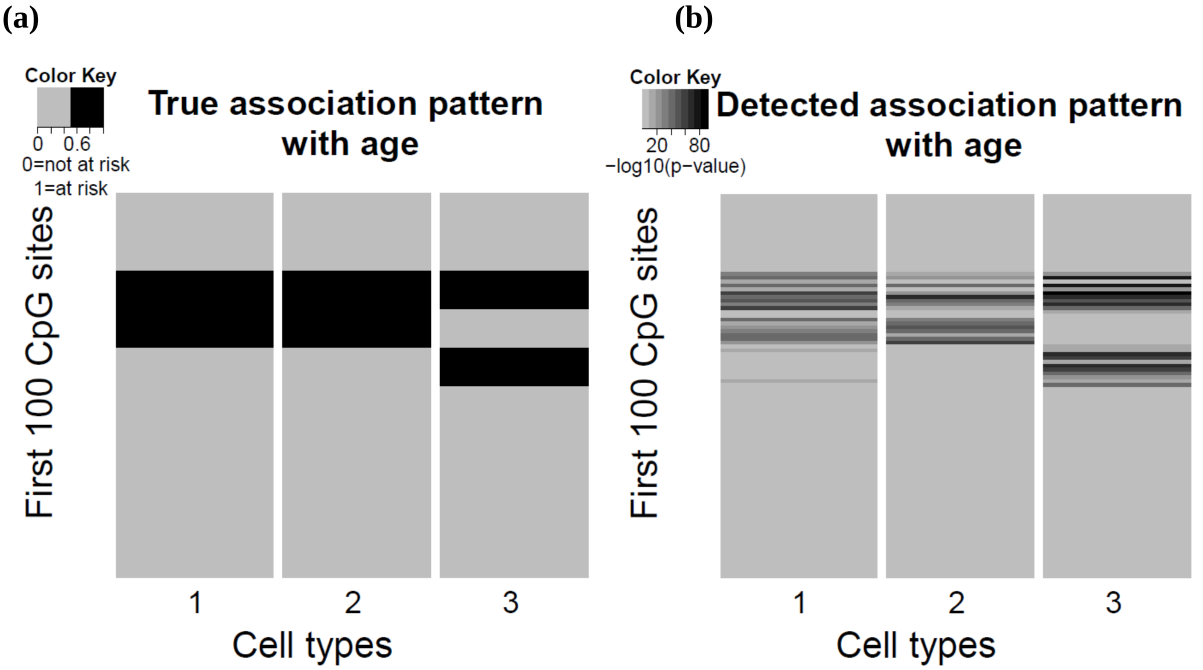

The underlying true cell-type-specific association pattern with age and its detectedassociation pattern by HIRE in the simulated dataset—"true alternative" with K=3 and n=180. In both panels, the rows correspond to the CpG sites, and the columns represent the cell types.**(a)** The underlying true cell-type-specific association pattern with age. The dark cells represent the CpG sites at risk—associated with age. The grey cellscorrespond to CpG sites not at risk—not associated with age. **(b)** The detected association pattern with age by HIRE. In this heatmap, each cell's darkness degree represents the significance level at the scaleof $-\log_{10}(p - value)$.

Supplementary Figure 19.

(a) **(b)**

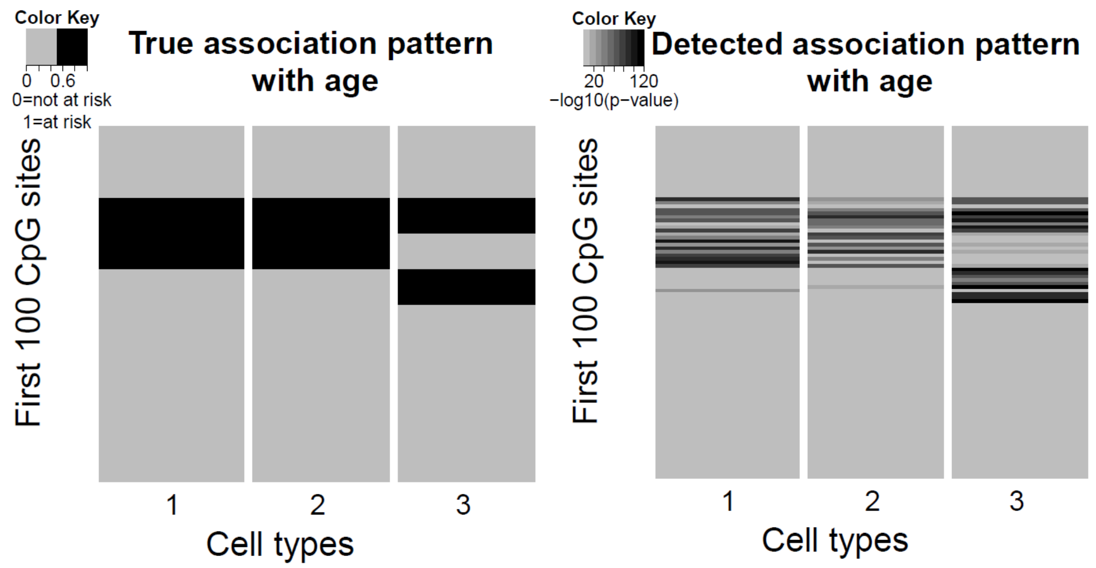

The underlying true cell-type-specific association pattern with age and its detectedassociation pattern by HIRE in the simulated dataset—"true alternative" with K=3 and n=300. In both panels, the rows correspond to the CpG sites, and the columns represent the cell types.(a) The underlying true cell-type-specific association pattern with age. The dark cells represent the CpG sites at risk—associated with age. The grey cellscorrespond to CpG sites not at risk—not associated with age. (b) The detected association pattern with age by HIRE. In this heatmap, each cell's darkness degree represents the significance level at the scaleof $-\log_{10}(p-value)$.

Supplementary Figure 20.

(a) **(b)**

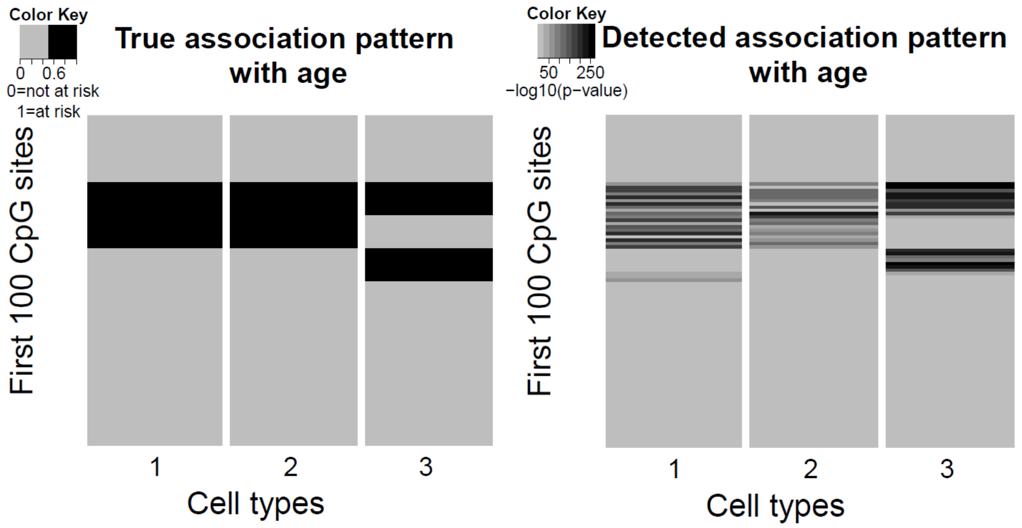

The underlying true cell-type-specific association pattern with age and its detectedassociation pattern by HIRE in the simulated dataset—“true alternative” with K=3 and n=600. In both panels, the rows correspond to the CpG sites, and the columns represent the cell types.**(a)** The underlying true cell-type-specific association pattern with age. The dark cells represent the CpG sites at risk—associated with age. The grey cellscorrespond to CpG sites not at risk—not associated with age. **(b)** The detected association pattern with age by HIRE. In this heatmap, each cell's darkness degree represents the significance level at the scaleof $-\log_{10}(p - value)$.

Supplementary Figure 21.

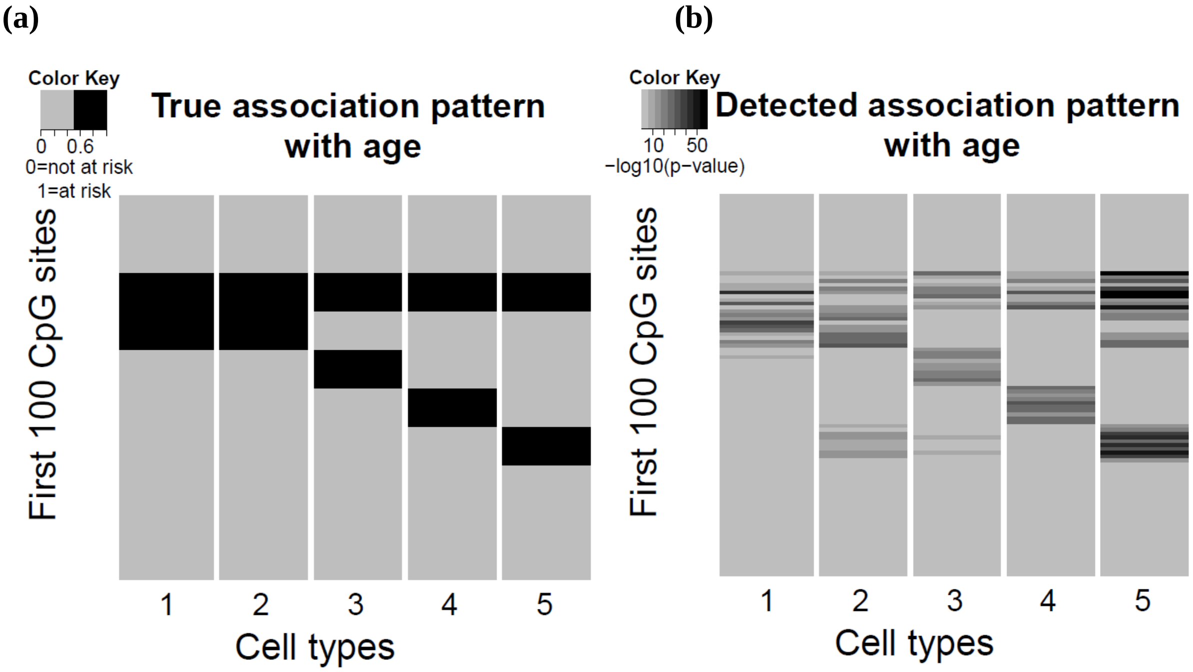

The underlying true cell-type-specific association pattern with age and its detectedassociation pattern by HIRE in the simulated dataset—“true alternative” with K=5 and n=180. In both panels, the rows correspond to the CpG sites, and the columns represent the cell types.(a) The underlying true cell-type-specific association pattern with age. The dark cells represent the CpG sites at risk—associated with age. The grey cellscorrespond to CpG sites not at risk—not associated with age. (b) The detected association pattern with age by HIRE. In this heatmap, each cell's darkness degree represents the significance level at the scaleof $-\log_{10}(p - value)$.

Supplementary Figure 22.

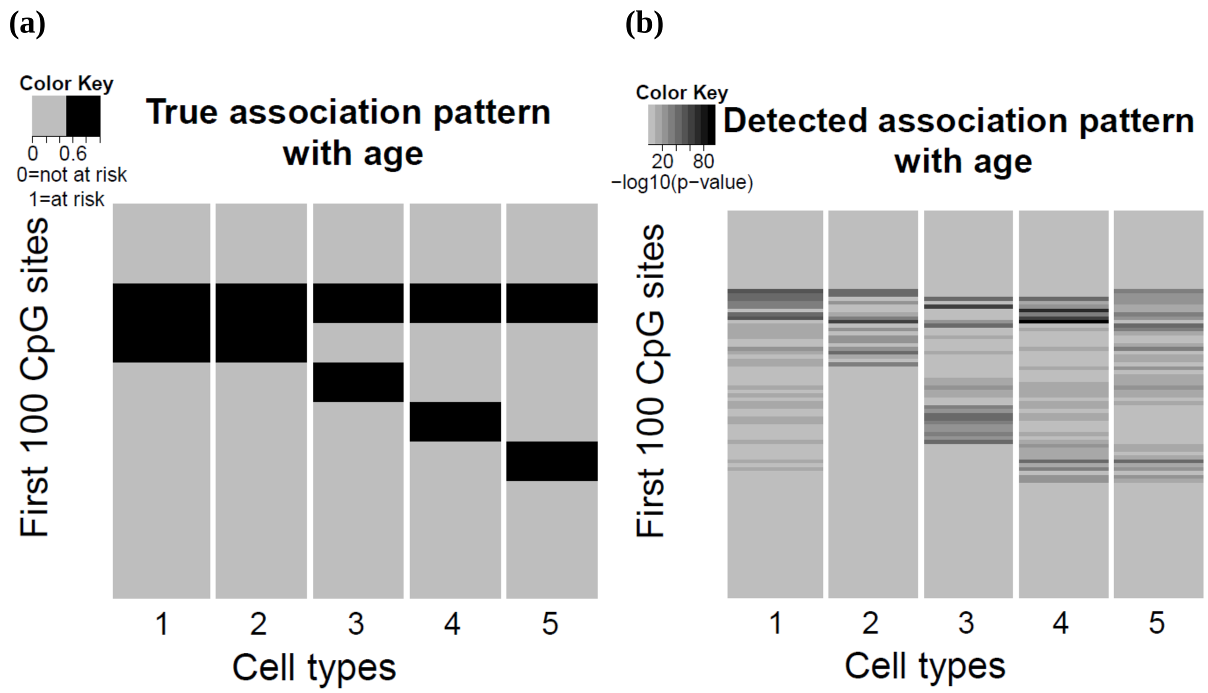

The underlying true cell-type-specific association pattern with age and its detectedassociation pattern by HIRE in the simulated dataset—“true alternative” with K=5 and n=300. In both panels, the rows correspond to the CpG sites, and the columns represent the cell types.(a) The underlying true cell-type-specific association pattern with age. The dark cells represent the CpG sites at risk—associated with age. The grey cellscorrespond to CpG sites not at risk—not associated with age. (b) The detected association pattern with age by HIRE. In this heatmap, each cell's darkness degree represents the significance level at the scaleof $-\log_{10}(p - value)$.

Supplementary Figure 23.

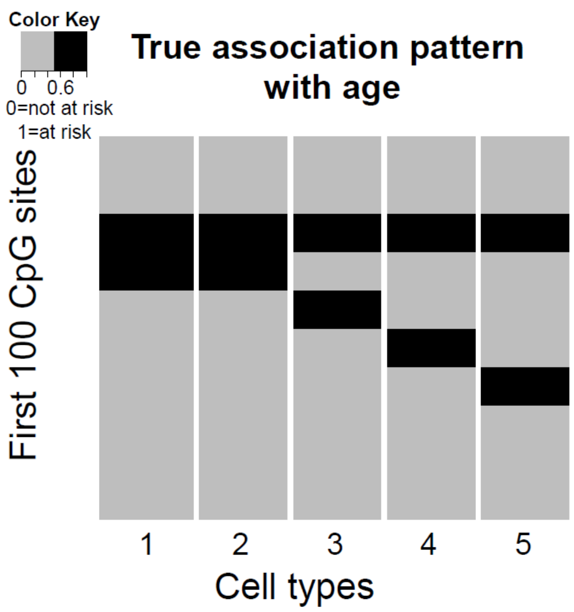

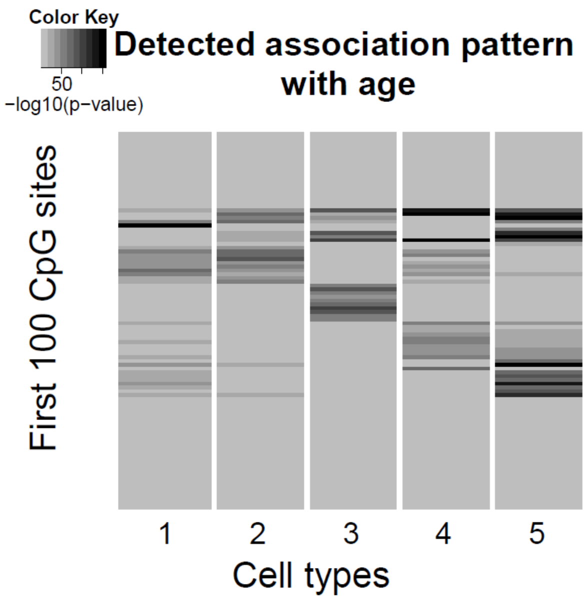

The underlying true cell-type-specific association pattern with age and its detectedassociation pattern by HIRE in the simulated dataset—“true alternative” with K=5 and n=600. In both panels, the rows correspond to the CpG sites, and the columns represent the cell types.(a) The underlying true cell-type-specific association pattern with age. The dark cells represent the CpG sites at risk—associated with age. The grey cellscorrespond to CpG sites not at risk—not associated with age. (b) The detected association pattern with age by HIRE. In this heatmap, each cell's darkness degree represents the significance level at the scaleof $-\log_{10}(p - value)$.

Supplementary Figure 24.

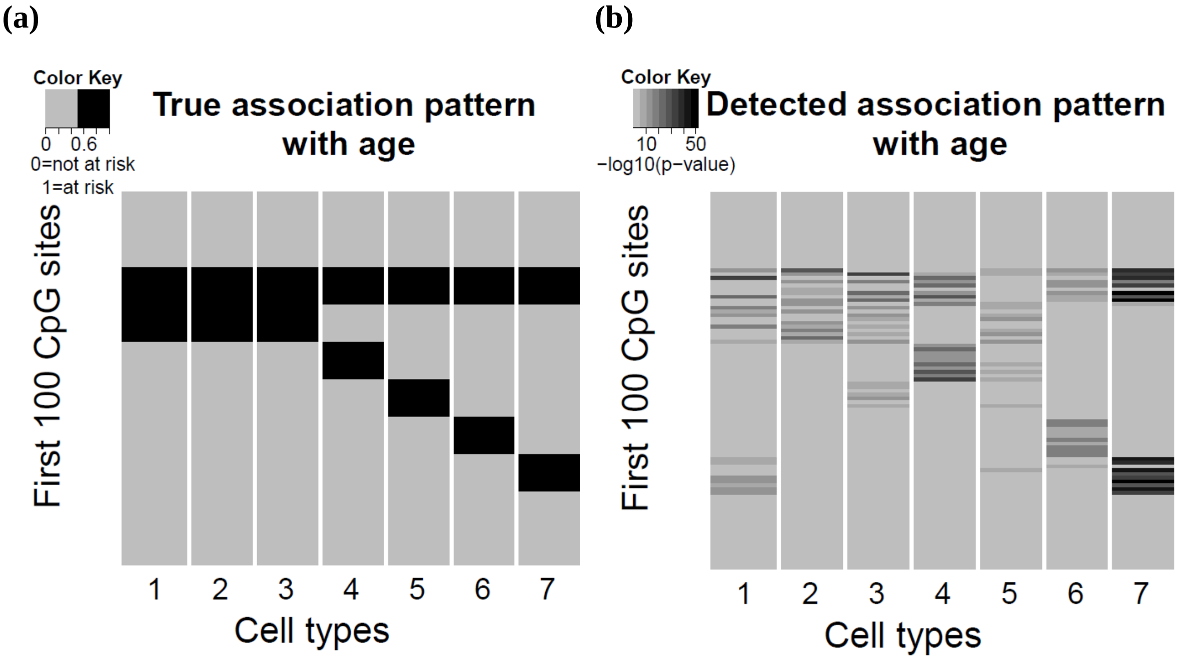

The underlying true cell-type-specific association pattern with age and its detectedassociation pattern by HIRE in the simulated dataset—"true alternative" with K=7 and n=180. In both panels, the rows correspond to the CpG sites, and the columns represent the cell types.(a) The underlying true cell-type-specific association pattern with age. The dark cells represent the CpG sites at risk—associated with age. The grey cellscorrespond to CpG sites not at risk—not associated with age. (b) The detected association pattern with age by HIRE. In this heatmap, each cell's darkness degree represents the significance level at the scaleof $-\log_{10}(p-value)$.

Supplementary Figure 25.

(a) **(b)**

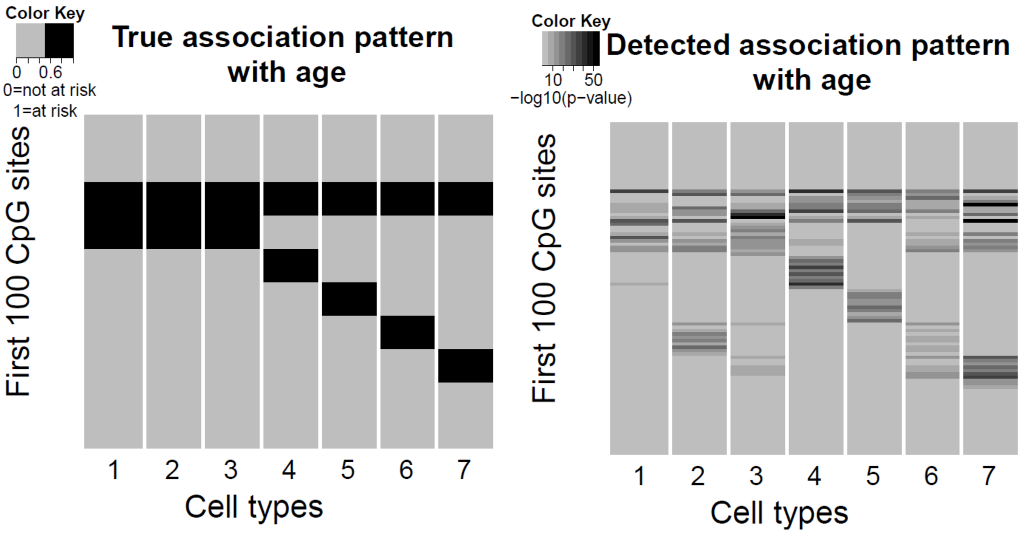

The underlying true cell-type-specific association pattern with age and its detectedassociation pattern by HIRE in the simulated dataset—"true alternative" with K=7 and n=300. In both panels, the rows correspond to the CpG sites, and the columns represent the cell types.(a) The underlying true cell-type-specific association pattern with age. The dark cells represent the CpG sites at risk—associated with age. The grey cellscorrespond to CpG sites not at risk—not associated with age. (b) The detected association pattern with age by HIRE. In this heatmap, each cell's darkness degree represents the significance level at the scaleof $-\log_{10}(p - value)$.

Supplementary Figure 26.

(a) **(b)**

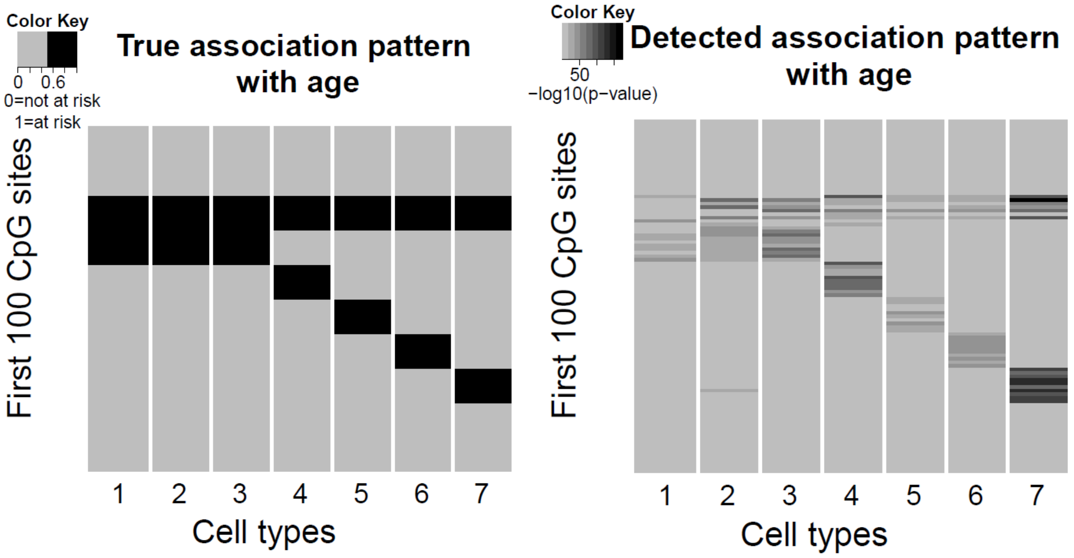

The underlying true cell-type-specific association pattern with age and its detectedassociation pattern by HIRE in the simulated dataset—"true alternative" with K=7 and n=600. In both panels, the rows correspond to the CpG sites, and the columns represent the cell types.(a) The underlying true cell-type-specific association pattern with age. The dark cells represent the CpG sites at risk—associated with age. The grey cellscorrespond to CpG sites not at risk—not associated with age. (b) The detected association pattern with age by HIRE. In this heatmap, each cell's darkness degree represents the significance level at the scaleof $-\log_{10}(p - value)$.

Supplementary Figure 27.

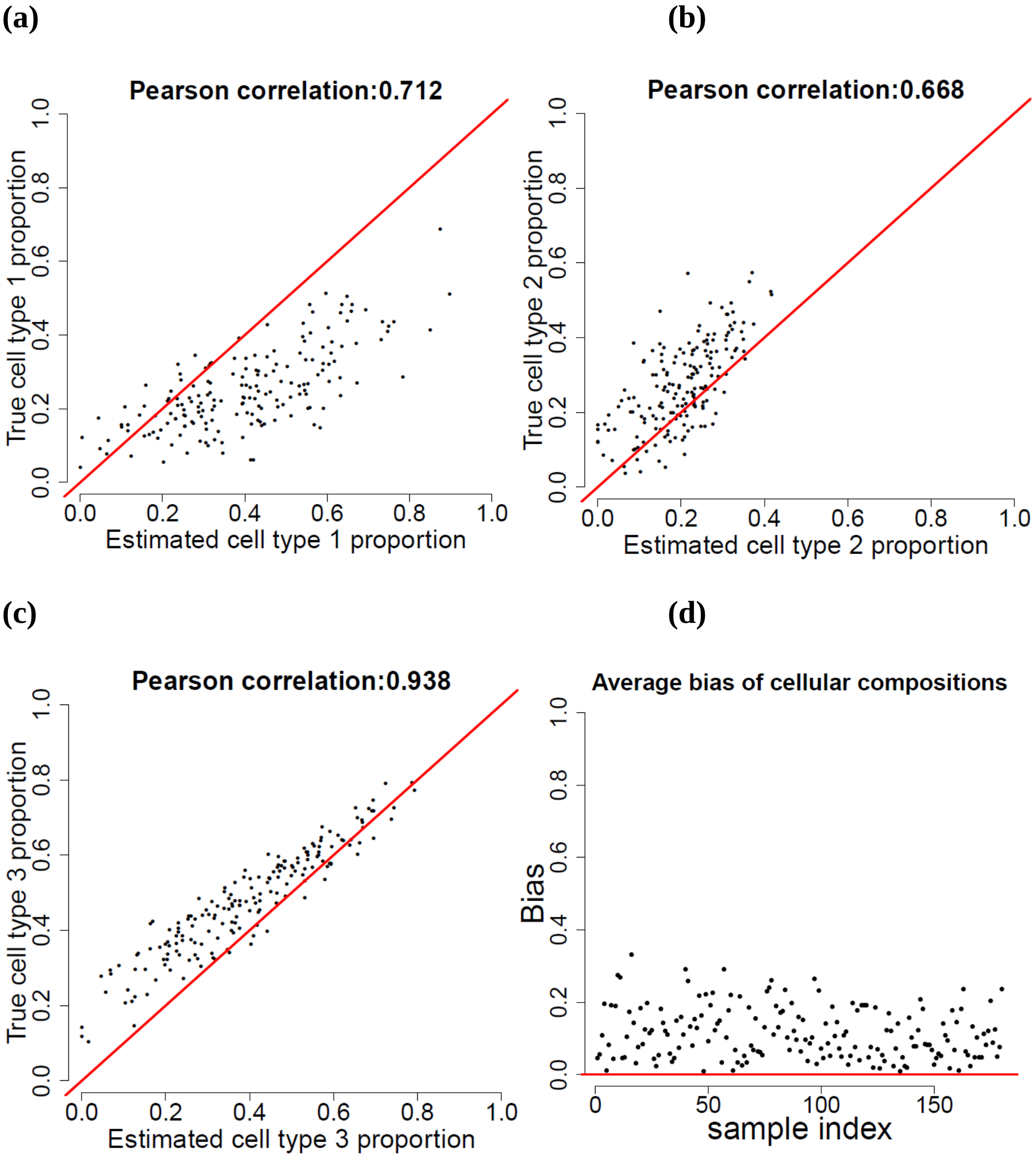

In the "true null" case with K=3 and n=180, (a-c) the scatter plots for the estimated cell proportions by HIRE and the underlying truth for (a) cell type 1; (b) cell type 2; and (c) cell type 3. (d) The average biasplot for the cellular compositions, where the y axis is $\frac{1}{K}\sum_{k=1}^{K}|\widehat{p}_{ki} - p_{ki}|$ and the x axis is the sample index i for$i = 1, \ldots, n$.

Supplementary Figure 28.

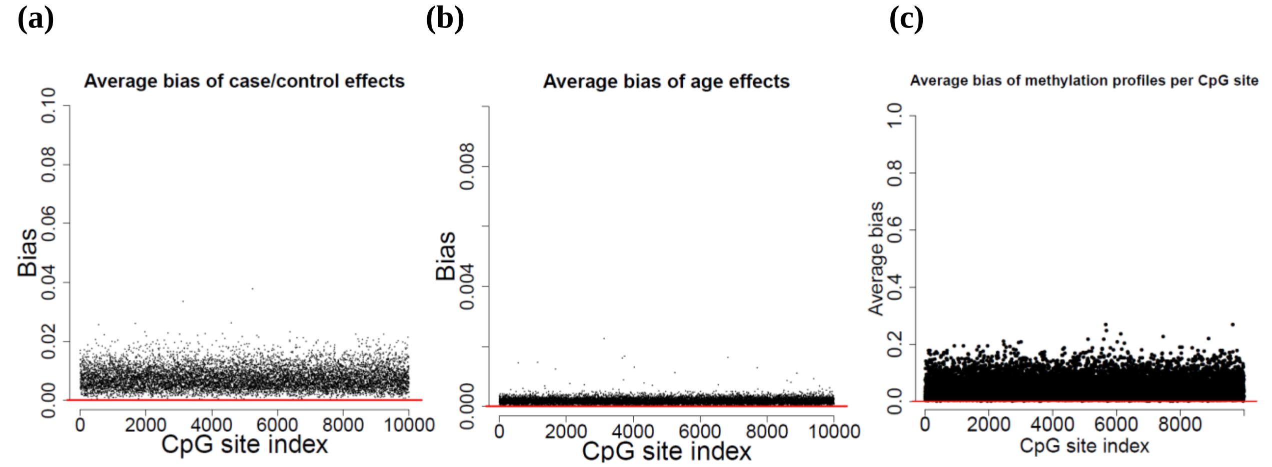

In the "true null" case with K=3 and n=180,the average biasplots for (a) the case/control effects and (b) the age effects. In (a), the y axis is the $\frac{1}{K}\sum_{k=1}^{K}|\hat{\beta}_{jk1}-\beta_{jk1}|$ and the x axis is the CpG site index j for $j=1,\ldots,m$. In (b), the y axis is the $\frac{1}{K}\sum_{k=1}^{K}|\hat{\beta}_{jk2}-\beta_{jk2}|$ and the x axis is the CpG site index j for $j=1,\ldots,m$. In (c), the y axis is the $\frac{1}{K}\sum_{k=1}^{K}|\hat{\mu}_{jk}-\mu_{jk}|$ and the x-axis is the CpG site index j for $j=1,\ldots,m$.

Supplementary Figure 29.

(a)

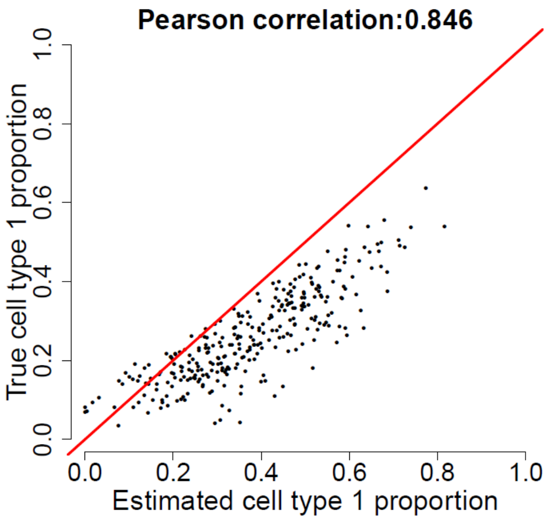

(b)

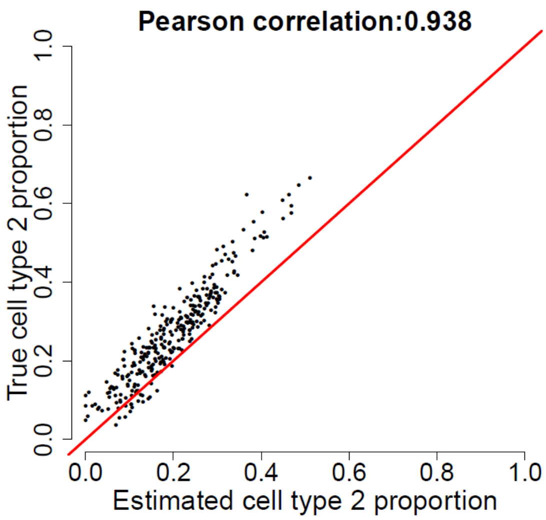

(c)

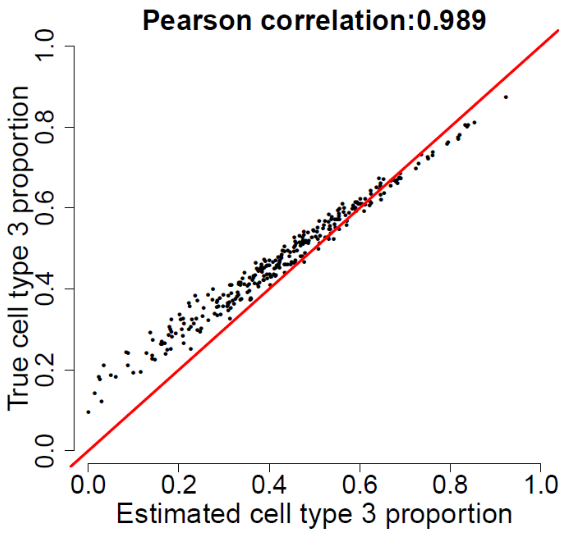

(d)

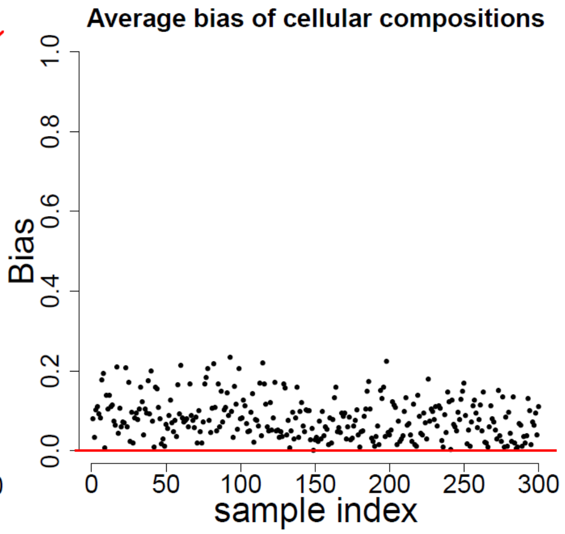

In the “true null” case with K=3 and n=300, (a-c) the scatter plots for the estimated cell proportions by HIRE and the underlying truth for (a) cell type 1; (b) cell type 2; and (c) cell type 3. (d) The average biasplot for the cellular compositions, where the y axis is $\frac{1}{K}\sum_{k=1}^{K} |\widehat{p}_{ki} - p_{ki}|$ and the x axis is the sample index i for $i = 1, \ldots, n$.

Supplementary Figure 30.

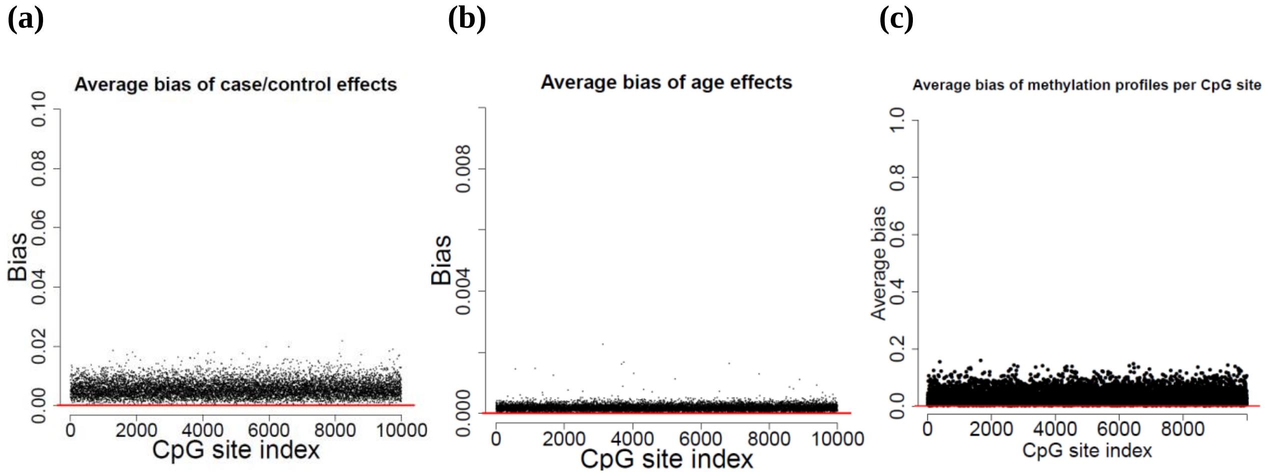

In the "true null" case with K=3 and n=300,the average biasplots for (a) the case/control effects and (b) the age effects. In (a), the y axis is the $\frac{1}{K}\sum_{k=1}^{K}|\hat{\beta}_{jk1} - \beta_{jk1}|$ and the x axis is the CpG site index j for $j = 1, \dots, m$. In (b), the y axis is the $\frac{1}{K}\sum_{k=1}^{K}|\hat{\beta}_{jk2} - \beta_{jk2}|$ and the x axis is the CpG site index j for $j = 1, \dots, m$. In (c), the y axis is the $\frac{1}{K}\sum_{k=1}^{K}|\hat{\mu}_{jk} - \mu_{jk}|$ and the x-axis is the CpG site index j for $j = 1, \dots, m$.

Supplementary Figure 31.

(a)

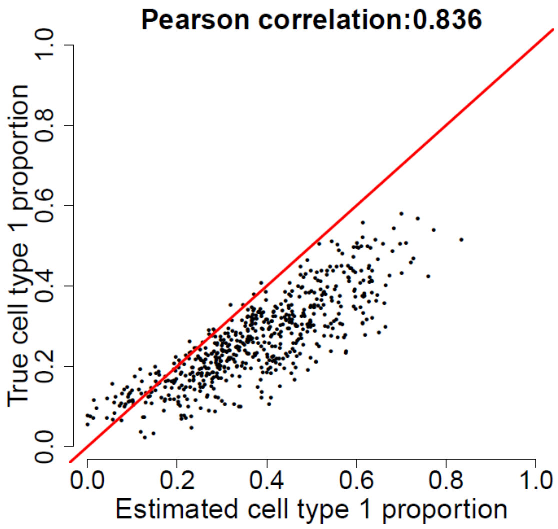

(b)

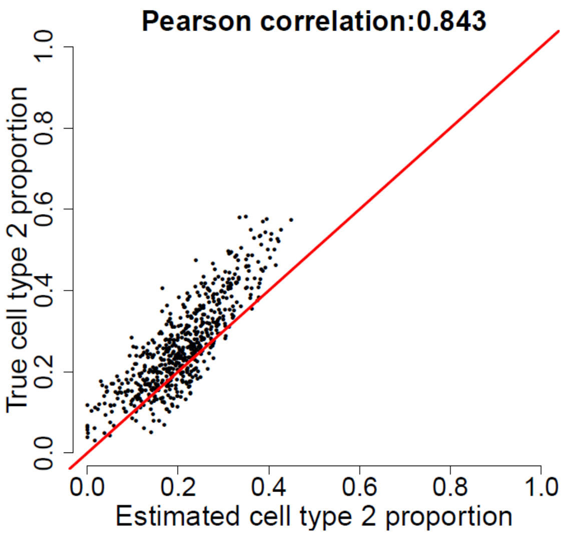

(c)

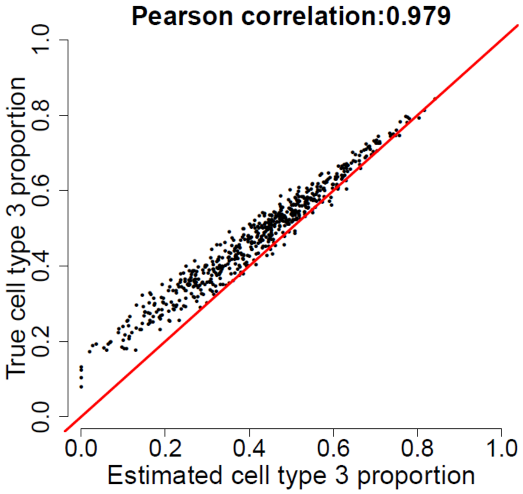

(d)

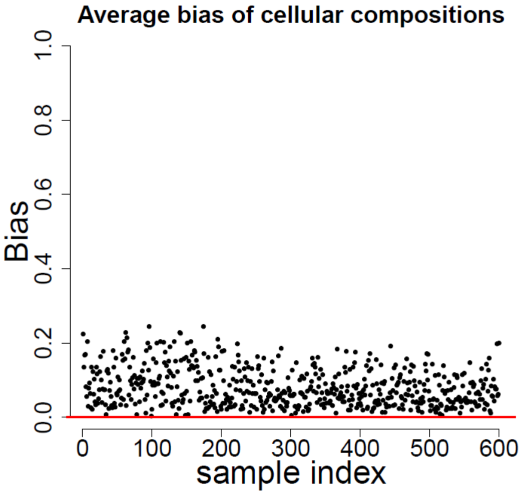

In the "true null" case with K=3 and n=300, (a-c) the scatter plots for the estimated cell proportions by HIRE and the underlying truth for (a) cell type 1; (b) cell type 2; and (c) cell type 3. (d) The average biasplot for the cellular compositions, where the y axis is $\frac{1}{K}\sum_{k=1}^{K}|\widehat{p}_{ki} - p_{ki}|$ and the x axis is the sample index i for $i = 1, \dots, n$.

Supplementary Figure 32.

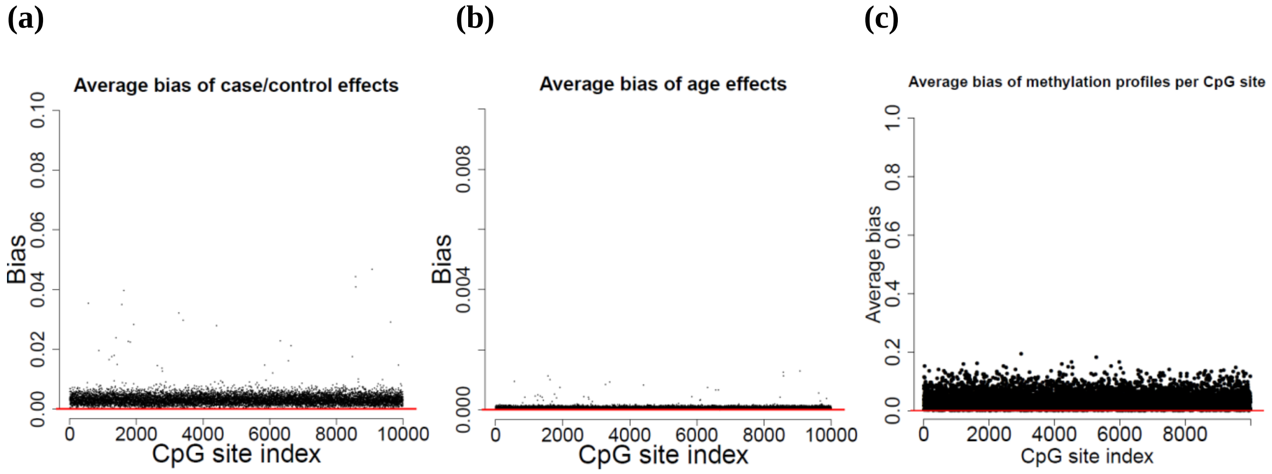

In the "true null" case with K=3 and n=600,the average biasplots for (a) the case/control effects and (b) the age effects.In (a), the y axis is the $\frac{1}{K}\sum_{k=1}^{K}|\hat{\beta}_{jk1}-\beta_{jk1}|$ and the x axis is the CpG site index j for $j=1,\ldots,m$. In (b), the y axis is the $\frac{1}{K}\sum_{k=1}^{K}|\hat{\beta}_{jk2}-\beta_{jk2}|$ and the x axis is the CpG site index j for $j=1,\ldots,m$. In (c), the y axis is the $\frac{1}{K}\sum_{k=1}^{K}|\hat{\mu}_{jk}-\mu_{jk}|$ and the x-axis is the CpG site index j for $j=1,\ldots,m$.

Supplementary Figure 33.

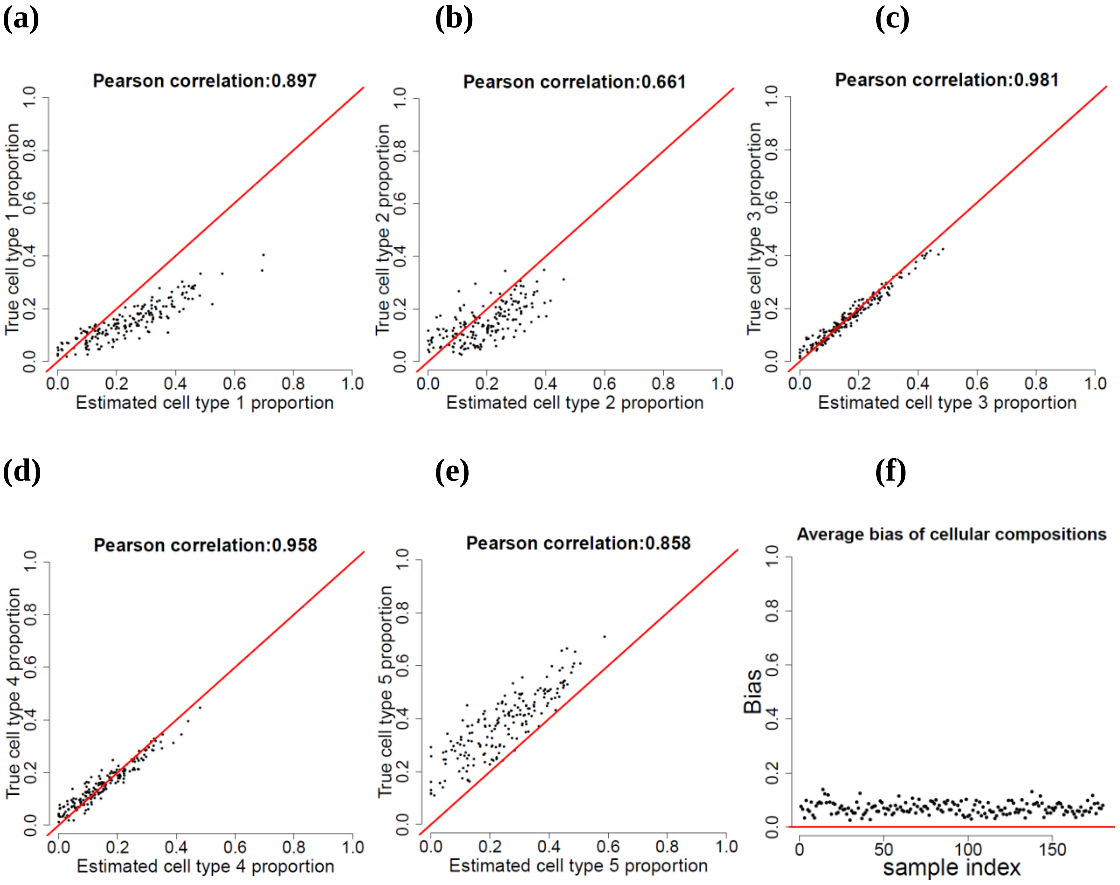

In the “true null” case with K=5 and n=180, (a-e) the scatter plots for the estimated cell proportions by HIRE and the underlying truth for (a) cell type 1; (b) cell type 2; (c) cell type 3; (d) cell type 4; and (e) cell type 5. (f) The average biasplot for the cellular compositions, where the y axis is $\frac{1}{K}\sum_{k=1}^{K} |\widehat{p}_{ki} - p_{ki}|$ and the x axis is the sample index i for $i = 1, \dots, n$.

Supplementary Figure 34.

(a) (b) (c)

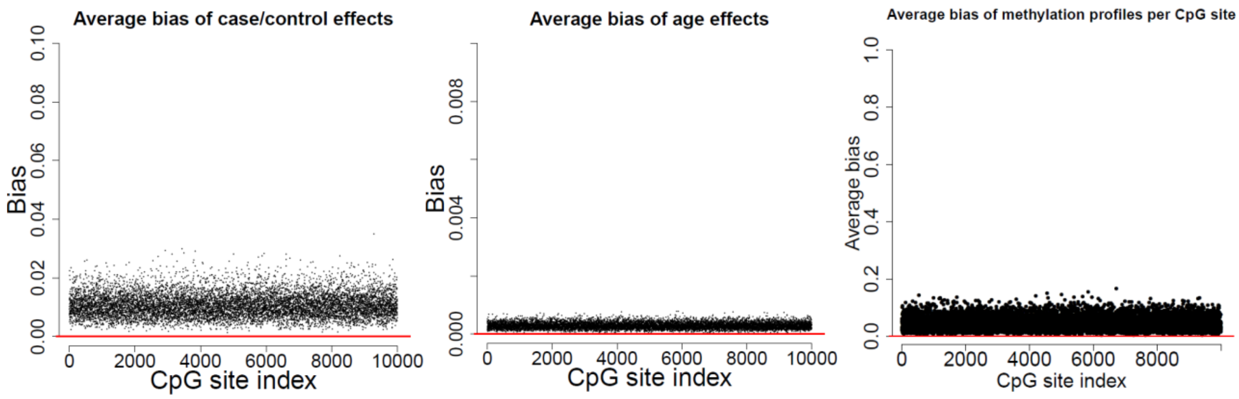

In the "true null" case with K=5 and n=180,the average biasplots for (a) the case/control effects and (b) the age effects. In (a), the y axis is the $\frac{1}{K}\sum_{k=1}^{K}|\hat{\beta}_{jk1} - \beta_{jk1}|$ and the x axis is the CpG site index j for $j = 1, \dots, m$. In (b), the y axis is the $\frac{1}{K}\sum_{k=1}^{K}|\hat{\beta}_{jk2} - \beta_{jk2}|$ and the x axis is the CpG site index j for $j = 1, \dots, m$. In (c), the y axis is the $\frac{1}{K}\sum_{k=1}^{K}|\hat{\mu}_{jk} - \mu_{jk}|$ and the x-axis is the CpG site index j for $j = 1, \dots, m$.

Supplementary Figure 35.

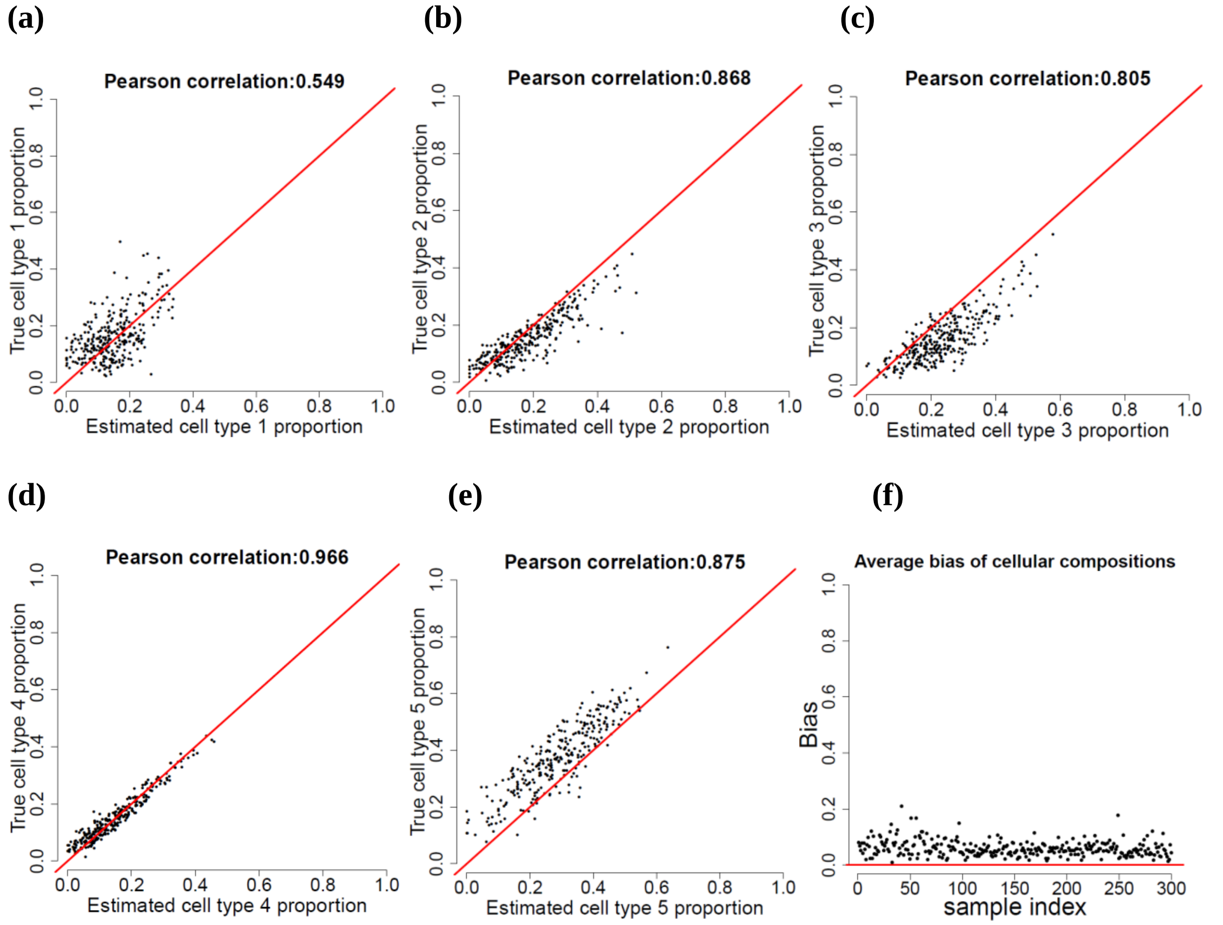

In the "true null" case with K=5 and n=300, (a-e) the scatter plots for the estimated cell proportions by HIRE and the underlying truth for (a) cell type 1; (b) cell type 2; (c) cell type 3; (d) cell type 4; and (e) cell type 5. (f) The average biasplot for the cellular compositions, where the y axis is $\frac{1}{K}\sum_{k=1}^{K}|\widehat{p}_{ki} - p_{ki}|$ and the x axis is the sample index i for $i = 1, \ldots, n$.

Supplementary Figure 36.

(a) **(b)** **(c)**

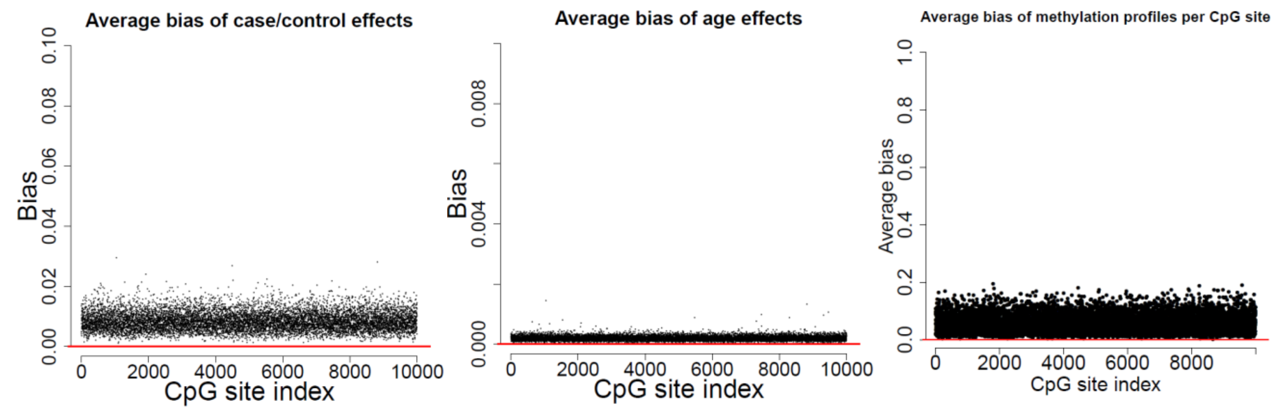

In the "true null" case with K=5 and n=300,the average biasplots for (a) the case/control effects and (b) the age effects. In (a), the y axis is the $\frac{1}{K}\sum_{k=1}^{K}|\hat{\beta}_{jk1}-\beta_{jk1}|$ and the x axis is the CpG site index j for $j=1,\ldots,m$. In (b), the y axis is the $\frac{1}{K}\sum_{k=1}^{K}|\hat{\beta}_{jk2}-\beta_{jk2}|$ and the x axis is the CpG site index j for $j=1,\ldots,m$. In (c), the y axis is the $\frac{1}{K}\sum_{k=1}^{K}|\hat{\mu}_{jk}-\mu_{jk}|$ and the x-axis is the CpG site index j for $j=1,\ldots,m$.

Supplementary Figure 37.

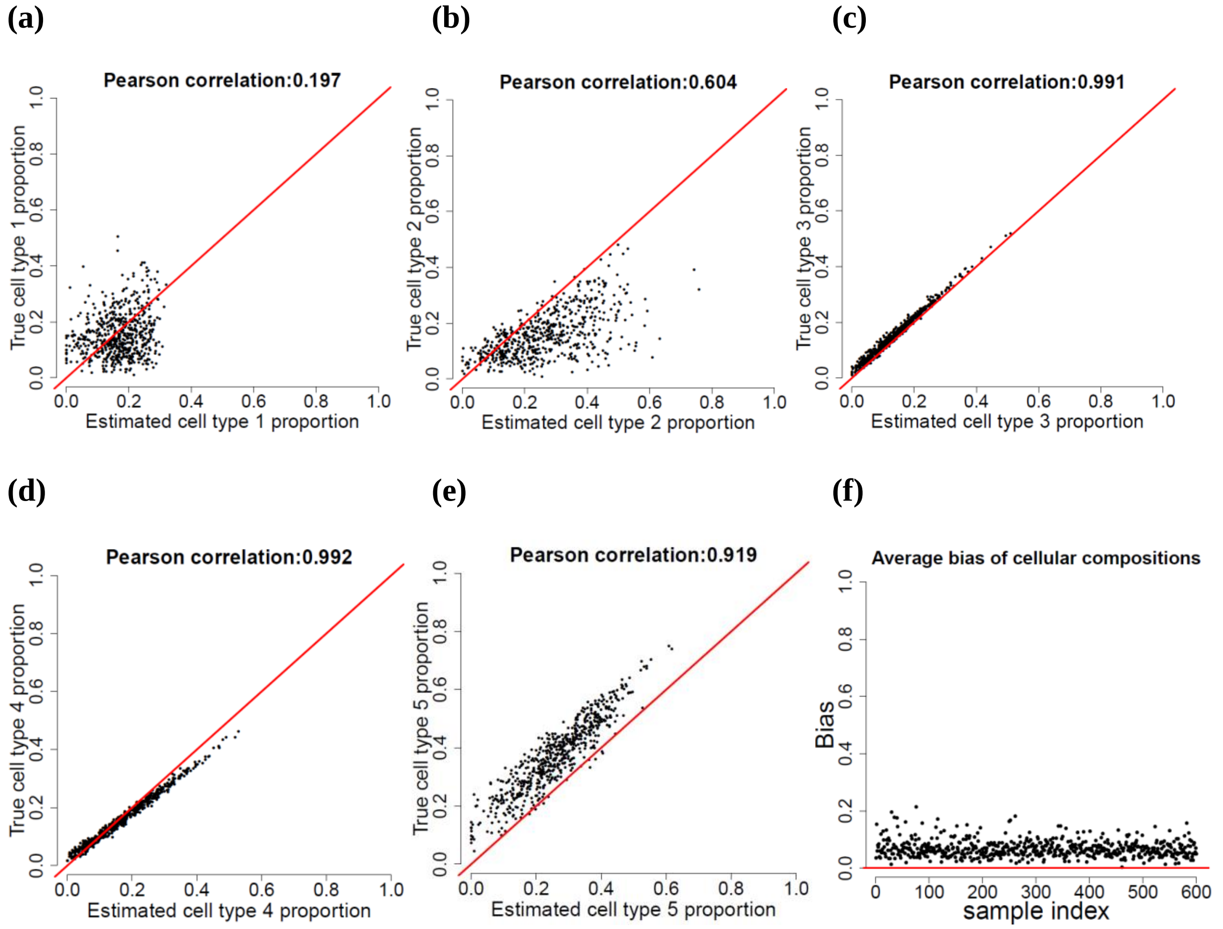

In the “true null” case with K=5 and n=600, (a-e) the scatter plots for the estimated cell proportions by HIRE and the underlying truth for (a) cell type 1; (b) cell type 2; (c) cell type 3; (d) cell type 4; and (e) cell type 5. (f) The average biasplot for the cellular compositions, where the y axis is $\frac{1}{K}\sum_{k=1}^{K}|\widehat{p}_{ki} - p_{ki}|$ and the x axis is the sample index i for $i = 1, \ldots, n$.

Supplementary Figure 38.

(a) (b) (c)

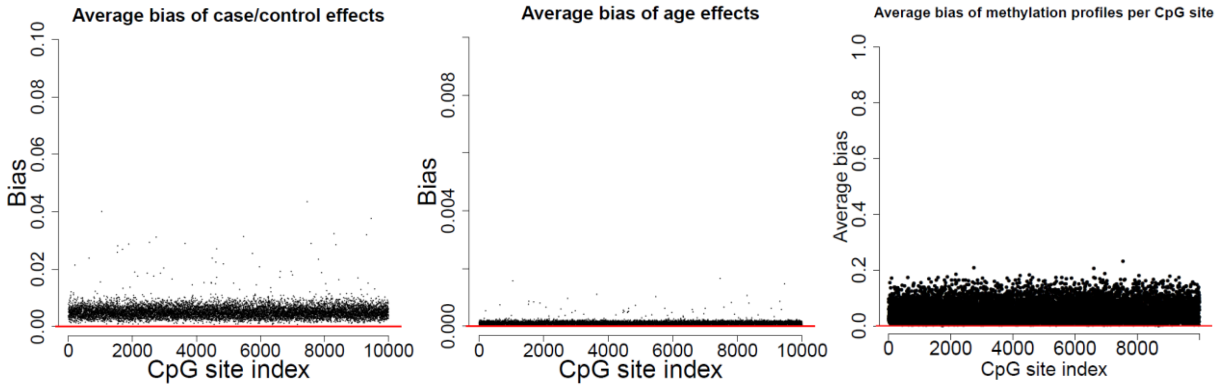

In the "true null" case with K=5 and n=600,the average biasplots for (a) the case/control effects and (b) the age effects. In (a), the y axis is the $\frac{1}{K}\sum_{k=1}^{K}|\hat{\beta}_{jk1} - \beta_{jk1}|$ and the x axis is the CpG site index j for $j = 1, \ldots, m$. In (b), the y axis is the $\frac{1}{K}\sum_{k=1}^{K}|\hat{\beta}_{jk2} - \beta_{jk2}|$ and the x axis is the CpG site index j for $j = 1, \ldots, m$. In (c), the y axis is the $\frac{1}{K}\sum_{k=1}^{K}|\hat{\mu}_{jk} - \mu_{jk}|$ and the x-axis is the CpG site index j for $j = 1, \ldots, m$.

Supplementary Figure 39.

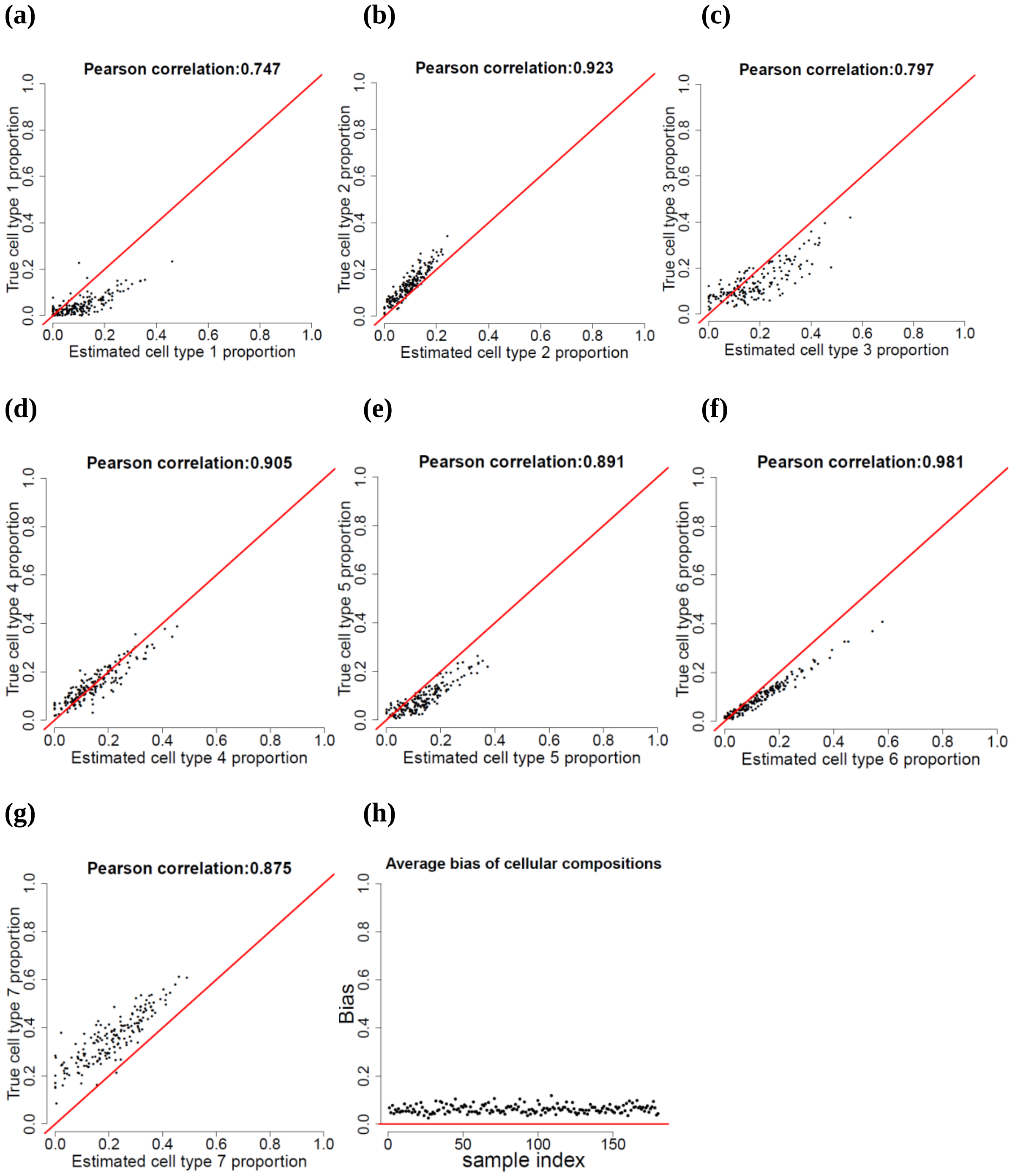

In the "true null" case with K=7 and n=180, (a-g) the scatter plots for the estimated cell proportions by HIRE and the underlying truth for (a) cell type 1; (b) cell type 2; (c) cell type 3; (d) cell type 4; (e) cell type 5; (f) cell type 6; and (g) cell type 7. (h) The average biasplot for the cellular compositions, where the y axis is $\frac{1}{K}\sum_{k=1}^{K} |\widehat{p}_{ki} - p_{ki}|$ and the x axis is the sample index i for $i = 1, \ldots, n$.

Supplementary Figure 40.

(a) **(b)** **(c)**

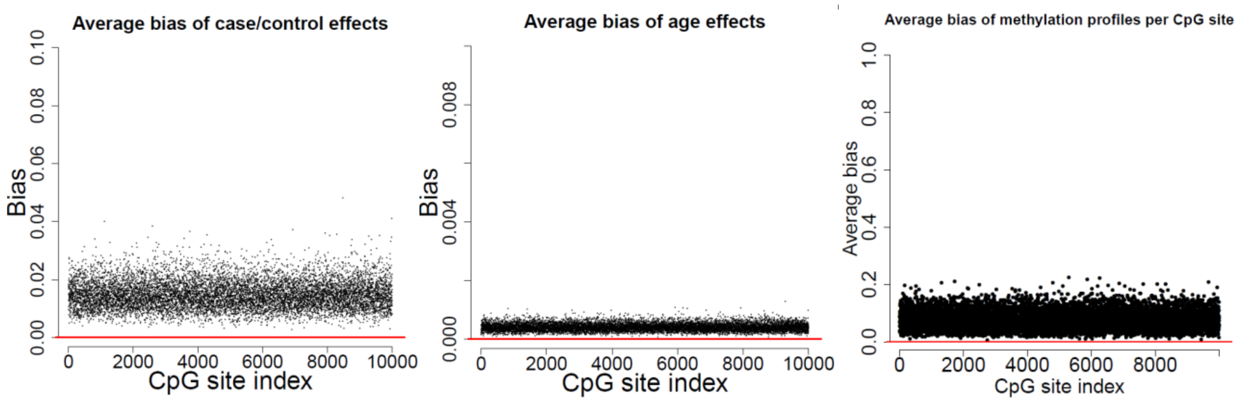

In the "true null" case with K=7 and n=180,the average biasplots for (a) the case/control effects and (b) the age effects. In (a), the y axis is the $\frac{1}{K}\sum_{k=1}^{K}|\hat{\beta}_{jk1}-\beta_{jk1}|$ and the x axis is the CpG site index j for $j=1,\dots,m$. In (b), the y axis is the $\frac{1}{K}\sum_{k=1}^{K}|\hat{\beta}_{jk2}-\beta_{jk2}|$ and the x axis is the CpG site index j for $j=1,\dots,m$. In (c), the y axis is the $\frac{1}{K}\sum_{k=1}^{K}|\hat{\mu}_{jk}-\mu_{jk}|$ and the x-axis is the CpG site index j for $j=1,\dots,m$.

Supplementary Figure 41.

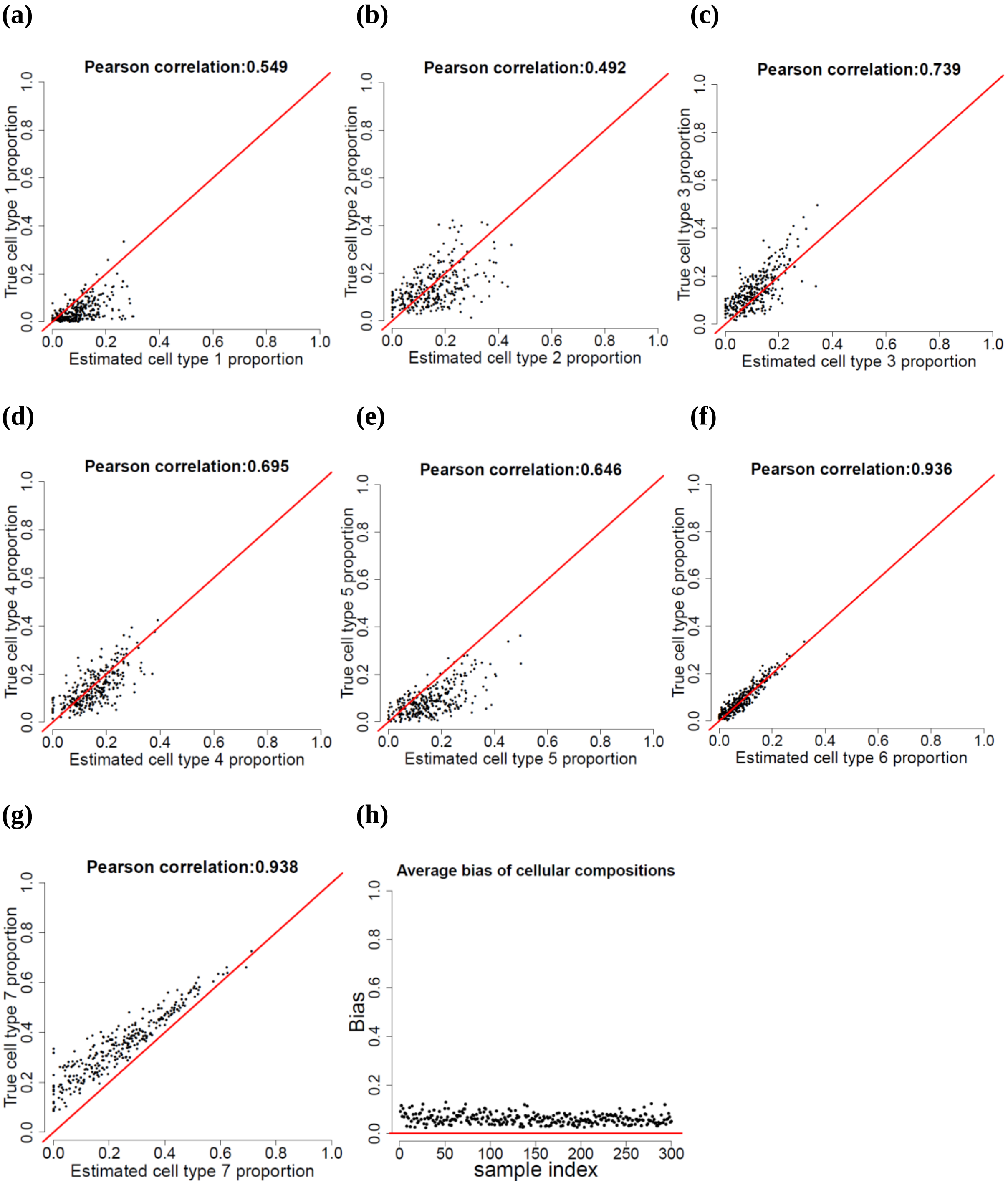

In the "true null" case with K=7 and n=300, (a-g) the scatter plots for the estimated cell proportions by HIRE and the underlying truth for (a) cell type 1; (b) cell type 2; (c) cell type 3; (d) cell type 4; (e) cell type 5; (f) cell type 6; and (g) cell type 7. (h) The average biasplot for the cellular compositions, where the y axis is $\frac{1}{K}\sum_{k=1}^{K}|\widehat{p}_{ki} - p_{ki}|$ and the x axis is the sample index i for $i = 1, \ldots, n$.

Supplementary Figure 42.

(a) **(b)** **(c)**

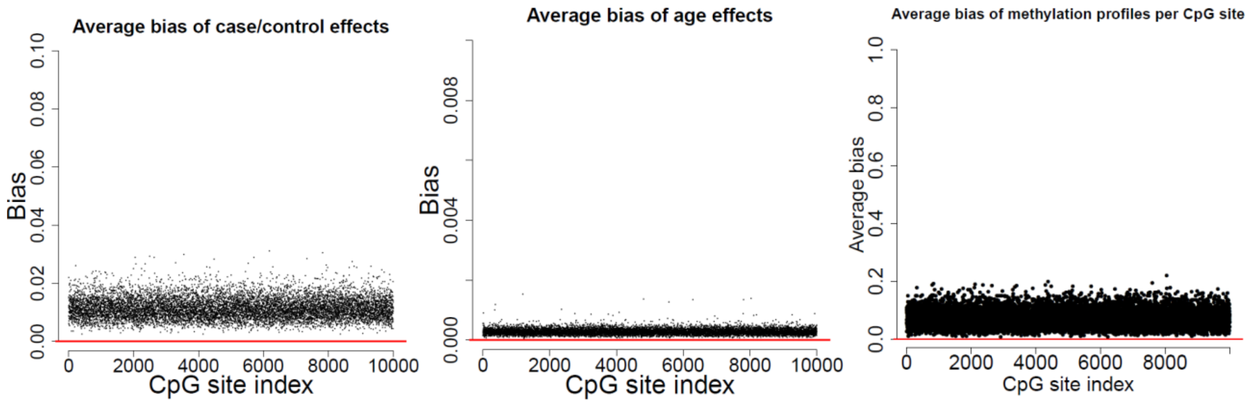

In the "true null" case with K=7 and n=300,the average biasplots for (a) the case/control effects and (b) the age effects. In (a), the y axis is the $\frac{1}{K}\sum_{k=1}^{K}|\hat{\beta}_{jk1} - \beta_{jk1}|$ and the x axis is the CpG site index j for $j = 1, \ldots, m$. In (b), the y axis is the $\frac{1}{K}\sum_{k=1}^{K}|\hat{\beta}_{jk2} - \beta_{jk2}|$ and the x axis is the CpG site index j for $j = 1, \ldots, m$. In (c), the y axis is the $\frac{1}{K}\sum_{k=1}^{K}|\hat{\mu}_{jk} - \mu_{jk}|$ and the x-axis is the CpG site index j for $j = 1, \ldots, m$.

Supplementary Figure 43.

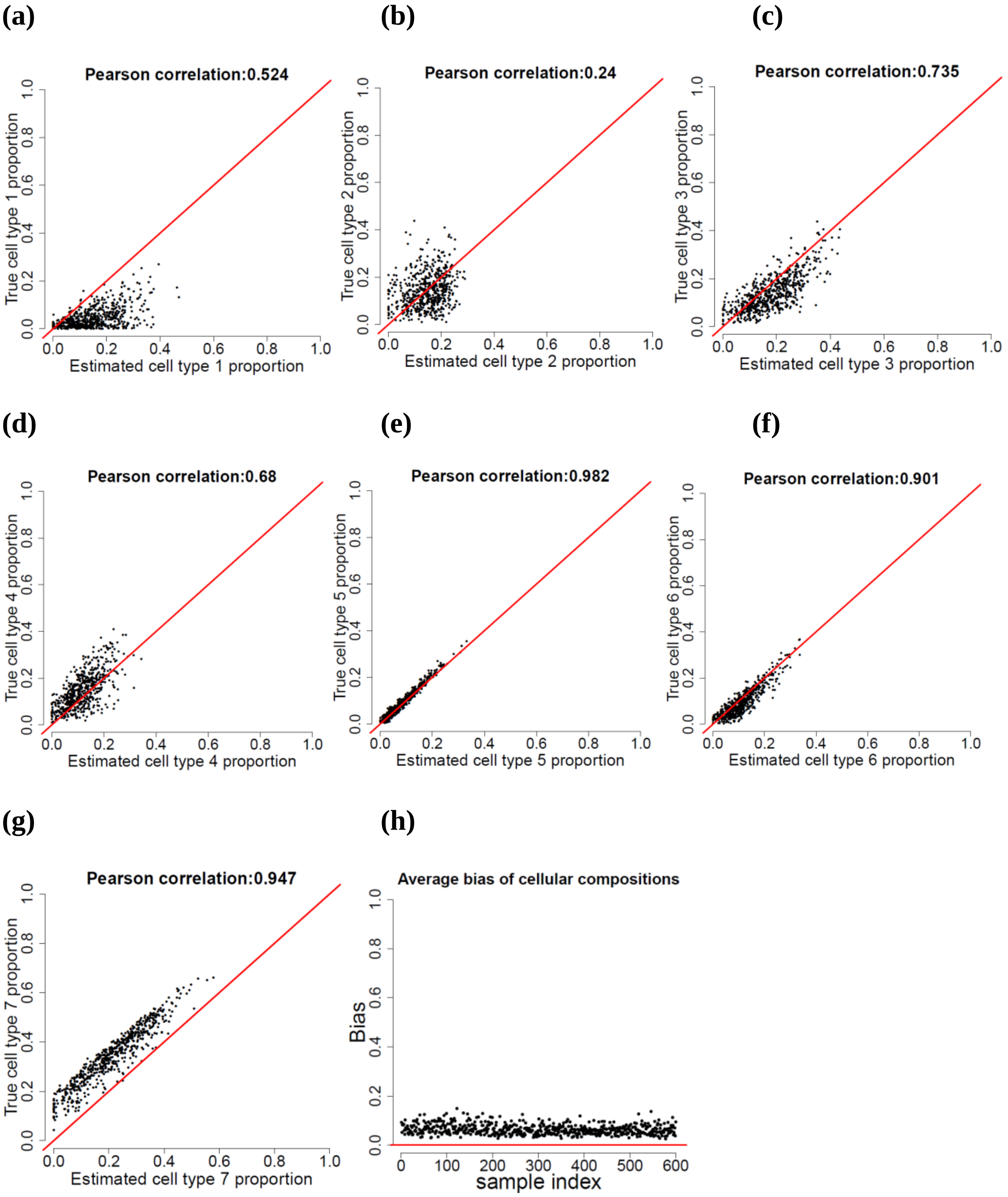

In the "true null" case with K=7 and n=600, (a-g) the scatter plots for the estimated cell proportions by HIRE and the underlying truth for (a) cell type 1; (b) cell type 2; (c) cell type 3; (d) cell type 4; (e) cell type 5; (f) cell type 6; and (g) cell type 7. (h) The average biasplot for the cellular compositions, where the y axis is $\frac{1}{K}\sum_{k=1}^{K}|\widehat{p}_{ki}-p_{ki}|$ and the x axis is the sample index i for $i=1,\ldots,n$.

Supplementary Figure 44.

(a) (b) (c)

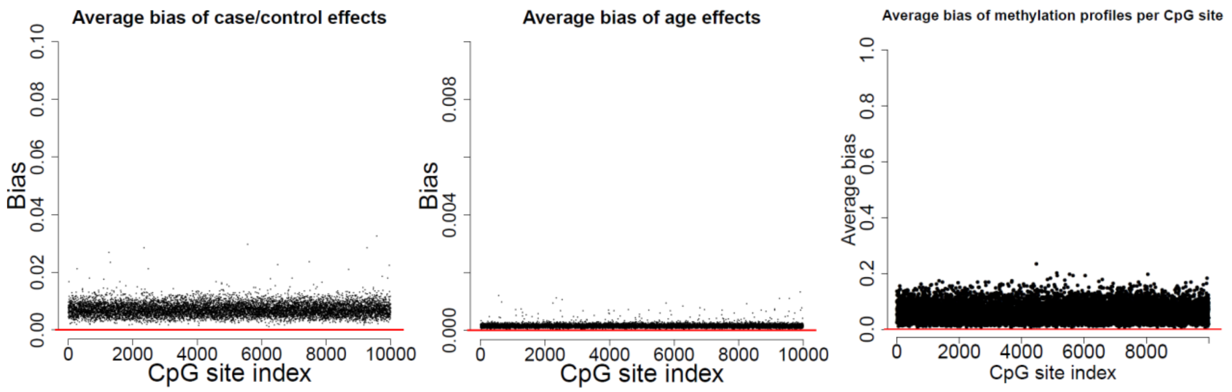

In the "true null" case with K=7 and n=600,the average biasplots for (a) the case/control effects and (b) the age effects. In (a), the y axis is the $\frac{1}{K}\sum_{k=1}^{K}|\hat{\beta}_{jk1}-\beta_{jk1}|$ and the x axis is the CpG site index j for $j=1,\dots,m$. In (b), the y axis is the $\frac{1}{K}\sum_{k=1}^{K}|\hat{\beta}_{jk2}-\beta_{jk2}|$ and the x axis is the CpG site index j for $j=1,\dots,m$. In (c), the y axis is the $\frac{1}{K}\sum_{k=1}^{K}|\hat{\mu}_{jk}-\mu_{jk}|$ and the x-axis is the CpG site index j for $j=1,\dots,m$.

Supplementary Figure 45.

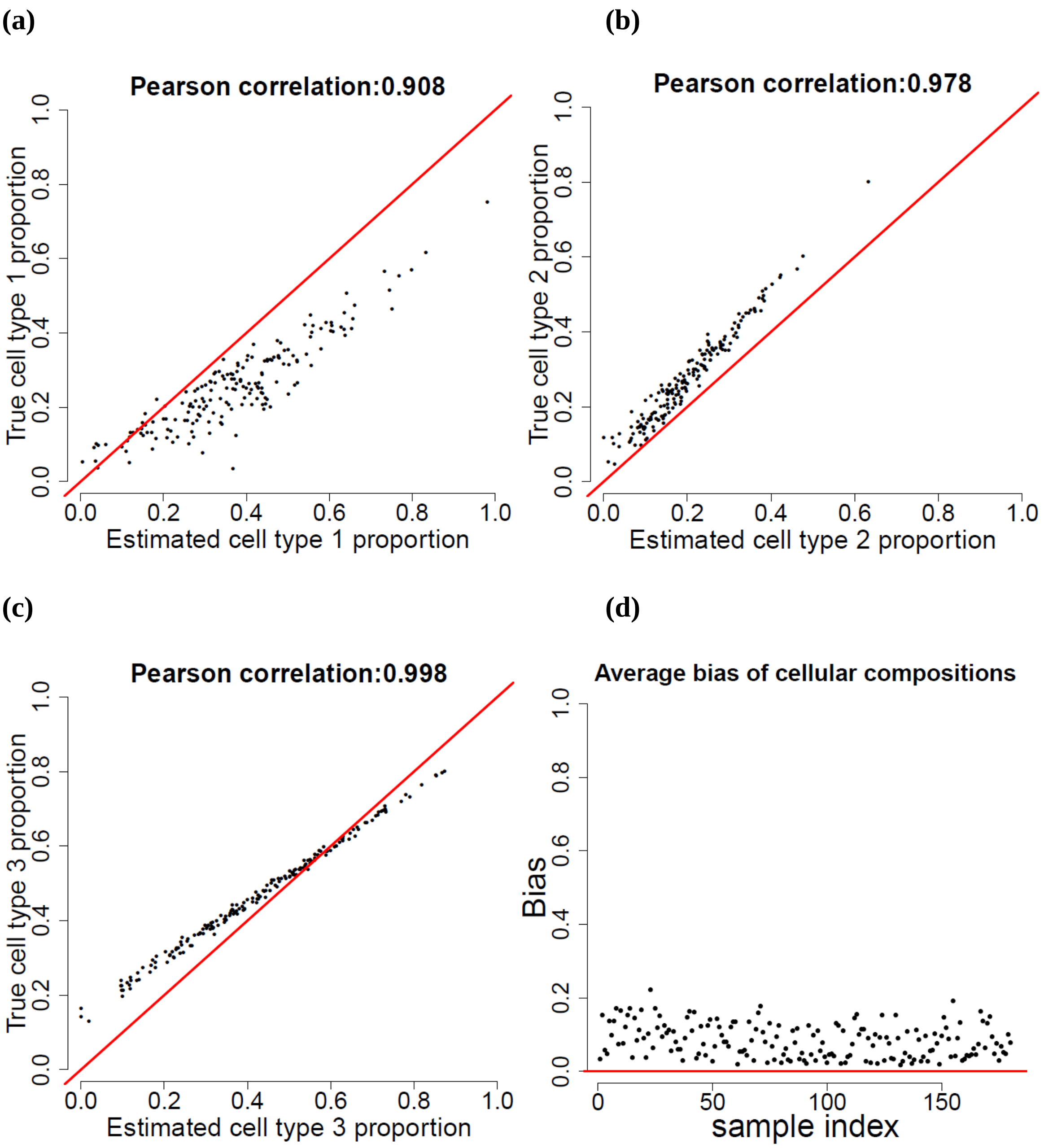

In the “true alternative” case with K=3 and n=180, (a-c) the scatter plots for the estimated cell proportions by HIRE and the underlying truth for (a) cell type 1; (b) cell type 2; and (c) cell type 3. (d) The average biasplot for the cellular compositions, where the y axis is $\frac{1}{K}\sum_{k=1}^{K}|\widehat{p}_{ki} - p_{ki}|$ and the x axis is the sample index i for $i = 1, \dots, n$.

Supplementary Figure 46.

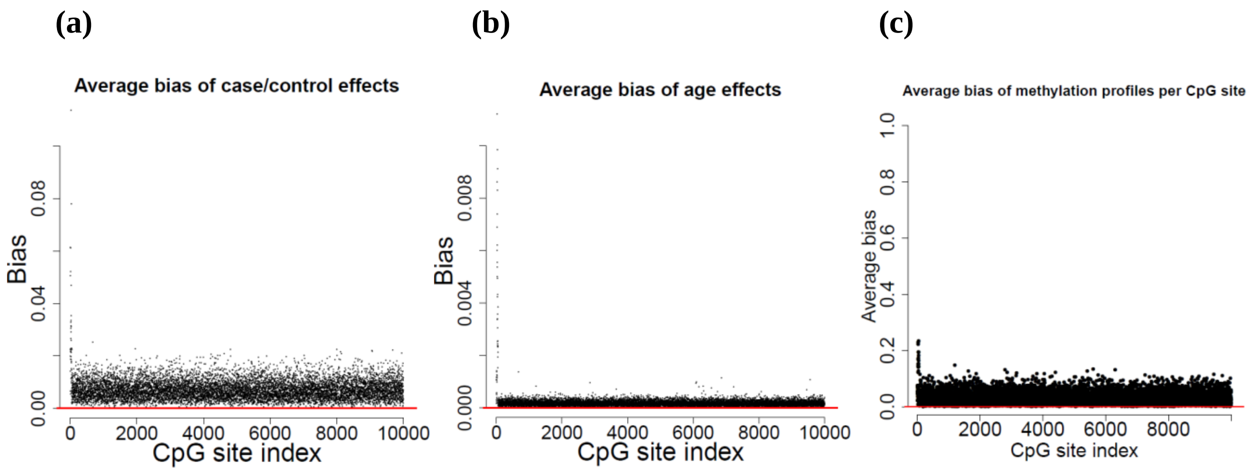

In the "true alternative" case with K=3 and n=180,the average biasplots for (a) the case/control effects and (b) the age effects. In (a), the y axis is the $\frac{1}{K}\sum_{k=1}^{K}|\hat{\beta}_{jk1}-\beta_{jk1}|$ and the x axis is the CpG site index j for $j=1,\dots,m$. In (b), the y axis is the $\frac{1}{K}\sum_{k=1}^{K}|\hat{\beta}_{jk2}-\beta_{jk2}|$ and the x axis is the CpG site index j for $j=1,\dots,m$. In (c), the y axis is the $\frac{1}{K}\sum_{k=1}^{K}|\hat{\mu}_{jk}-\mu_{jk}|$ and the x-axis is the CpG site index j for $j=1,\dots,m$.

Supplementary Figure 47.

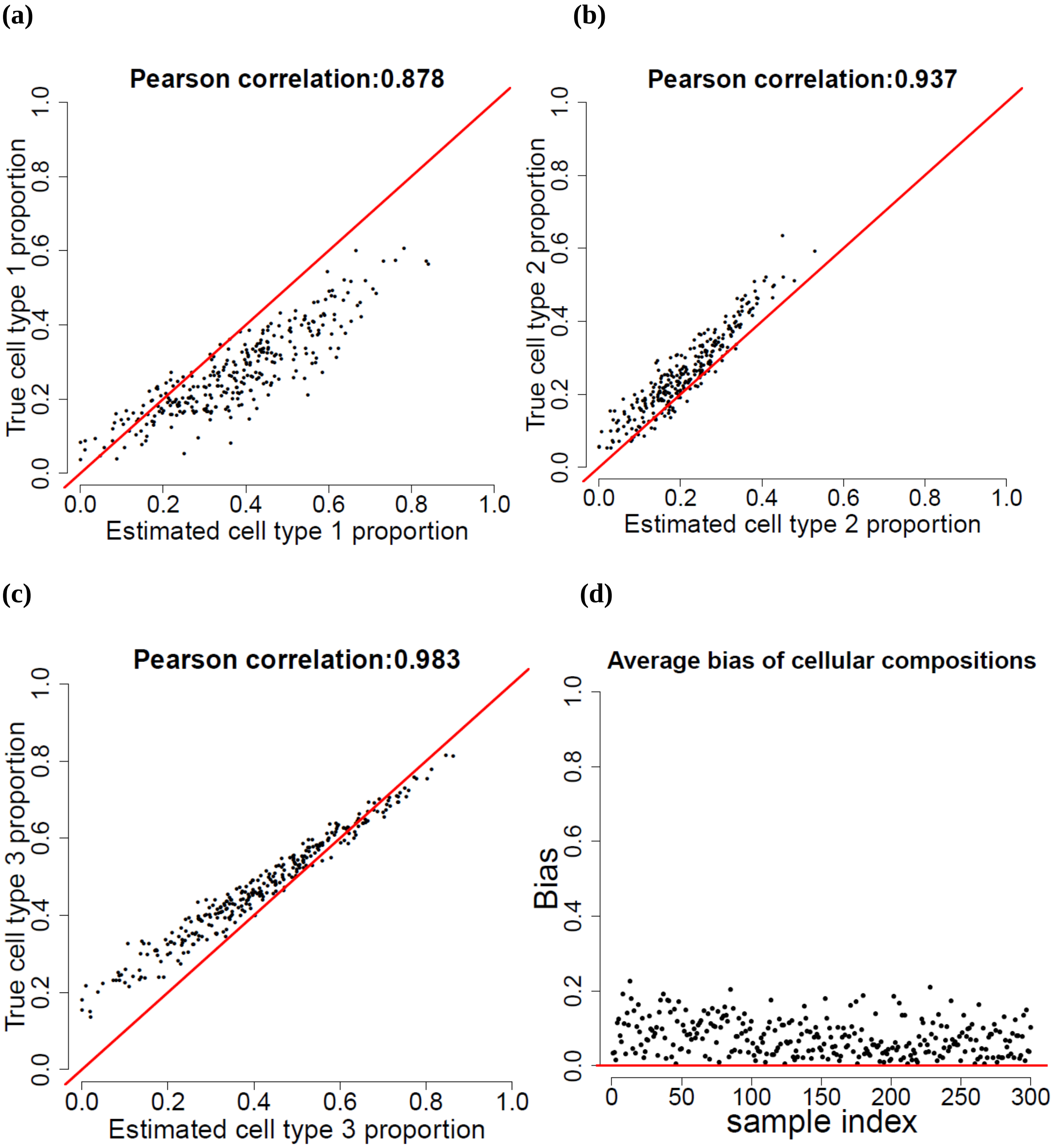

In the “true alternative” case with K=3 and n=300, (a-c) the scatter plots for the estimated cell proportions by HIRE and the underlying truth for (a) cell type 1; (b) cell type 2; and (c) cell type 3. (d) The average biasplot for the cellular compositions, where the y axis is $\frac{1}{K}\sum_{k=1}^{K}|\widehat{p}_{ki} - p_{ki}|$ and the x axis is the sample index i for $i = 1, \ldots, n$.

Supplementary Figure 48.

(a)

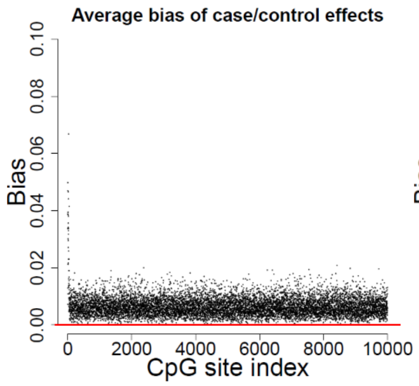

(b)

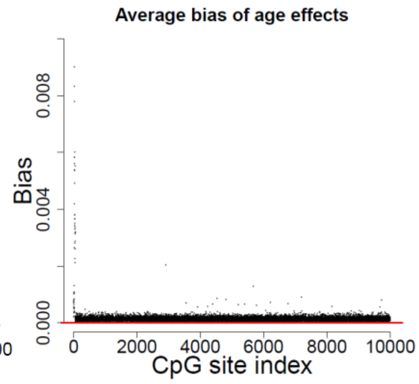

(c)

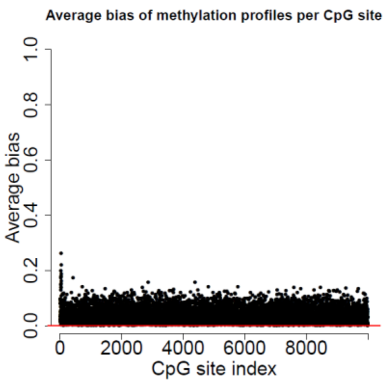

In the "true alternative" case with K=3 and n=300,the average biasplots for (a) the case/control effects and (b) the age effects. In (a), the y axis is the $\frac{1}{K}\sum_{k=1}^{K}|\hat{\beta}_{jk1} - \beta_{jk1}|$ and the x axis is the CpG site index j for $j = 1, \dots, m$. In (b), the y axis is the $\frac{1}{K}\sum_{k=1}^{K}|\hat{\beta}_{jk2} - \beta_{jk2}|$ and the x axis is the CpG site index j for $j = 1, \dots, m$. In (c), the y axis is the $\frac{1}{K}\sum_{k=1}^{K}|\hat{\mu}_{jk} - \mu_{jk}|$ and the x-axis is the CpG site index j for $j = 1, \dots, m$.

Supplementary Figure 49.

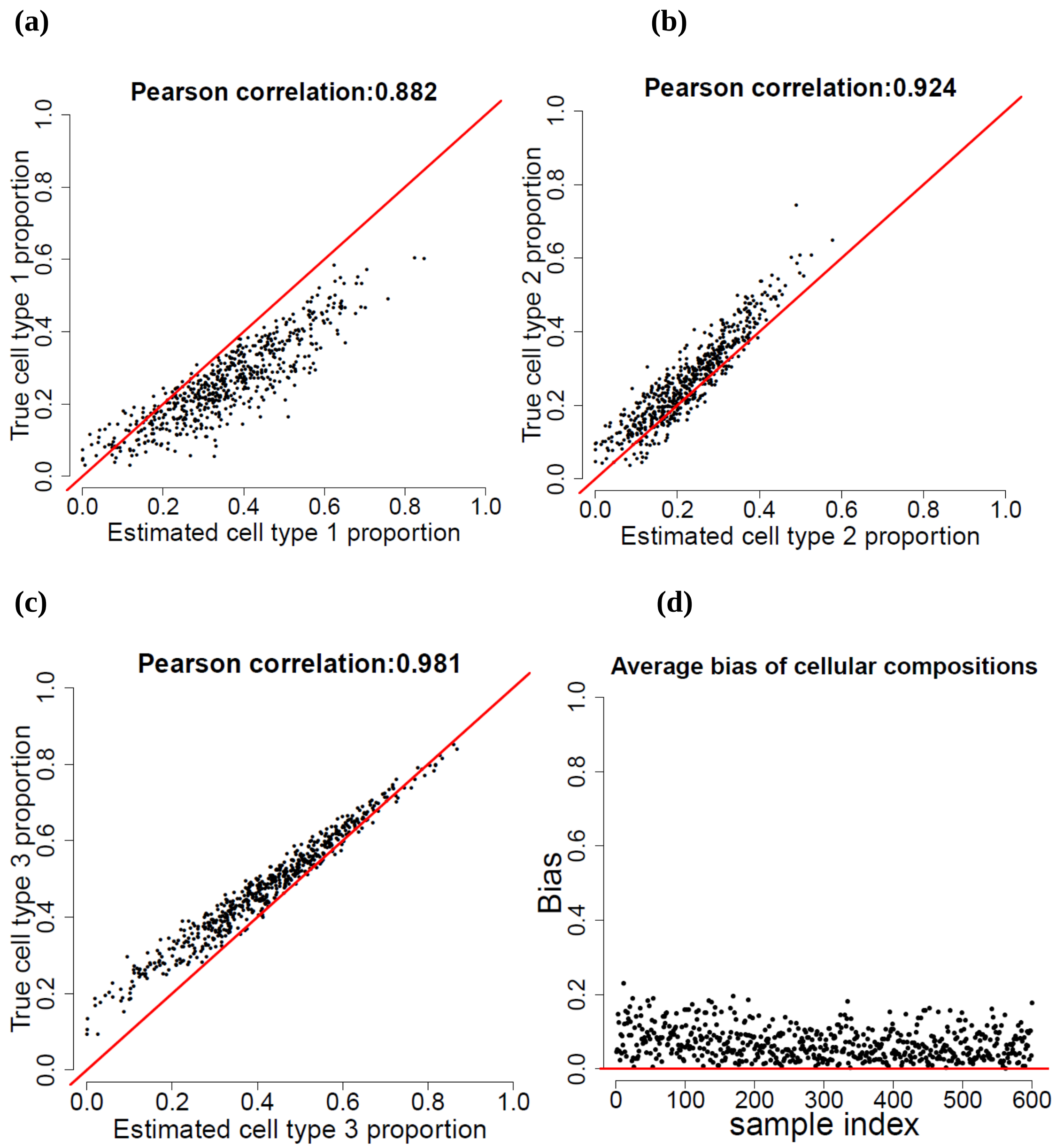

In the "true alternative" case with K=3 and n=600, (a-c) the scatter plots for the estimated cell proportions by HIRE and the underlying truth for (a) cell type 1; (b) cell type 2; and (c) cell type 3. (d) The average biasplot for the cellular compositions, where the y axis is $\frac{1}{K}\sum_{k=1}^{K}|\widehat{p}_{ki} - p_{ki}|$ and the x axis is the sample index i for $i = 1, \ldots, n$.

Supplementary Figure 50.

(a) **(b)** **(c)**

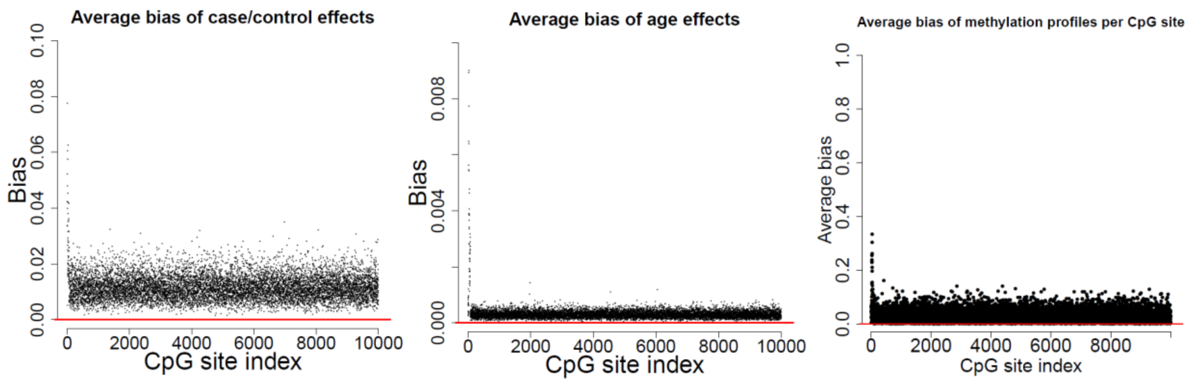

In the “true alternative” case with K=3 and n=600,the average biasplots for (a) the case/control effects and (b) the age effects. In (a), the y axis is the $\frac{1}{K}\sum_{k=1}^{K}|\hat{\beta}_{jk1}-\beta_{jk1}|$ and the x axis is the CpG site index j for $j=1,\ldots,m$. In (b), the y axis is the $\frac{1}{K}\sum_{k=1}^{K}|\hat{\beta}_{jk2}-\beta_{jk2}|$ and the x axis is the CpG site index j for $j=1,\ldots,m$. In (c), the y axis is the $\frac{1}{K}\sum_{k=1}^{K}|\hat{\mu}_{jk}-\mu_{jk}|$ and the x-axis is the CpG site index j for $j=1,\ldots,m$.

Supplementary Figure 51.

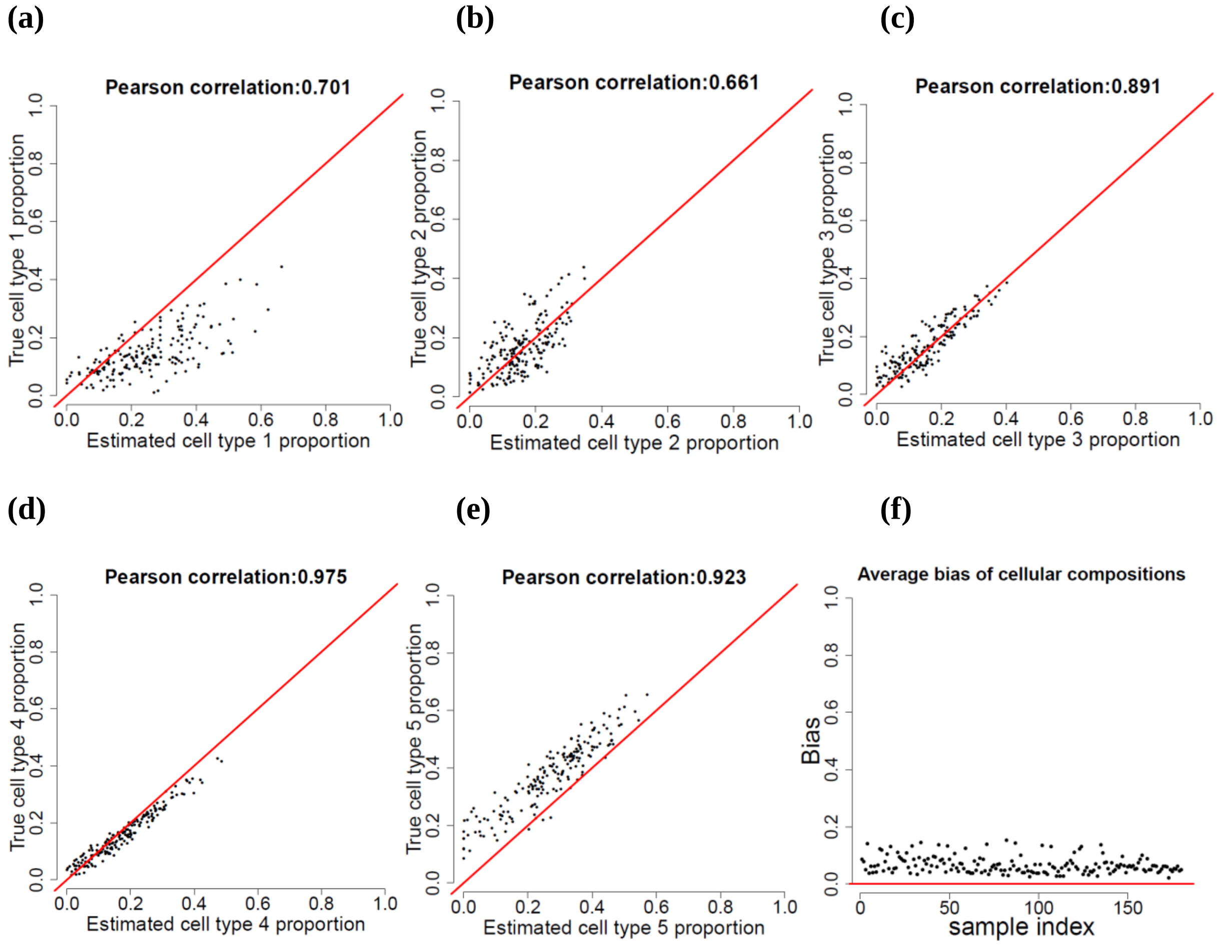

In the "true alternatives" case with K=5 and n=180, (a-e) the scatter plots for the estimated cell proportions by HIRE and the underlying truth for (a) cell type 1; (b) cell type 2; (c) cell type 3; (d) cell type 4; and (e) cell type 5. (f) The average biasplot for the cellular compositions, where the y axis is $\frac{1}{K}\sum_{k=1}^{K} |\widehat{p}_{ki} - p_{ki}|$ and the x axis is the sample index i for $i = 1, \dots, n$.

Supplementary Figure 52.

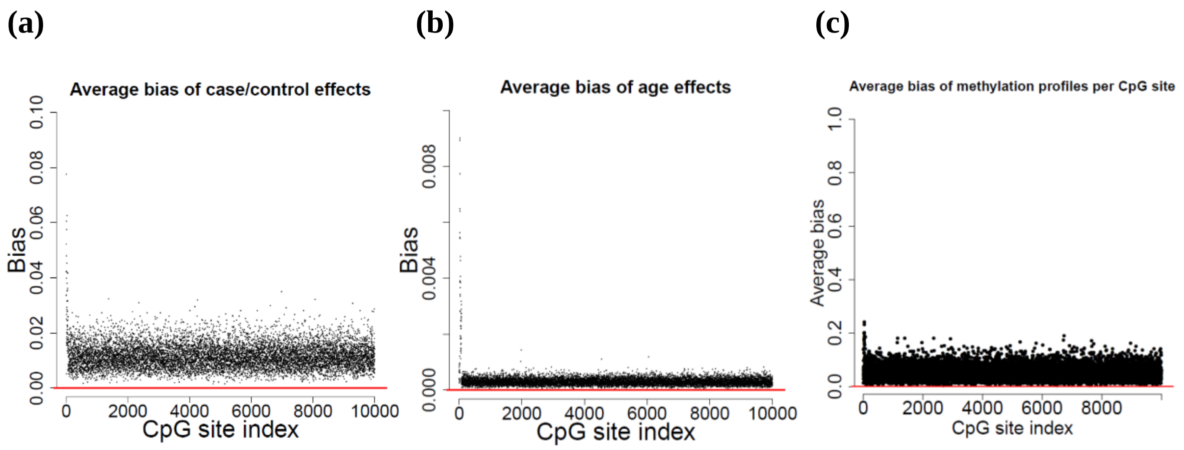

In the "true alternatives" case with K=5 and n=180,the average biasplots for (a) the case/control effects and (b) the age effects.In (a), the y axis is the $\frac{1}{K}\sum_{k=1}^{K}|\hat{\beta}_{jk1}-\beta_{jk1}|$ and the x axis is the CpG site index j for $j=1,\ldots,m$. In (b), the y axis is the $\frac{1}{K}\sum_{k=1}^{K}|\hat{\beta}_{jk2}-\beta_{jk2}|$ and the x axis is the CpG site index j for $j=1,\ldots,m$. In (c), the y axis is the $\frac{1}{K}\sum_{k=1}^{K}|\hat{\mu}_{jk}-\mu_{jk}|$ and the x-axis is the CpG site index j for $j=1,\ldots,m$.

Supplementary Figure 53.

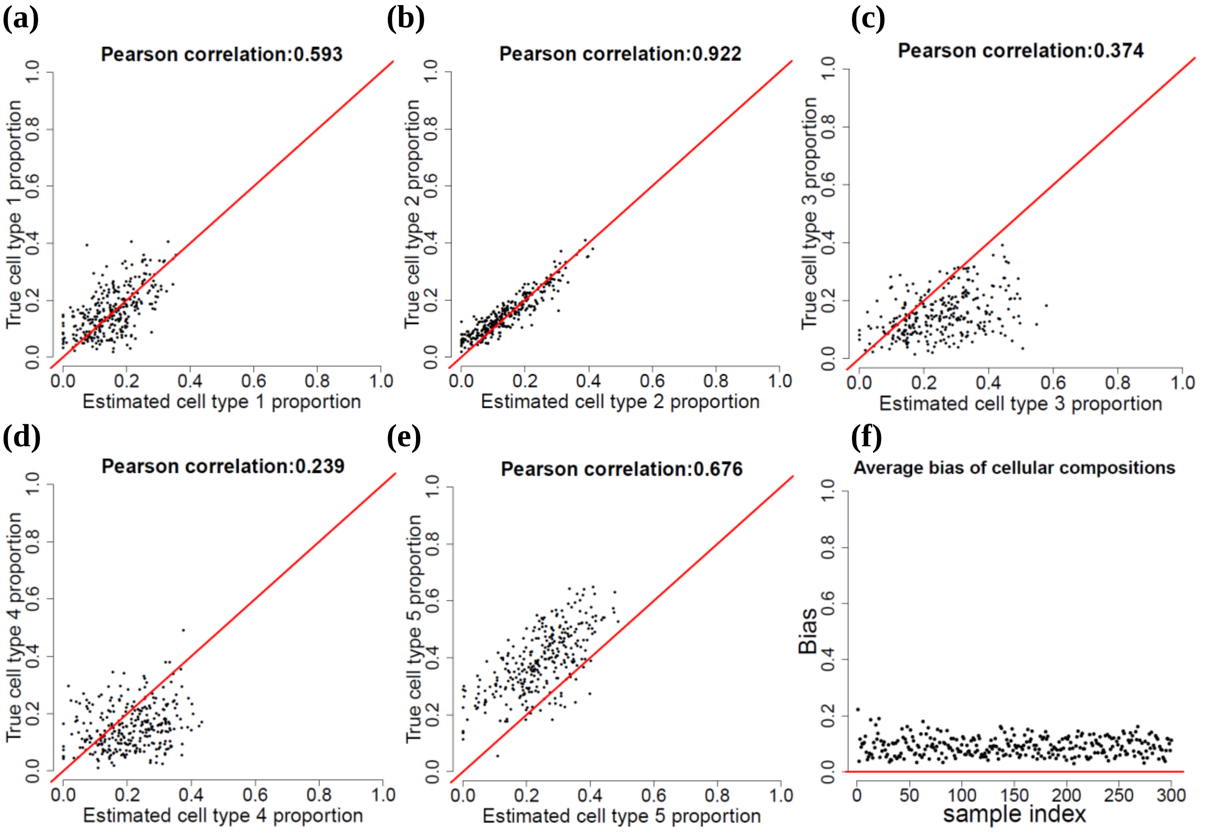

In the “true alternatives” case with K=5 and n=300, (a-e) the scatter plots for the estimated cell proportions by HIRE and the underlying truth for (a) cell type 1; (b) cell type 2; (c) cell type 3; (d) cell type 4; and (e) cell type 5. (f) The average biasplot for the cellular compositions, where the y axis is $\frac{1}{K}\sum_{k=1}^{K}|\widehat{p}_{ki} - p_{ki}|$ and the x axis is the sample index i for $i = 1, \ldots, n$.

Supplementary Figure 54.

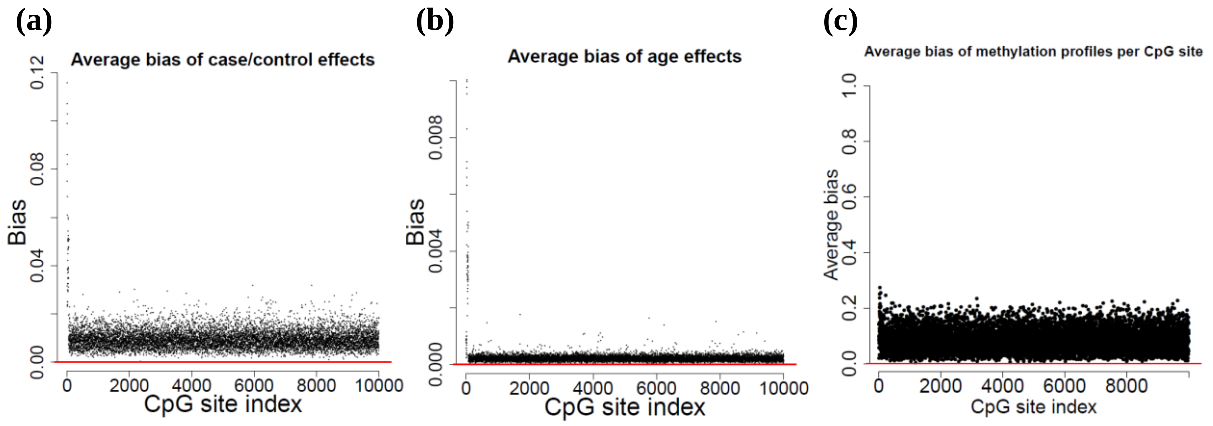

In the “true alternatives” case with K=5 and n=300,the average biasplots for (a) the case/control effects and (b) the age effects. In (a), the y axis is the $\frac{1}{K}\sum_{k=1}^{K}|\hat{\beta}_{jk1} - \beta_{jk1}|$ and the x axis is the CpG site index j for $j = 1, \ldots, m$. In (b), the y axis is the $\frac{1}{K}\sum_{k=1}^{K}|\hat{\beta}_{jk2} - \beta_{jk2}|$ and the x axis is the CpG site index j for $j = 1, \ldots, m$. In (c), the y axis is the $\frac{1}{K}\sum_{k=1}^{K}|\hat{\mu}_{jk} - \mu_{jk}|$ and the x-axis is the CpG site index j for $j = 1, \ldots, m$.

Supplementary Figure 55.

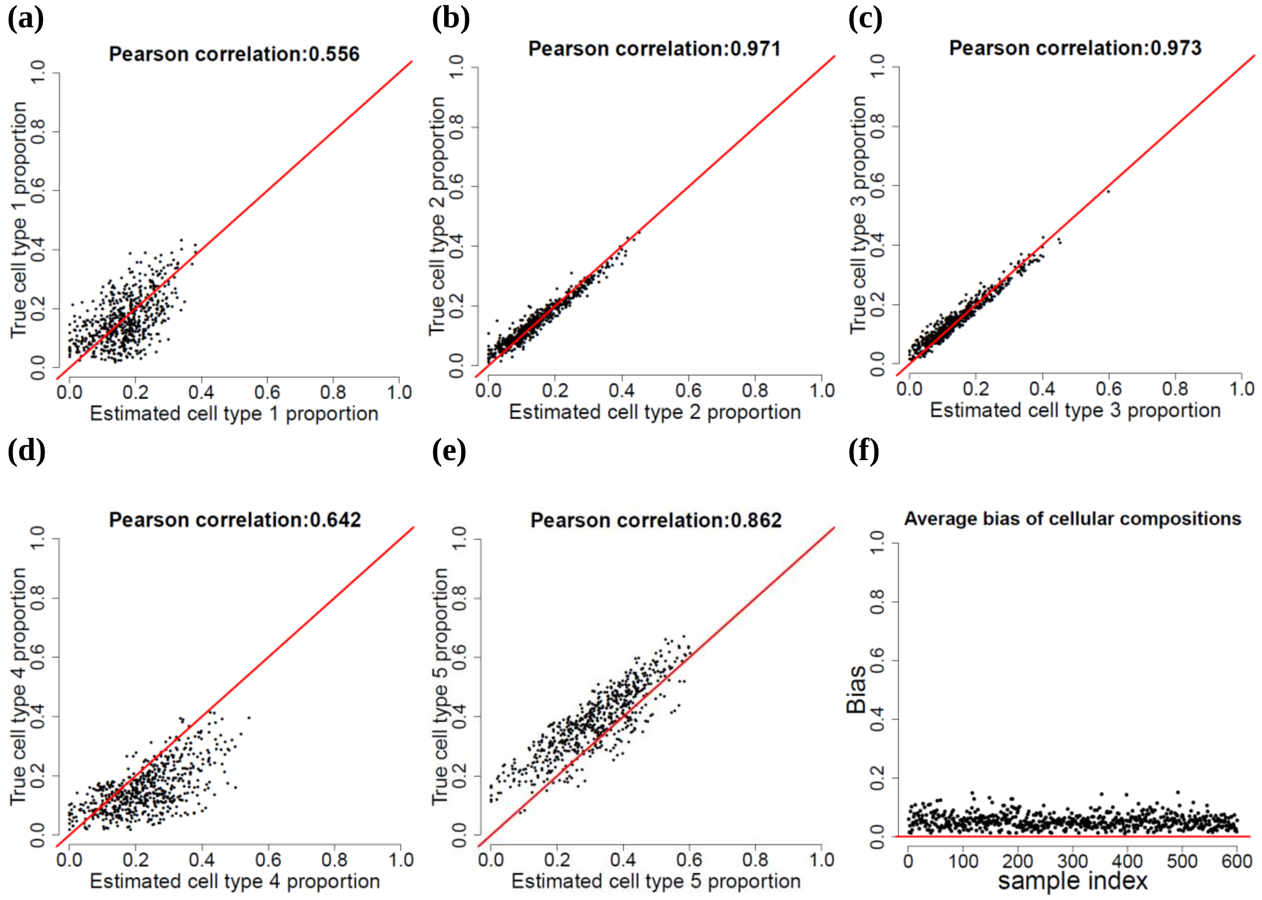

In the “true alternatives” case with K=5 and n=600, (a-e) the scatter plots for the estimated cell proportions by HIRE and the underlying truth for (a) cell type 1; (b) cell type 2; (c) cell type 3; (d) cell type 4; and (e) cell type 5. (f) The average biasplot for the cellular compositions, where the y axis is $\frac{1}{K}\sum_{k=1}^{K}|\widehat{p}_{ki}-p_{ki}|$ and the x axis is the sample index i for $i=1,\ldots,n$.

Supplementary Figure 56.

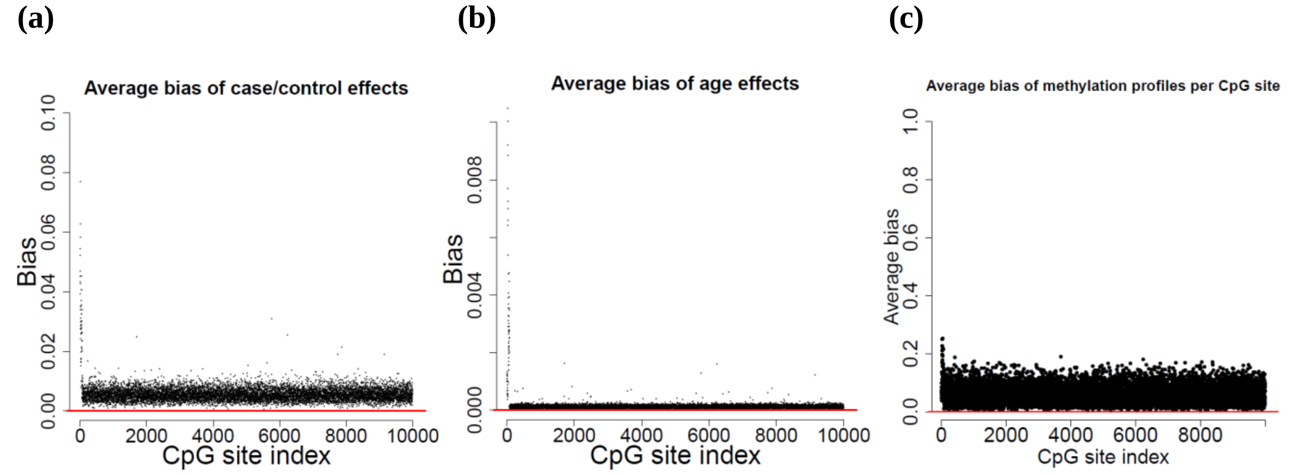

In the "true alternatives" case with K=5 and n=600,the average biasplots for (a) the case/control effects and (b) the age effects. In (a), the y axis is the $\frac{1}{K}\sum_{k=1}^{K}|\hat{\beta}_{jk1}-\beta_{jk1}|$ and the x axis is the CpG site index j for $j=1,\ldots,m$. In (b), the y axis is the $\frac{1}{K}\sum_{k=1}^{K}|\hat{\beta}_{jk2}-\beta_{jk2}|$ and the x axis is the CpG site index j for $j=1,\ldots,m$. In (c), the y axis is the $\frac{1}{K}\sum_{k=1}^{K}|\hat{\mu}_{jk}-\mu_{jk}|$ and the x-axis is the CpG site index j for $j=1,\ldots,m$.

Supplementary Figure 57.

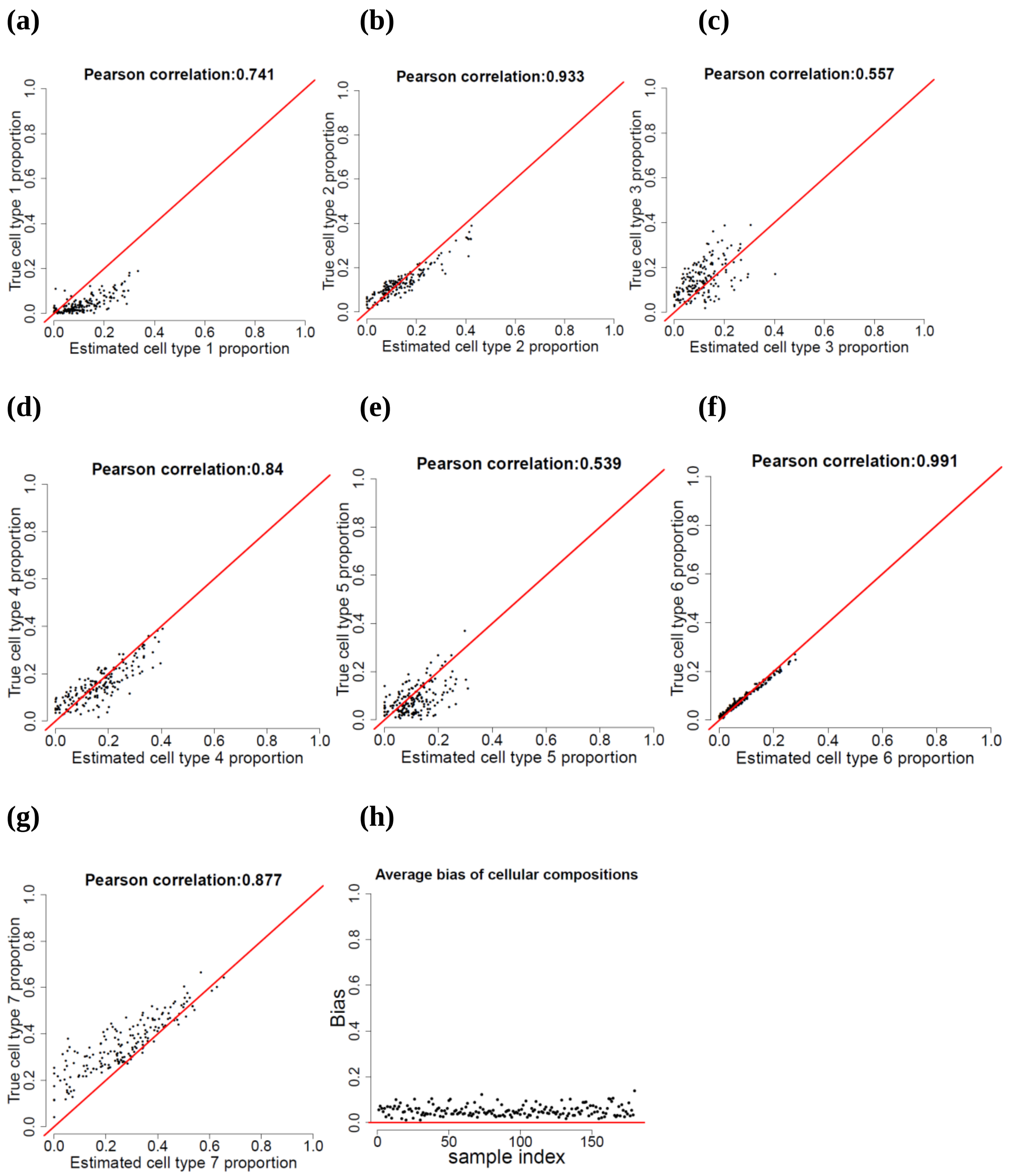

In the "true alternative" case with K=7 and n=180, (a-g) the scatter plots for the estimated cell proportions by HIRE and the underlying truth for (a) cell type 1; (b) cell type 2; (c) cell type 3; (d) cell type 4; (e) cell type 5; (f) cell type 6; and (g) cell type 7. (h) The average biasplot for the cellular compositions, where the y axis is $\frac{1}{K}\sum_{k=1}^{K}|\widehat{p}_{ki} - p_{ki}|$ and the x axis is the sample index i for $i = 1, \ldots, n$.

Supplementary Figure 58.

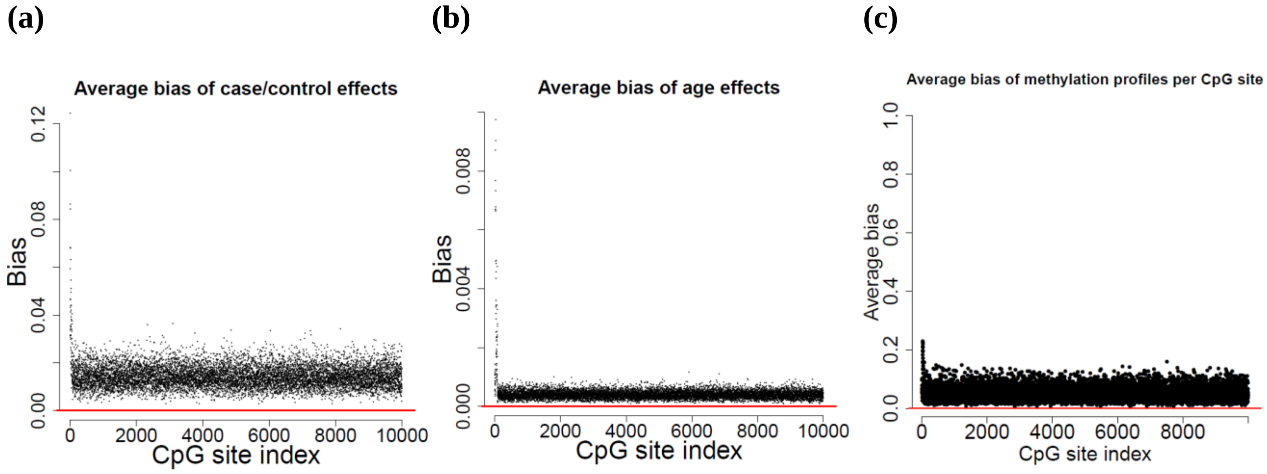

In the “true alternative” case with K=7 and n=180,the average biasplots for (a) the case/control effects and (b) the age effects. In (a), the y axis is the $\frac{1}{K}\sum_{k=1}^{K}|\hat{\beta}_{jk1}-\beta_{jk1}|$ and the x axis is the CpG site index j for $j=1,\dots,m$. In (b), the y axis is the $\frac{1}{K}\sum_{k=1}^{K}|\hat{\beta}_{jk2}-\beta_{jk2}|$ and the x axis is the CpG site index j for $j=1,\dots,m$. In (c), the y axis is the $\frac{1}{K}\sum_{k=1}^{K}|\hat{\mu}_{jk}-\mu_{jk}|$ and the x-axis is the CpG site index j for $j=1,\dots,m$.

Supplementary Figure 59.

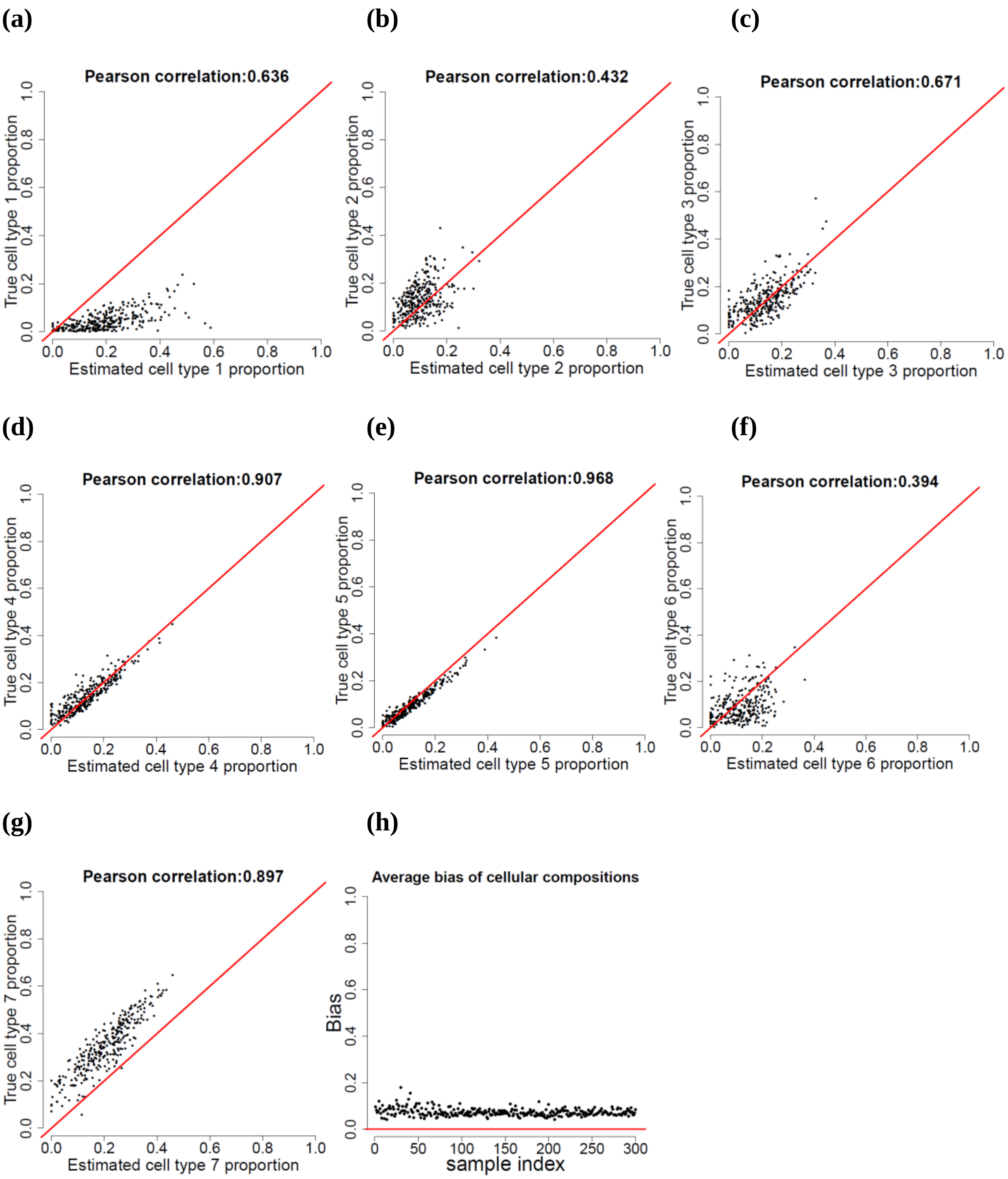

In the "true alternative" case with K=7 and n=300, (a-g) the scatter plots for the estimated cell proportions by HIRE and the underlying truth for (a) cell type 1; (b) cell type 2; (c) cell type 3; (d) cell type 4; (e) cell type 5; (f) cell type 6; and (g) cell type 7. (h) The average biasplot for the cellular compositions, where the y axis is $\frac{1}{K}\sum_{k=1}^{K}|\widehat{p}_{ki} - p_{ki}|$ and the x axis is the sample index i for $i = 1, \dots, n$.

Supplementary Figure 60.

(a) **(b)** **(c)**

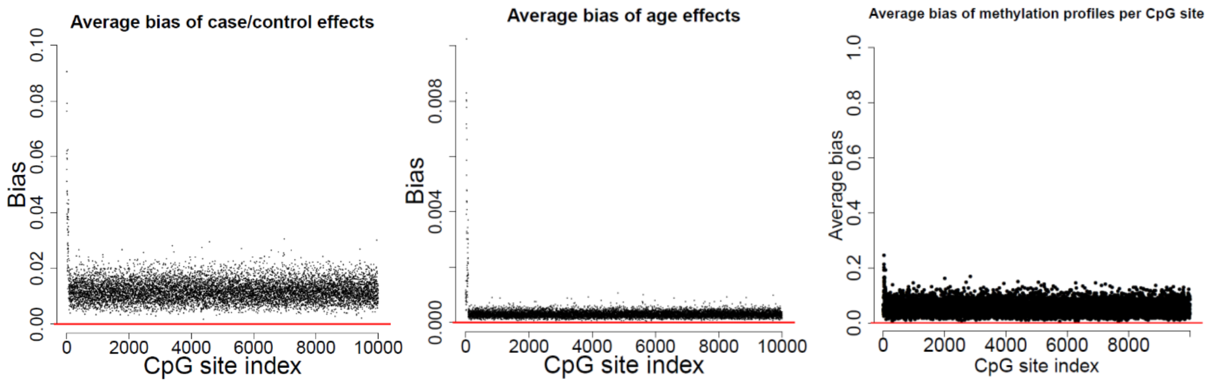

In the “true alternative” case with K=7 and n=300,the average biasplots for (a) the case/control effects and (b) the age effects.In (a), the y axis is the $\frac{1}{K}\sum_{k=1}^{K}|\hat{\beta}_{jk1}-\beta_{jk1}|$ and the x axis is the CpG site index j for $j=1,\ldots,m$. In (b), the y axis is the $\frac{1}{K}\sum_{k=1}^{K}|\hat{\beta}_{jk2}-\beta_{jk2}|$ and the x axis is the CpG site index j for $j=1,\ldots,m$. In (c), the y axis is the $\frac{1}{K}\sum_{k=1}^{K}|\hat{\mu}_{jk}-\mu_{jk}|$ and the x-axis is the CpG site index j for $j=1,\ldots,m$.

Supplementary Figure 61.

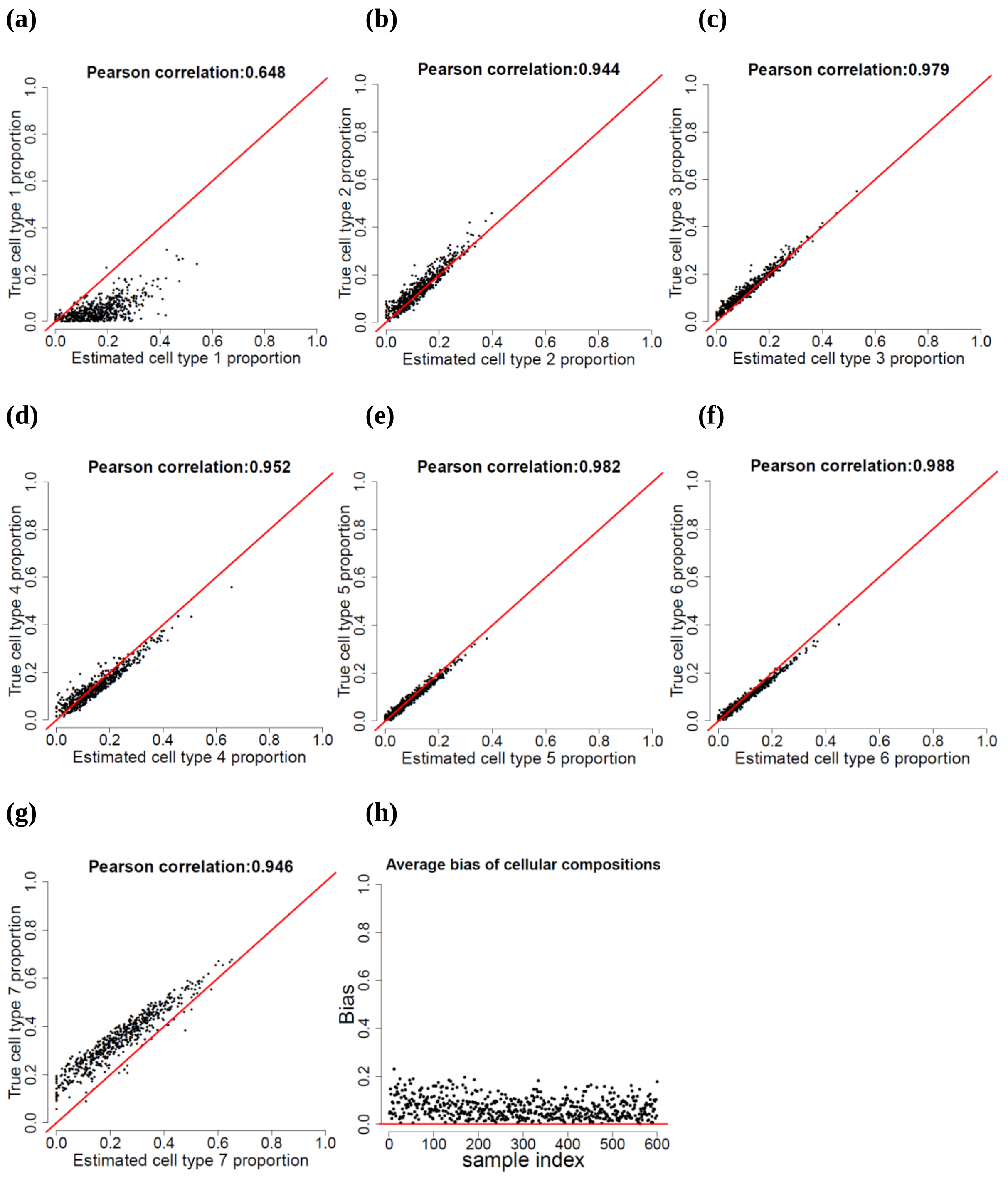

In the "true alternative" case with K=7 and n=600, (a-g) the scatter plots for the estimated cell proportions by HIRE and the underlying truth for (a) cell type 1; (b) cell type 2; (c) cell type 3; (d) cell type 4; (e) cell type 5; (f) cell type 6; and (g) cell type 7. (h) The average biasplot for the cellular compositions.

Supplementary Figure 62.

(a)

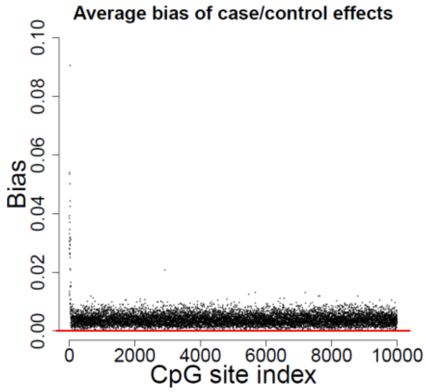

(b)

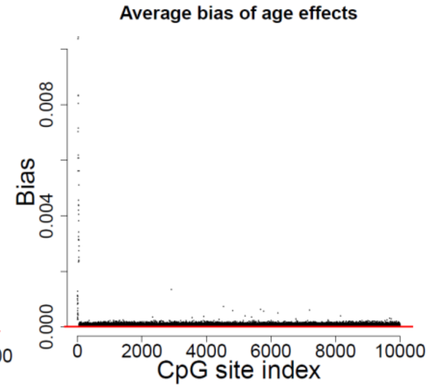

(c)

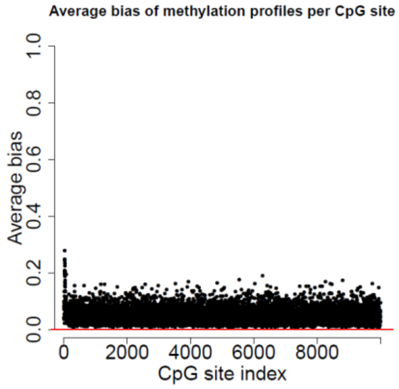

In the “true alternative” case with K=7 and n=600,the average biasplots for (a) the case/control effects and (b) the age effects.In (a), the y axis is the $\frac{1}{K}\sum_{k=1}^{K}|\hat{\beta}_{jk1}-\beta_{jk1}|$ and the x axis is the CpG site index j for $j=1,\dots,m$. In (b), the y axis is the $\frac{1}{K}\sum_{k=1}^{K}|\hat{\beta}_{jk2}-\beta_{jk2}|$ and the x axis is the CpG site index j for $j=1,\dots,m$. In (c), the y axis is the $\frac{1}{K}\sum_{k=1}^{K}|\hat{\mu}_{jk}-\mu_{jk}|$ and the x-axis is the CpG site index j for $j=1,\dots,m$.

Supplementary Figure 63.

(a) **(b)**

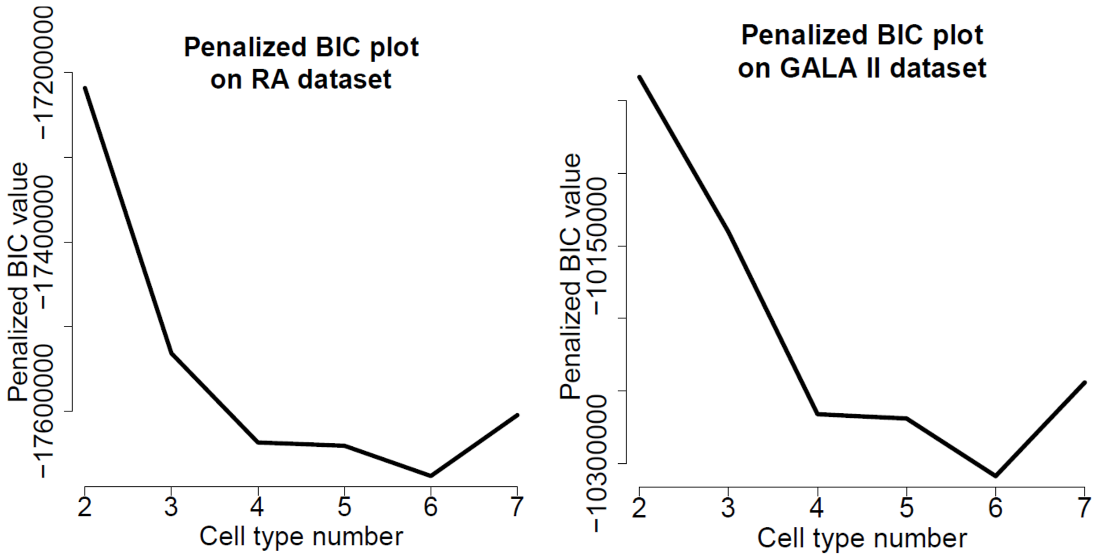

The penalized BIC plots, $pBIC(\alpha)$, for the (**a**) RA dataset and **(b)** GALA II dataset. α is set at 0.01 (see Supplementary Note). The y-axis is the value of $pBIC(\alpha)$, and the x-axis corresponds to the cell type number. $pBIC(\alpha)$attains the minimum at three, six, and six for the simulated dataset, RA dataset, and GALA II dataset, respectively.

Supplementary Figure 64.

(a)

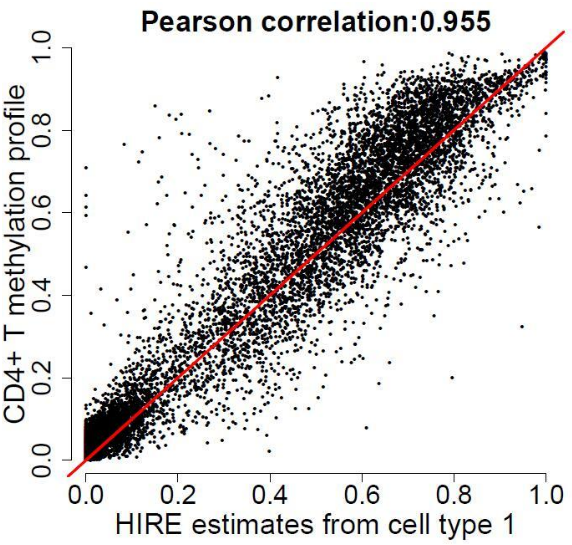

(b)

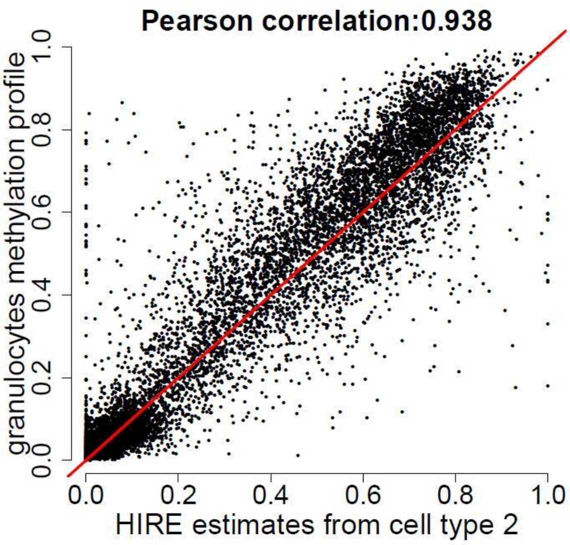

(c)

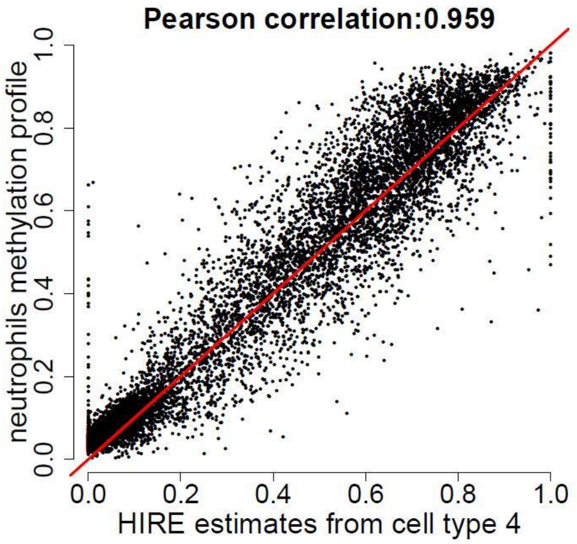

In the RA data set, the scatter plots for the estimated methylation profile by HIRE and its matched reference for (a) cell type 1; (b) cell type 2; and (c) cell type 4.

Supplementary Figure 65.

(a)

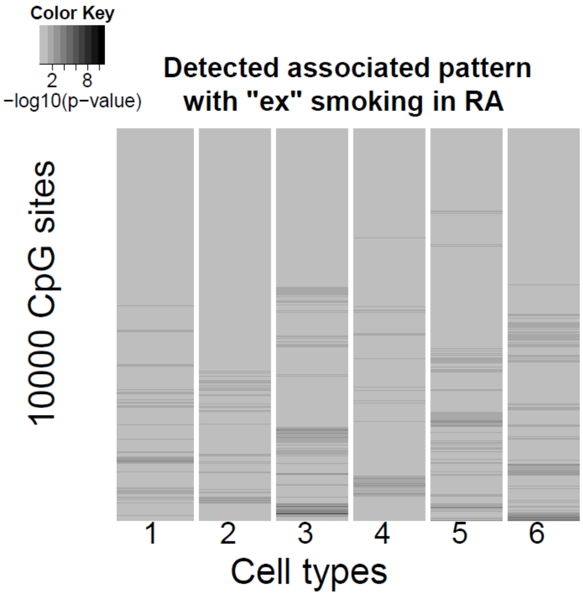

(b)

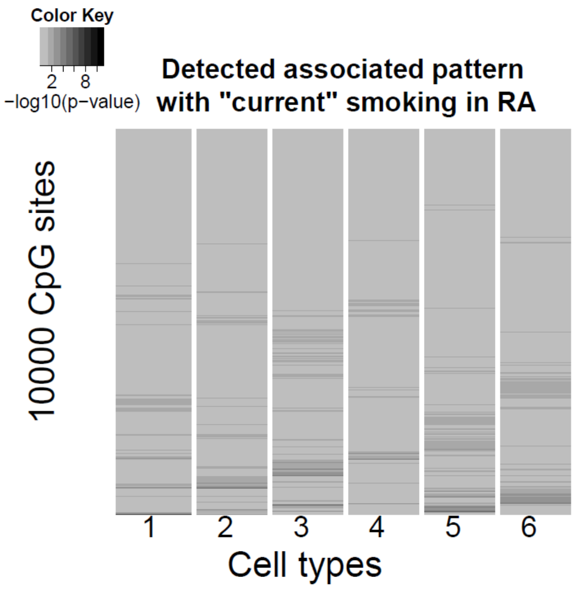

(c)

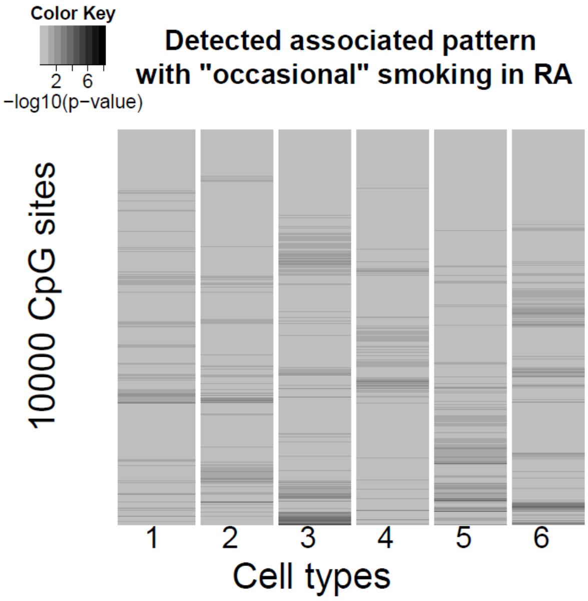

The detectedassociation pattern by HIRE in the RA dataset for three types of smoking history: **(a)** “ex”, **(b)** “current”, and **(c)** “occasional”. In all of the panels, the rows correspond to the CpG sites, and the columns represent the cell types. Each cell's darkness degree represents the significance level at the scale$-\log_{10}(p - value)$.

Supplementary Figure 66.

(a)

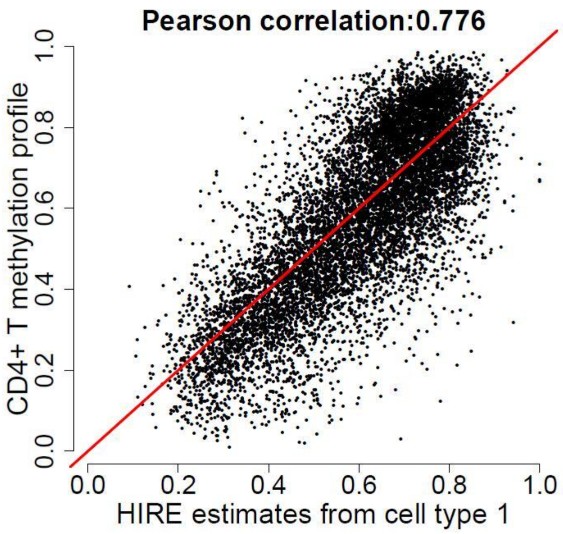

(b)

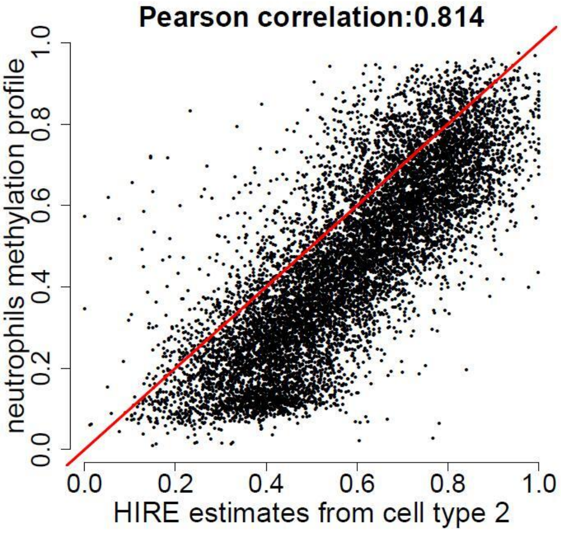

(c)

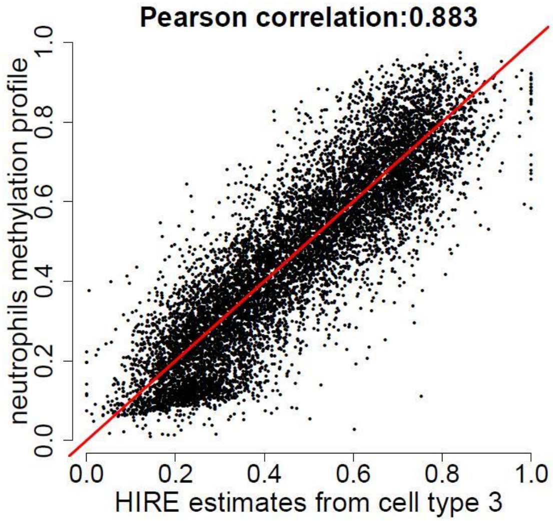

(d)

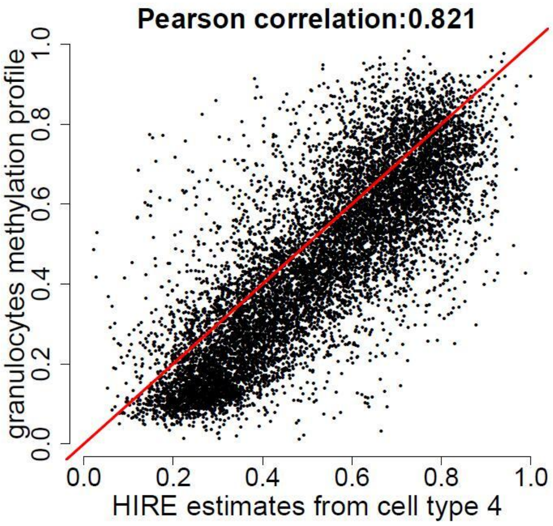

In the GALA II data set, the scatter plots for the estimated methylation profile by HIRE and its matched reference for (a) cell type 1; (b) cell type 2; (c) cell type 3; and (d) cell type 4.

Supplementary Figure 67.

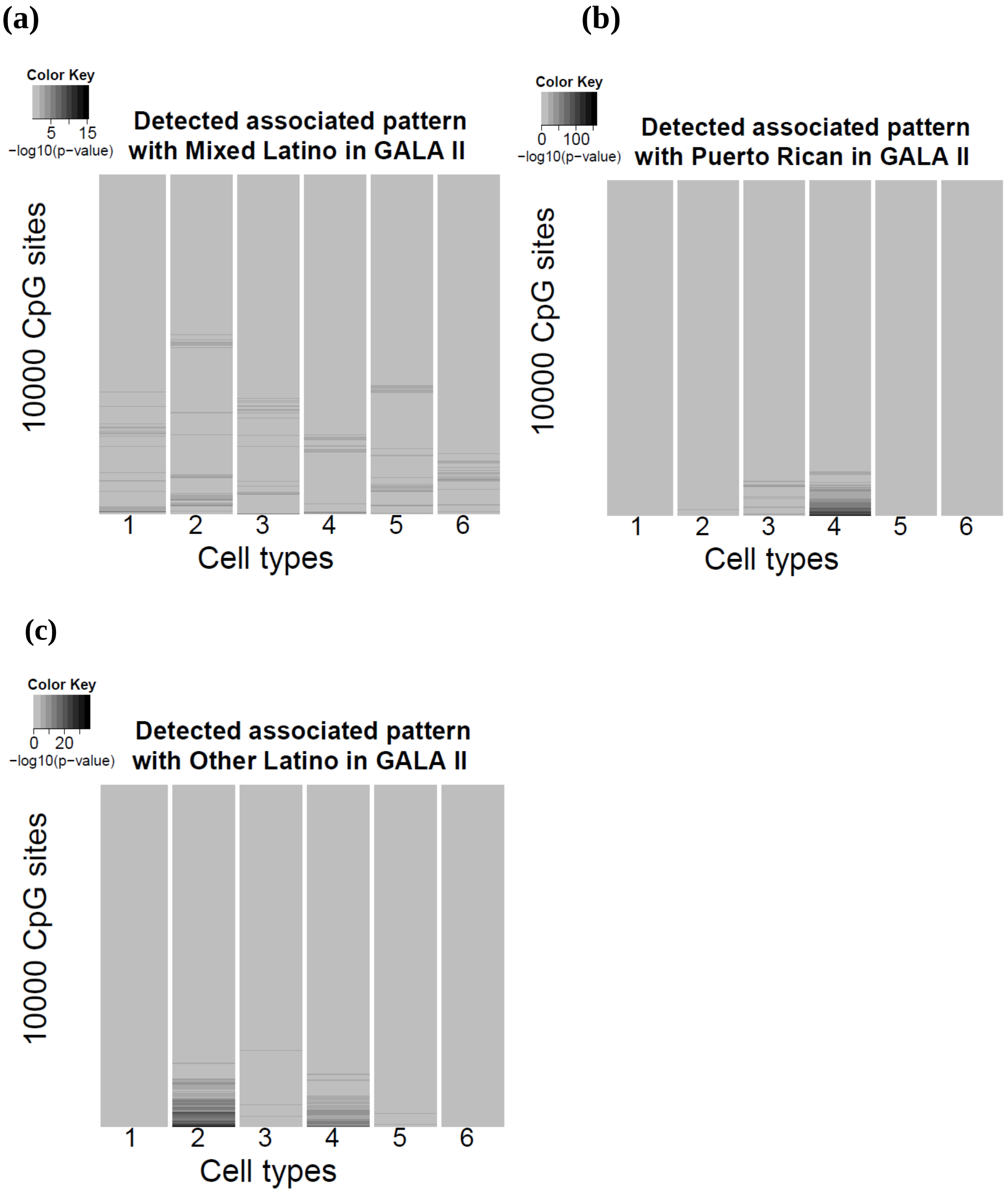

The detectedassociation pattern by HIRE in the GALA II dataset for three types of ethnicity: **(a)** Mixed Latino, **(b)** Puerto Rican, and **(c)** Other Latino. In all of the panels, the rows correspond to the CpG sites, and the columns represent the cell types. Each cell's darkness degree represents the significance level at the scale$-\log_{10}(p-value)$.

Supplementary Figure 68.

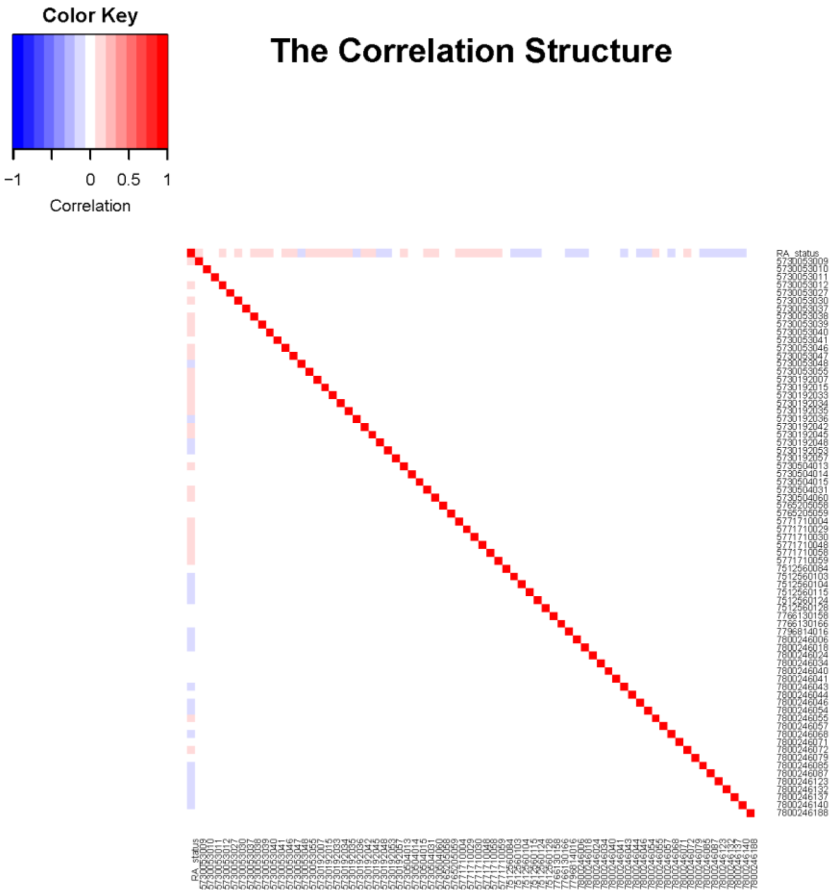

The correlations between the RA status and batch indicators in the RA dataset. In the heatmap, each cell corresponds to the Pearson correlation between its row attribute and its column attribute. The numbers in the columns and rows refer to the batch codes. The maximum of absolute values of correlations between the RA status and the batches is 0.137, so we can use COMBAT to adjust the original RA methylation data for the batch effects.

Supplementary Figure 69.

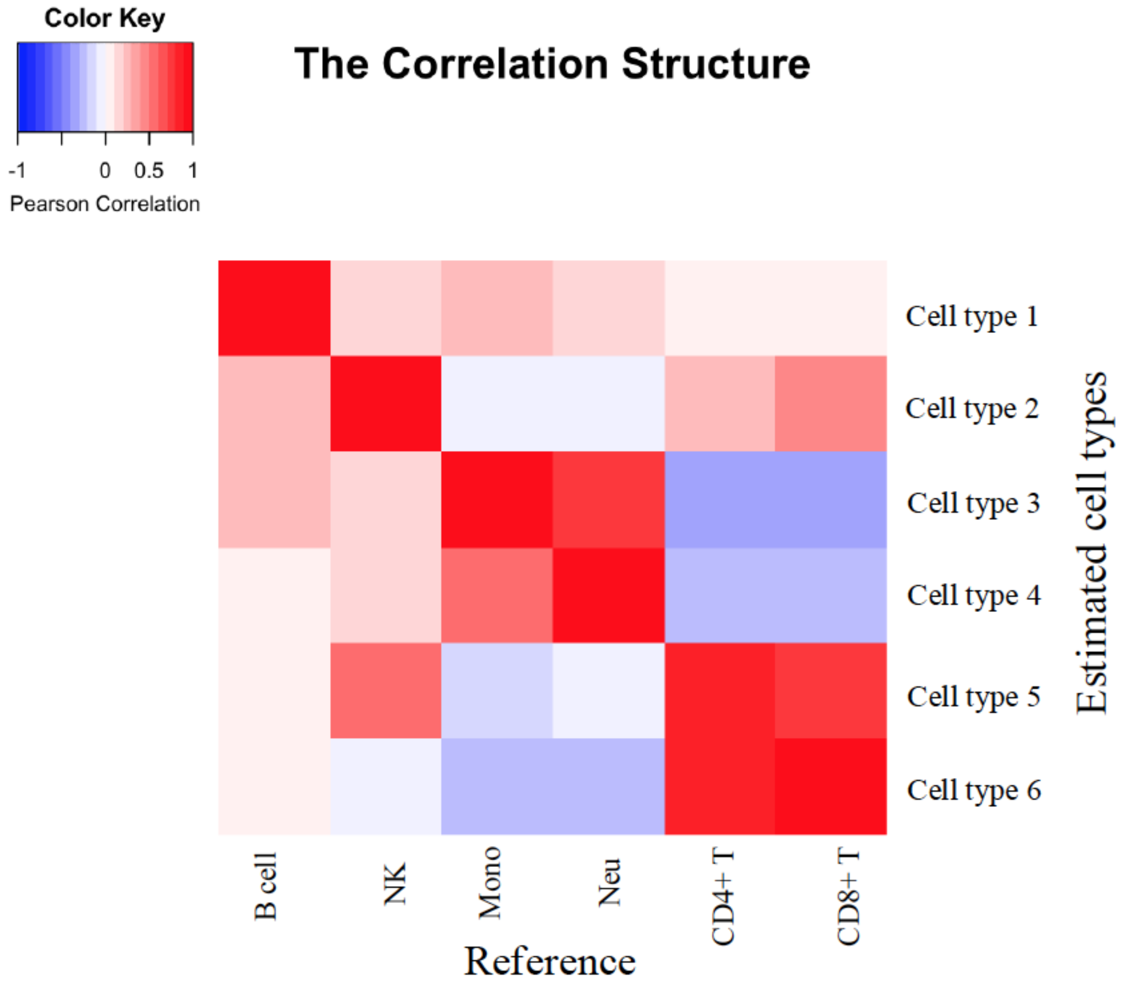

The heatmap of the Pearson correlation matrix between the estimated cell types and the biological cell types for the semi-simulated dataset. The red indicates positive values; the blue represents negative values; and values near zero are in white.

Supplementary Figure 70.

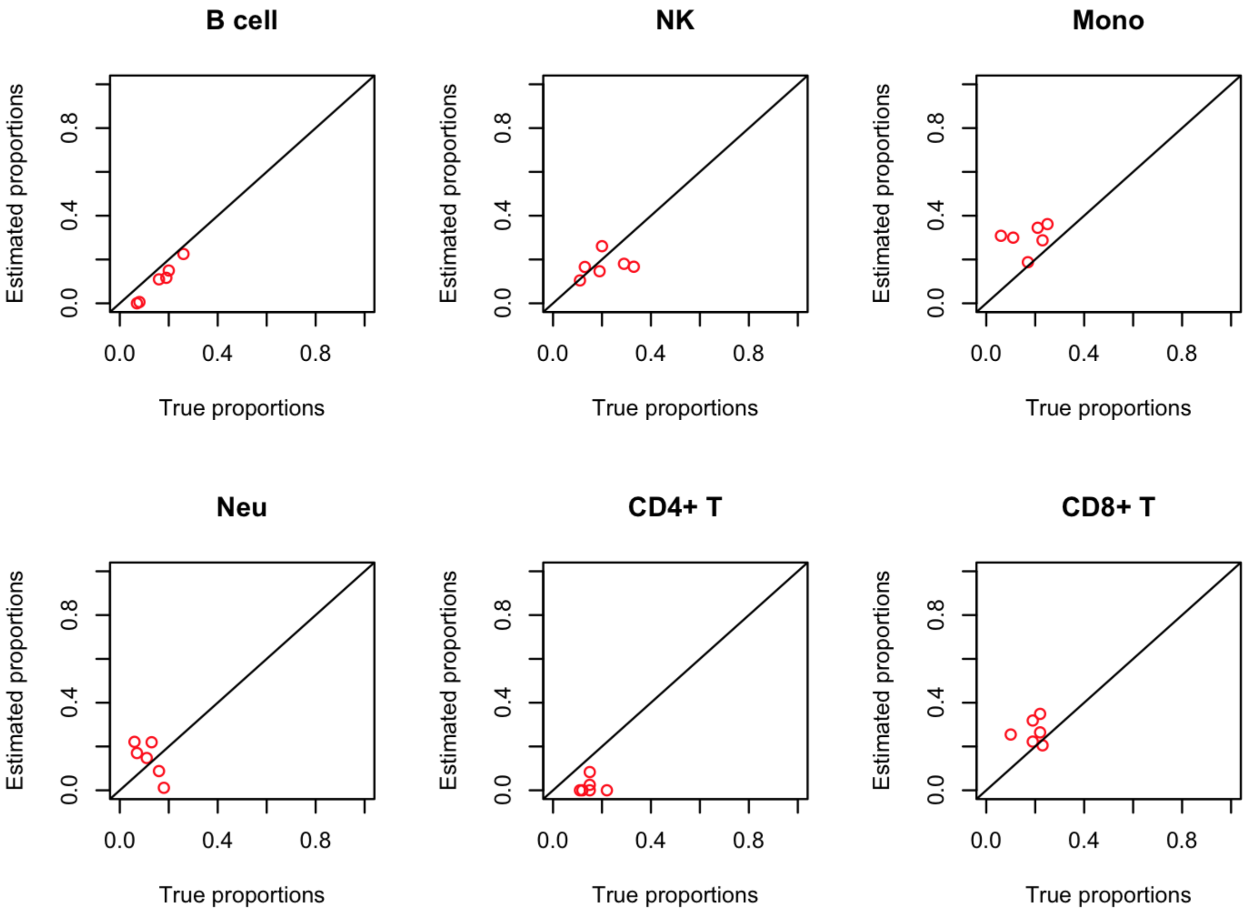

The scatter plots for the estimated cell proportions versus the underlying truth for the six mix samples with known cell type mixing proportions in the semi-simulated dataset. Each panel corresponds to one of the six cell types used for mixing: B cell, NK, monocytes, neutrophils, CD4+ T, and CD8+ T.

Supplementary Figure 71.

(a)

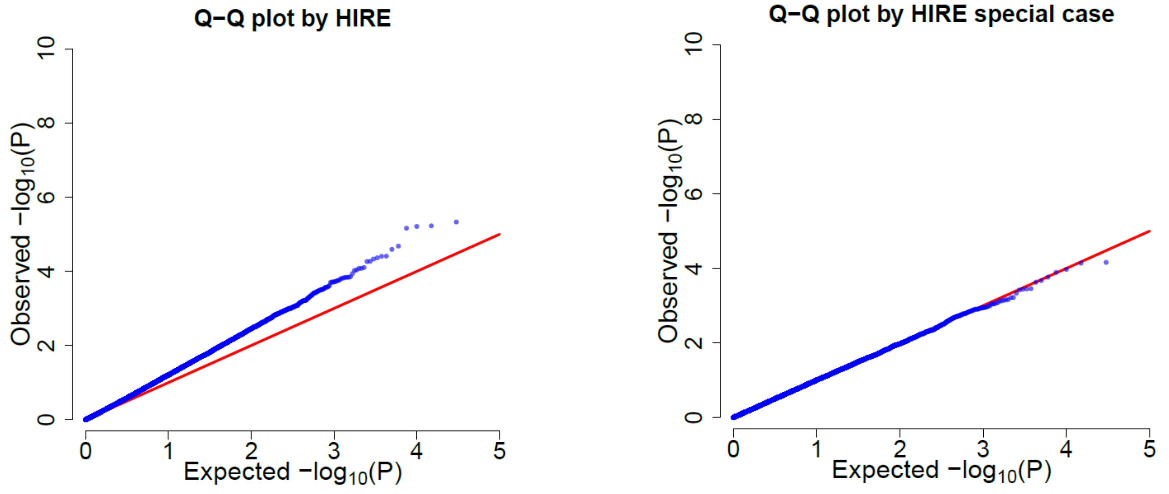

(b)

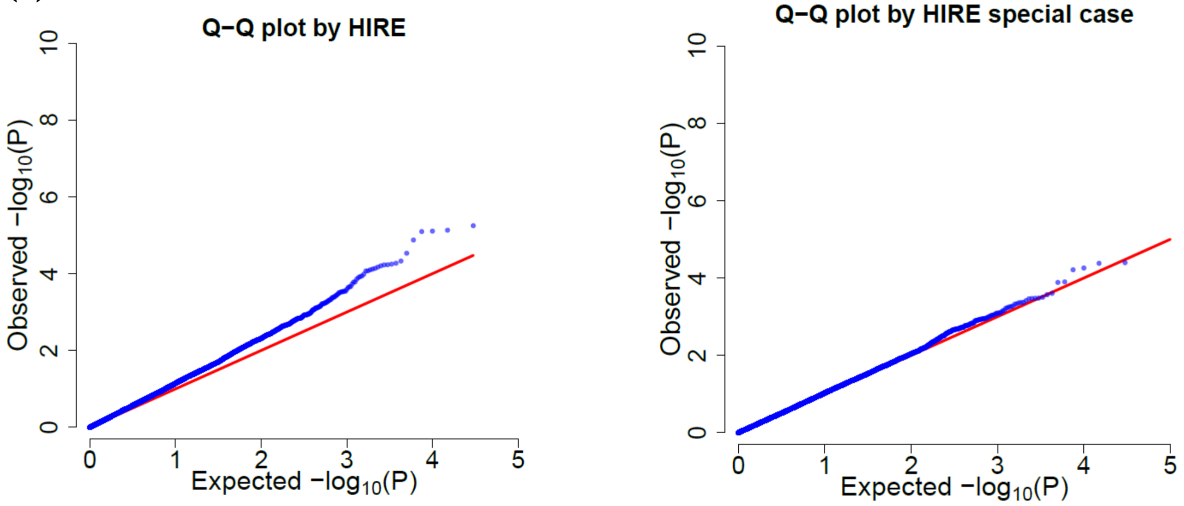

(c)

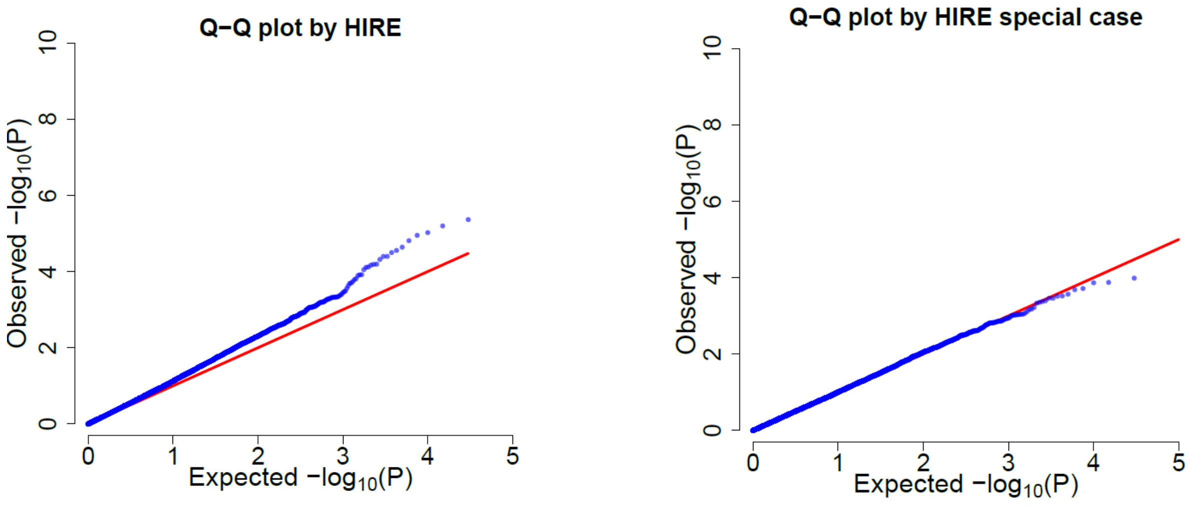

The Q-Q plots by HIRE and its special case for the "true null" simulation settings: (a) K=3 and n=180; (b) K=3 and n=300; (c) K=3 and n= 600. The Q-Q lines by HIRE's special case are near the y = x, thus controlling the inflation very well.

Supplementary Figure 72.

(a)

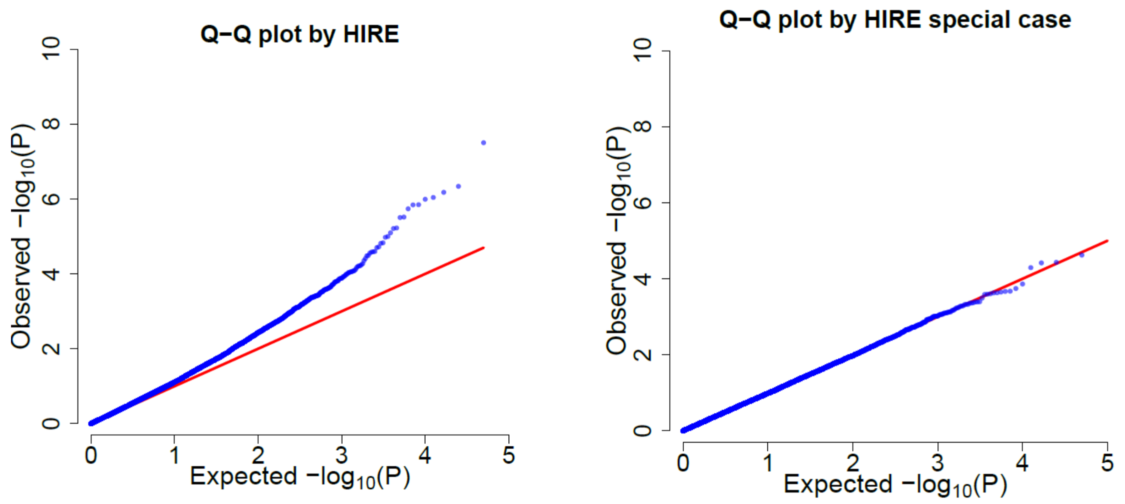

(b)

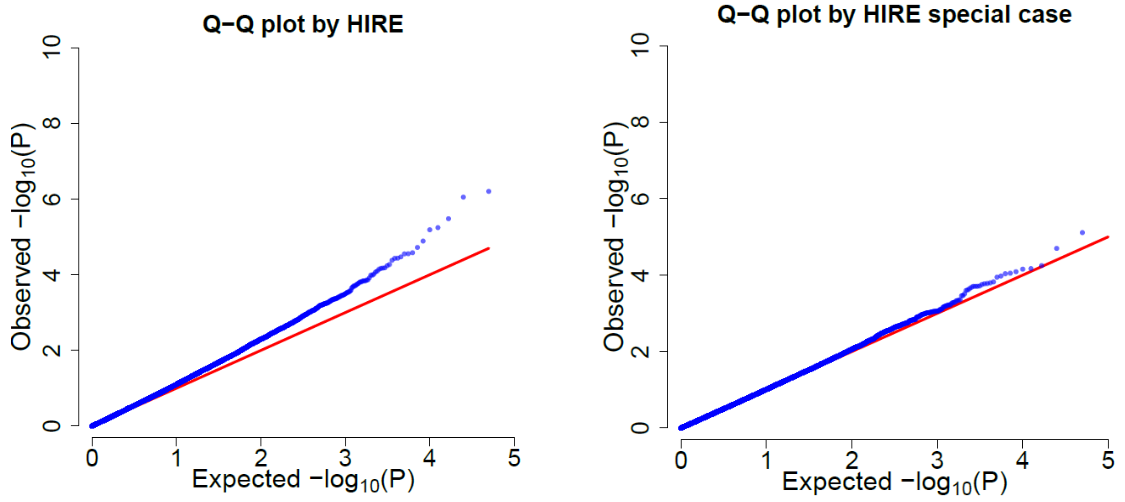

(c)

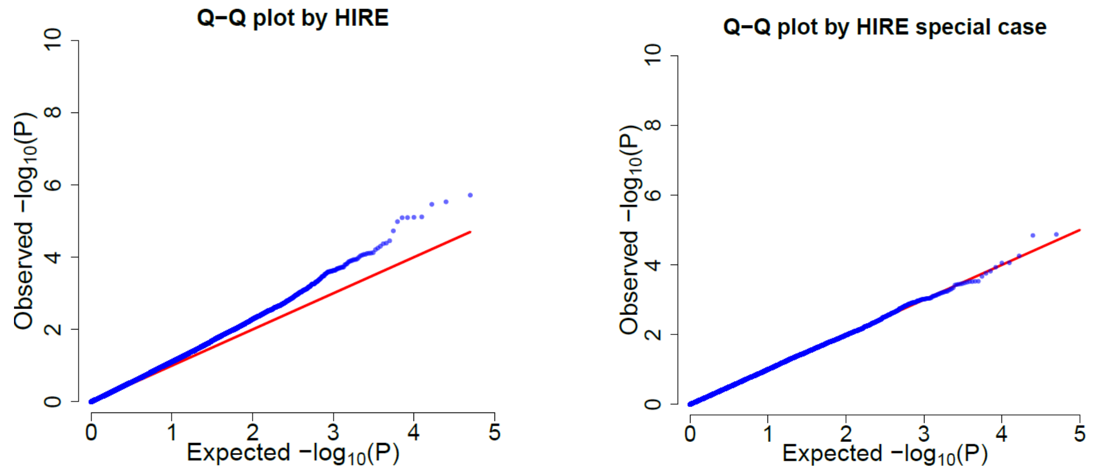

The Q-Q plots by HIRE and its special case for the "true null" simulation settings: (a) K=5 and n=180; (b) K=5 and n=300; (c) K=5 and n= 600. The Q-Q lines by HIRE's special case are near the y = x, thus controlling the inflation very well.

Supplementary Figure 73.

(a)

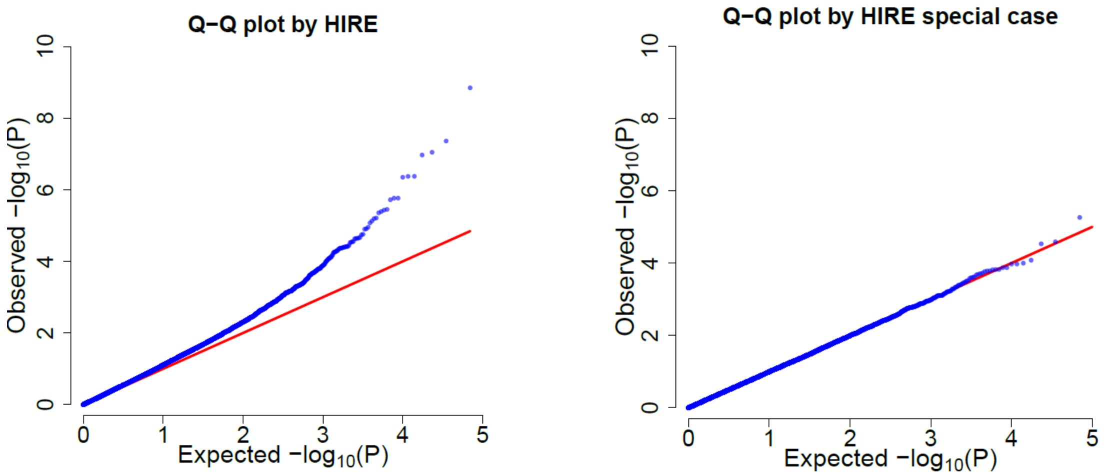

(b)

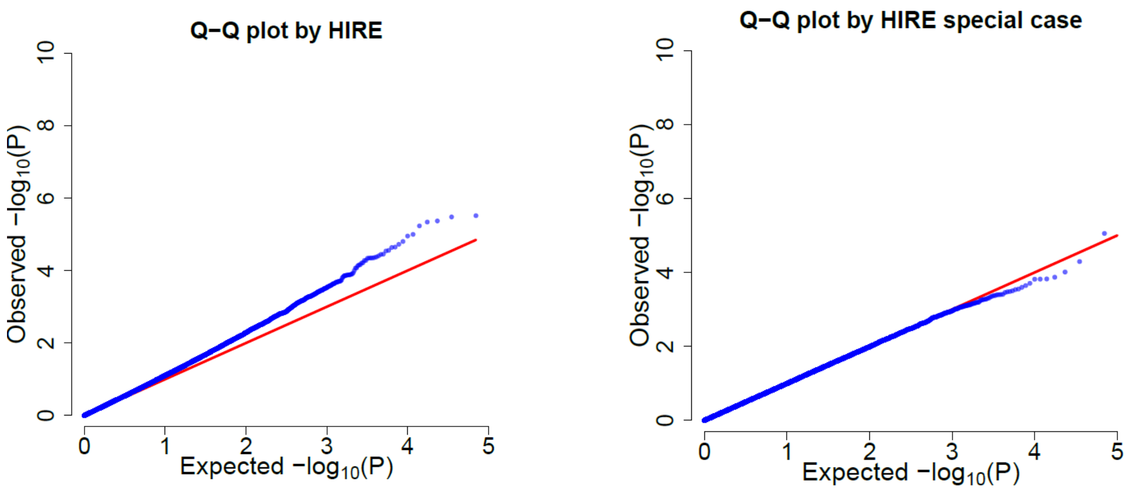

(c)

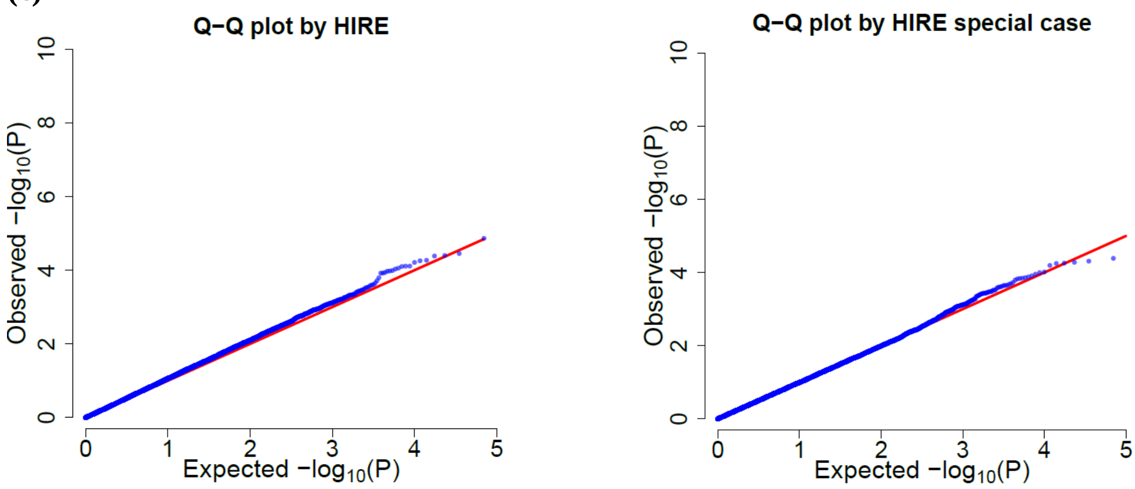

The Q-Q plots by HIRE and its special case for the "true null" simulation settings: (a) K=7 and n=180; (b) K=7 and n=300; (c) K=7 and n= 600. The Q-Q lines by HIRE's special case are near the y = x, thus controlling the inflation very well.

Supplementary Figure 74.

(a)

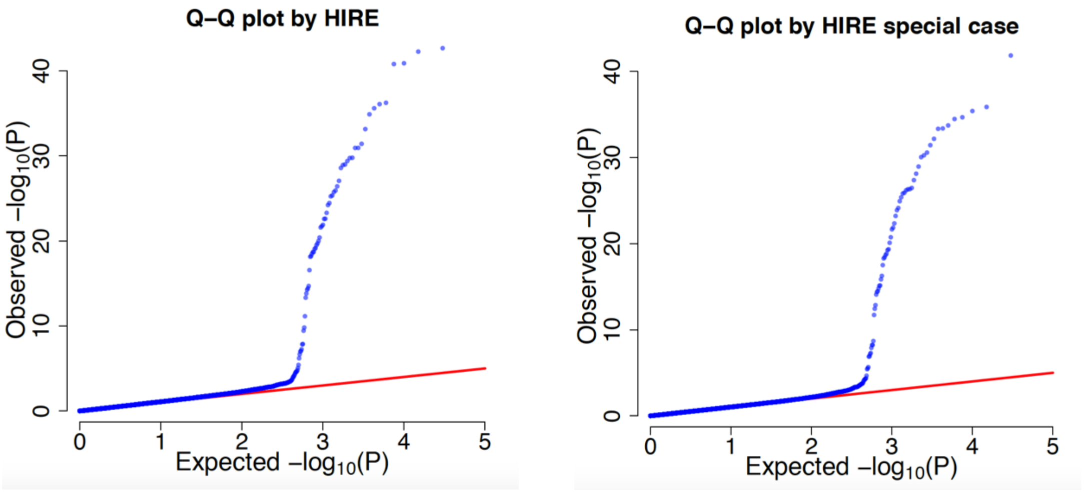

(b)

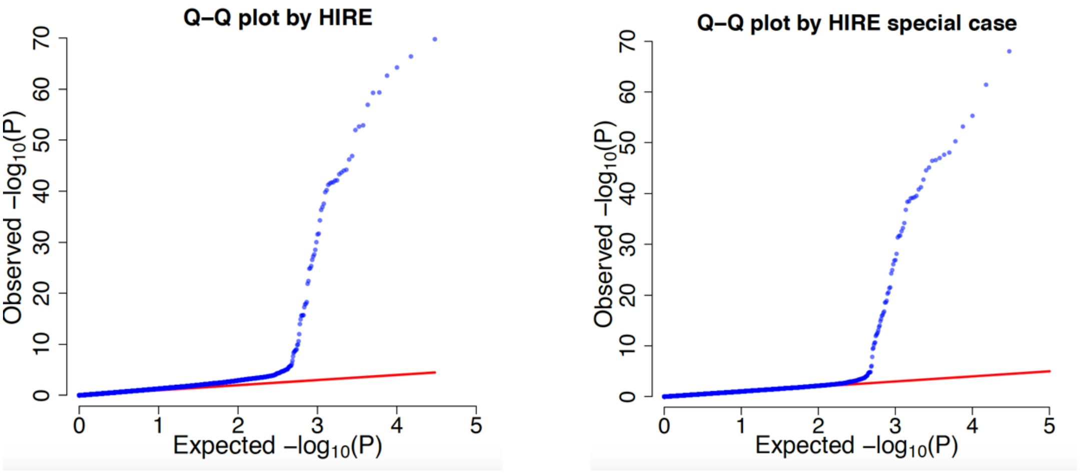

(c)

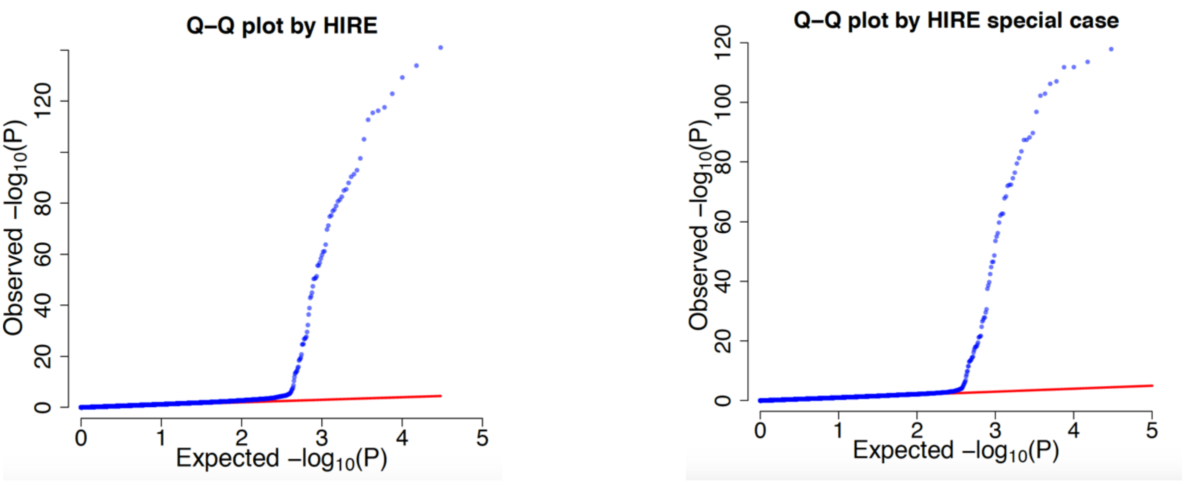

The Q-Q plots by HIRE and its special case for the "true alternative" simulation settings: (a) K=3 and n=180; (b) K=3 and n=300; (c) K=3 and n= 600. They have very similar behaviors.

Supplementary Figure 75.

(a)

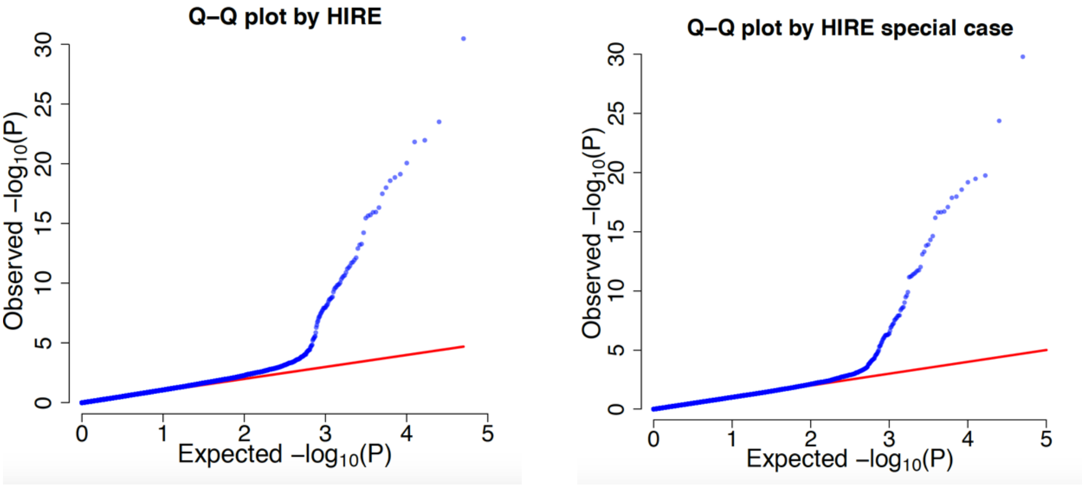

(b)

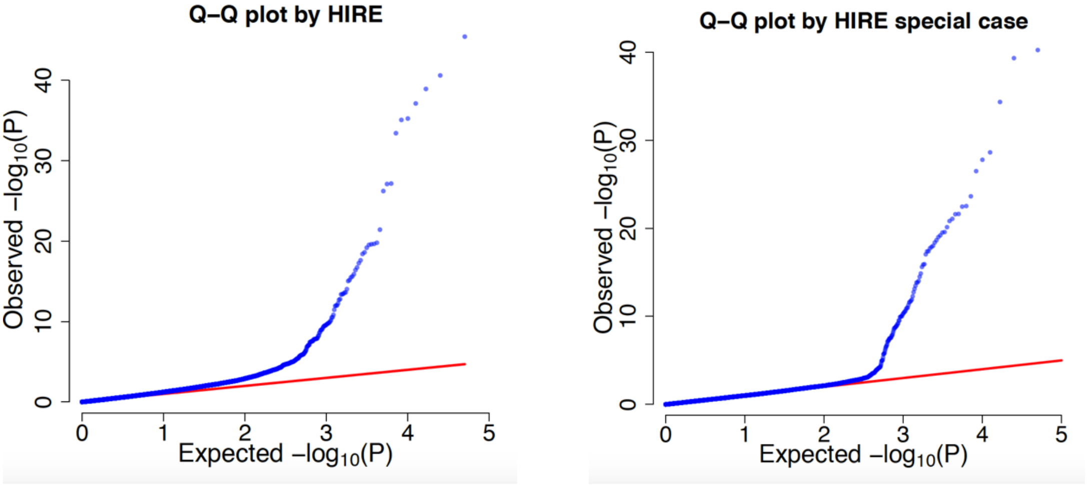

(c)

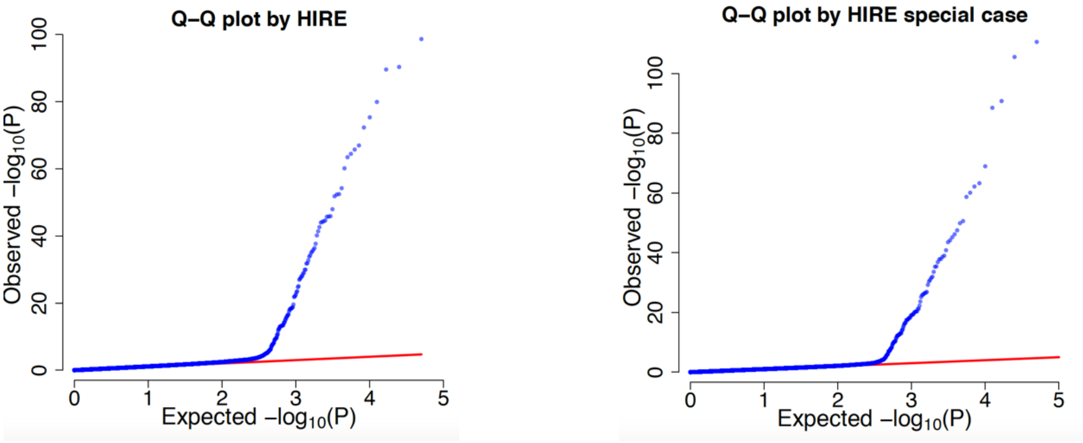

The Q-Q plots by HIRE and its special case for the "true alternative" simulation settings: (a) K=5 and n=180; (b) K=5 and n=300; (c) K=5 and n= 600. They have very similar behaviors.

Supplementary Figure 76.

(a)

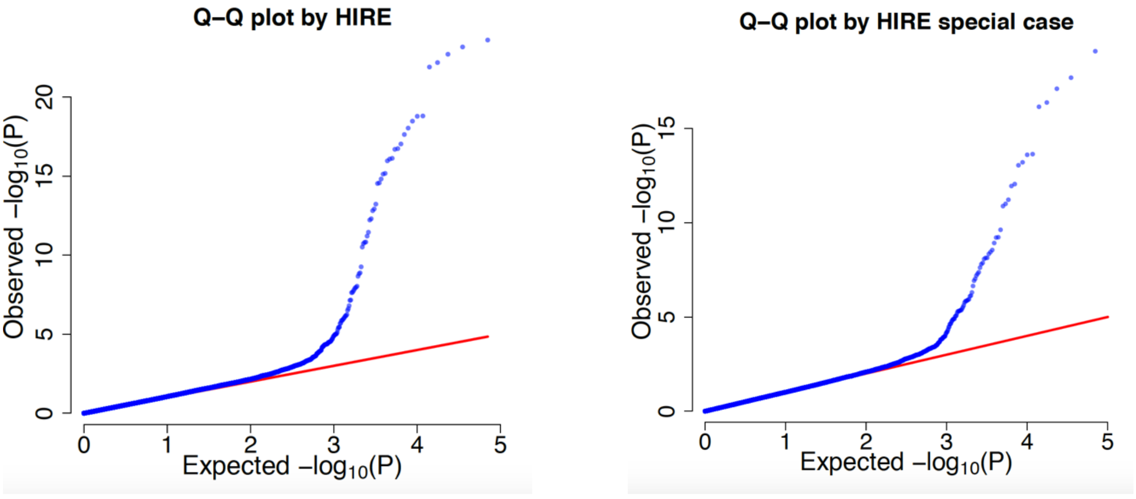

(b)

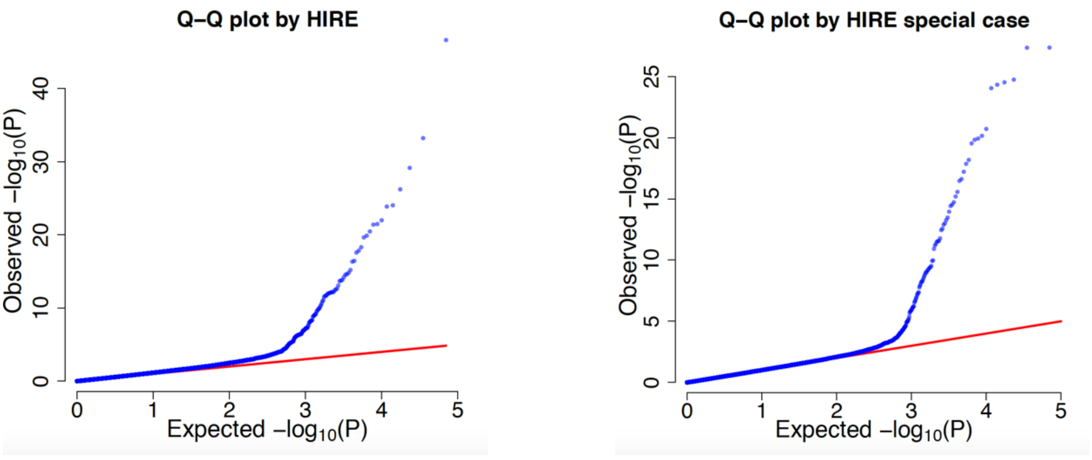

(c)

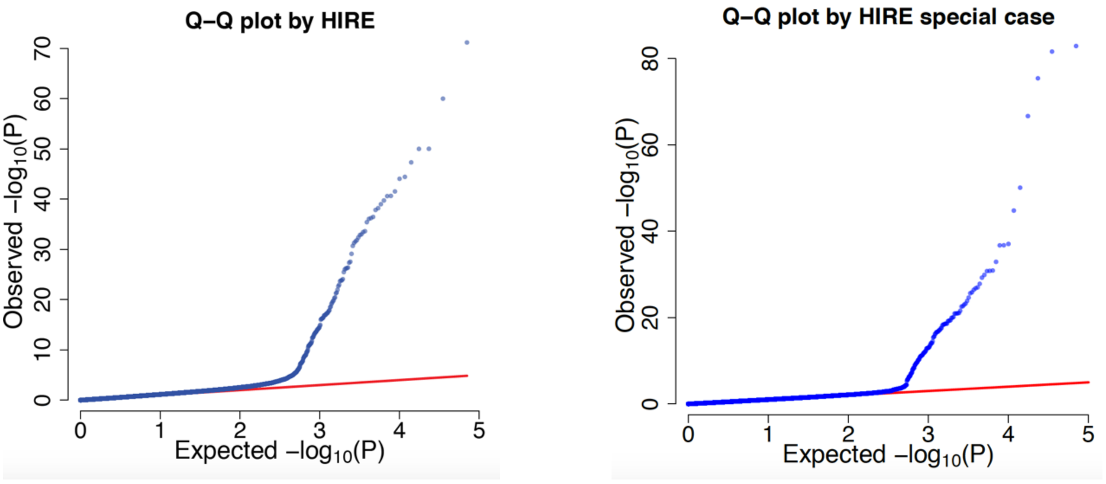

The Q-Q plots by HIRE and its special case for the "true alternative" simulation settings: (a) K=7 and n=180; (b) K=7 and n=300; (c) K=7 and n= 600. They have very similar behaviors.

Supplementary Table 1.

Cell type number		n=180	n=300	n=600
K=3	FPR	0.13%	0.04%	0.03%
	TPR	90%	93.33%	100%
K=5	FPR	0.03%	0.01%	0
	TPR	62%	92%	92%
K=7	FPR	0	0.00%	0.01%
	TPR	35%	63.33%	80%

The FPRs and TPRs of HIRE in different sample sizes and cell type numbers when nearby CpG sites are correlated with $\rho = 0.8$.

Supplementary Table 2.

Cell type number		n=180	n=300	n=600
K=3	FPR	0.10%	0.01%	0.04%
	TPR	90%	93.33%	100%
K=5	FPR	0.01%	0.09%	0
	TPR	56%	86%	92%
K=7	FPR	0	0.00%	0.02%
	TPR	43.33%	63.33%	76.67%

The FPRs and TPRs of HIRE in different sample sizes and cell type numbers when nearby CpG sites are correlated with ρ = 0.6.

Supplementary Table 3.

Cell type number		n=180	n=300	n=600
K=3	FPR<br>TPR	0<br>90%	0.02%<br>93.33%	0.03%<br>100%
K=5	FPR<br>TPR	0.01%<br>58%	0.07%<br>82%	0<br>90%
K=7	FPR<br>TPR	0<br>43.33%	0.00%<br>68.33%	0.01%<br>81.67%

The FPRs and TPRs of HIRE in different sample sizes and cell type numbers when nearby CpG sites are correlated with $\rho = 0.4$.

Supplementary Table 4.

Cell type number		n=180	n=300	n=600
K=3	FPR<br>TPR	0.01%<br>98.67%	0.00%<br>96.67%	0.03%<br>100%
K=5	FPR<br>TPR	0.00%<br>66%	0.01%<br>89.6%	0.01%<br>98.4%
K=7	FPR<br>TPR	0<br>43%	0.00%<br>63.33%	0.04%<br>82.67%

The FPRs and TPRs of HIRE in different sample sizes and cell type numbers when nearby CpG sites are uncorrelated (extracted from Table 1 in the manuscript).

Supplementary Table 5.

ID	IlluminaGene	Significant_in
cg15418221	PGK1	cell type 6 affected by RA
cg08076861	ZNF275	cell type 4 affected by RA
cg16756025	ZNF275	cell type 4 affected by RA
cg12158214	FAM104B	cell type 6 affected by RA
cg01813294	NHS	cell type 6 affected by RA
cg18112782	OTUD5	cell type 5 affected by RA
cg27609596	PTCHD1	cell type 4 affected by RA
cg07910525	NUDT10	cell type 5 affected by RA
cg16954455	ARX	cell type 5 affected by RA
cg27471304		cell type 5 affected by RA
cg03580328	SLC16A2	cell type 5 affected by RA
cg16097380	LOC100303728;SLC25A5	cell type 6 affected by RA
cg13189687	MECP2	cell type 5 affected by RA
cg27665489	NUDT10	cell type 5 affected by RA
cg03979311	GZMK	cell type 3 affected by RA
cg16967668	41158	cell type 3 affected by RA
cg07973464	NHS	cell type 6 affected by RA
cg05442715	APOO;CXorf58	cell type 6 affected by RA
cg12548693	MIR223	cell type 3 affected by RA
cg23612178	TSPYL2	cell type 6 affected by RA
cg15074974		cell type 3 affected by RA
cg26672162	CFP	cell type 3 affected by RA
cg03146219	NADSYN1	cell type 3 affected by RA
cg03672021	FLNA	cell type 6 affected by RA
cg10645377	ATP6AP1	cell type 3 affected by RA
cg13785123	ENO1	cell type 3 affected by RA
cg06055803	OCRL	cell type 3 affected by RA
cg21957905	SORCS2	cell type 3 affected by RA
cg05398700	WDR20	cell type 3 affected by RA
cg12213414	LYG2	cell type 3 affected by RA
cg23169883	ADAMTS14	cell type 3 affected by RA
cg00439981	FAM120B	cell types 2,3 affected by RA
cg18188328	JARID2	cell type 3 affected by RA
cg02193580	FHL1	cell type 3 affected by RA
cg06218079	TBCD	cell type 3 affected by RA
cg00446123	LIME1	cell type 3 affected by RA
cg05971678	CHST15	cell type 3 affected by RA
cg04858164	TCF12	cell types 2,3 affected by RA
cg00660167		cell type 3 affected by RA
cg20805133	PDCD1	cell type 3 affected by RA
cg09351156	RNF144B	cell type 3 affected by RA
cg03637218	AP3S1	cell type 3 affected by RA
cg21875980	EGLN1	cell type 3 affected by RA

cg10665891		cell type 3 affected by RA
cg07275179	ATXN7	cell type 3 affected by RA
cg25919177		cell type 3 affected by RA
cg19081101	CHI3L1	cell type 3 affected by RA
cg22203628		cell type 3 affected by RA
cg23478547		cell type 3 affected by RA
cg16313807	HSBP1L1	cell type 3 affected by RA
cg08450017	CXCR6;FYCO1	cell types 2,3,4 affected by RA
cg14516183	SEC22A	cell type 3 affected by RA
cg17733714		cell type 3 affected by RA
cg20107506		cell type 3 affected by RA
cg24131359	CPM	cell type 3 affected by RA
cg22712983	PNKD	cell type 3 affected by RA
cg19023589	TMCO3	cell types 2,3 affected by RA
cg15056572	MIR505	cell type 3 affected by RA
cg23568192		cell type 3 affected by RA
cg21743623	ATP2C1	cell types 2,3 affected by RA
cg10751070		cell type 3 affected by RA
**cg06373940**	**ERCC3**	**cell types 2,3,4 affected by RA**
cg25197500	WDR45L	cell type 3 affected by RA
cg07734159	VCL	cell type 3 affected by RA
cg09467433		cell type 3 affected by RA
cg09993145	RUNX3	cell type 3 affected by RA
cg02927682	SSBP3	cell types 2,3 affected by RA
cg05418105		cell type 3 affected by RA
cg12073436		cell types 2,3 affected by RA
cg08279189	TRIM27	cell type 3 affected by RA
cg02185190	SLC25A43	cell type 3 affected by RA
cg17791799	PROM1	cell type 3 affected by RA
cg07658508	SLC26A1	cell type 3 affected by RA
cg15029631	SCN8A	cell type 3 affected by RA
cg14345154	TTC8	cell type 3 affected by RA
cg05060607	C3AR1	cell type 3 affected by RA
cg09447675		cell type 3 affected by RA
cg25344401	FOXK1	cell types 2,3 affected by RA
cg01942558	TNFAIP6	cell type 3 affected by RA
cg17828255		cell type 3 affected by RA
cg07472373	DRGX	cell type 3 affected by RA

The 81 CpG sites significantly associated with the RA status in RA dataset. The CpG site in bold is discussed in the main text. The FWER is controlled to be less than 0.01 using the Bonferroni correction. The cell type information in which the CpG site is significant is also provided in the third column.

Supplementary Table 6.

ID	IlluminaGene	Significant_in (FWER < 0.01)
cg10717149	SLC25A14	cell type 3 affected by smoking status "ex"
**cg05575921**	**AHRR**	**cell types 2,3,4 affected by smoking status "current"**
cg23169883	ADAMTS14	cell type 3 affected by smoking status "occasional"
cg12073436		cell type 5 affected by smoking status "current"
cg14345154	TTC8	cell type 3 affected by smoking status "occasional"

The five CpG sites significantly associated with the smoking history in the RA dataset. The CpG site in bold is discussed in the main text. The FWER is controlled to be less than 0.01 using the Bonferroni correction. The cell type information in which the CpG site is significant is also provided in the third column.

Supplementary Table 7.

Pathway name	Description	FDR q-value
PID CMYB PATHWAY	C-MYB transcription factor network	1.18×10^{-4}
REACTOME HEMOSTASIS	Genes involved in Hemostasis	5.42×10^{-4}
REACTOME IMMUNE SYSTEM	Genes involved in Immune System	1.18×10^{-4}
REACTOME INFLAMMASOMES	Genes involved in Inammasomes	1×10^{-3}
REACTOME FACTORS INVOLVED IN MEGAKARYOCYTE DEVELOP-MENT AND PLATELET PRODUC-TION	Genes involved in Factors involved in megakaryocyte development and platelet production	4.63×10^{-3}
PID P53 DOWNSTREAM PATHWAY	Direct p53 effectors	9.55×10^{-3}
REACTOME THE ROLE OF NEF IN HIV1 REPLICATION AND DISEASE PATHOGENESIS	Genes involved in the role of Nef in HIV-1 replication and disease pathogenesis	1.25×10^{-2}
PID IGF1 PATHWAY	IGF1 pathway	1.35×10^{-2}
RECTOME NUCLEOTIDE BINDING DOMAIN LEUCINE RICH REPEAT CONTAINING RECEPTOR NLR SIG-NALING PATHWAYS	Genes involved in nucleotide-binding domain, leucine rich repeat containing receptor (NLR) signaling pathways	4.33×10^{-2}

The gene set enrichment analysis (GSEA) for HIRE in the GALA II dataset. The genes that harbor the risk-CpG sites found by HIRE were used as the input in GSEA. The used basis gene sets are the canonical pathways (CP). Significant pathways with false discovery rate less than 0.05 and their associated information are shown.

Supplementary Table 8.

Pathway name	Description	FDR q-value
KEGG CARDIAC MUSCLE CONTRACTION	Cardiac muscle contraction	2.71×10^{-2}

The gene set enrichment analysis (GSEA) for ReFACTor in the GALA II dataset. The genes that harborthe risk-CpG sites found by ReFACTor were used as the input in GSEA. The used basis gene sets are the canonical pathways (CP). Significant pathways with false discovery rate less than 0.05 and their associated information are shown.

Supplementary Table 9.

HIRE	Cell type number		n=90	n=120	n=150	n=180	n=300	n=600
True nulls	K=3	FPR	0.08%	0	0.00%	0	0.00%	0
	K=5	FPR	0.01%	0.01%	0.03%	0.05%	0.01%	0
	K=7	FPR	0.00%	0	0.1%	0.00%	0.00%	0
True alternatives	K=3	FPR	0.01%	0.03%	0.01%	0.01%	0.00%	0.03%
		TPR	82.67%	93.33%	94%	98.67%	96.67%	100%
	K=5	FPR	0	0.01%	0.00%	0.00%	0.01%	0.01%
		TPR	36%	52%	55.6%	66%	89.6%	98.4%
	K=7	FPR	0	0.00%	0.00%	0	0.00%	0.04%
		TPR	12.67%	23.33%	36.33%	43%	63.33%	82.67%

The performances of HIRE with different cell type numbers K and various sample sizes n. In each setting, the average of TPR/FPR is shown based on five replicates.

Supplementary Table 10.

Disease Status	n=180	n=300	n=600
K=3	96.67%	96.67%	100%
K=5	58%	82%	94%
K=7	30%	53.33%	86.67%
Age	n=180	n=300	n=600
K=3	96.67%	100%	100%
K=5	98%	98%	100%
K=7	65%	85%	98.33%

The power of HIRE's special case for the disease status and the age under the “true alternative” simulation settings.

Supplementary Notes

In this section, we give a numerical example to illustrate the importance of treating cellular compositions as multiplicative effects. Let us consider one CpG site and assume that there exist two cell types. Table S1 shows the cellular compositions $\mathbf{p}_i = (p_{1i}, p_{2i})$, the cell-type methylation profiles $\mathbf{u}_i = (u_{i1}, u_{i2})$, and the observed methylation values for two controls and three cases. The proportion of cell type 1 is low in the controls and high in the cases. Moreover, the given CpG site is hypermethylated in cell type 1 in cases but not differentially methylated in cell type 2. Here, O_i is calculated as $u_{i1}p_{1i} + u_{i2}p_{2i}$. Denoting the case/control status of sample i by x_i, then $x_i = 1$ if it is a case and $x_i = 0$ if it is a control.

controls					cases				
O_i	p_{1i}	u_{i1}	p_{2i}	u_{i2}	O_i	p_{1i}	u_{i1}	p_{2i}	u_{i2}
0.9	0.1	0	0.9	1	1	0.9	1	0.1	1
0.8	0.2	0	0.8	1	1	0.8	1	0.2	1
					1	0.7	1	0.3	1

Table S1: The data structure in a toy example, where O_i is the observed methylation value of sample i, p_{1i} is sample i's proportion of cell type 1, u_{i1} is the methylation profile of cell type 1 for sample i, and so forth. $O_i = u_{i1}p_{1i} + u_{i2}p_{2i}$.

Despite that existing deconvolution methods actually estimate $\mathbf{p}_i$s with biases, let us assume that the true cell type proportion $\mathbf{p}_i$ is known for now. If we treat $\mathbf{p}_i$ as additive effects as in RefFreeEWAS and other existing methods, we need to regress O_i on x_i and p_{1i} for $i = 1, \ldots, 5$ with the intercept term:

$$O_i = \alpha + \tau \cdot x_i + M \cdot p_{1i} + \epsilon_i. \quad (1)$$

As we are interested in whether there is any association between this CpG site and the case/control status, we want to infer whether τ, the regression coefficient of x_i, is zero or not. However, the p-value of τ is 0.275, so we do not have enough evidence to reject the null hypothesis $H_0 : \tau = 0$ and to say that the case/control status influences this CpG site. In other words, regarding $\mathbf{p}_i$ as the additive effect fails to detect this risk-CpG site in this case.

The main issue is as follows. Although the methylation difference between cases and controls

is as large as 1 in cell type 1, after mixing different cell types, the maximum methylation difference between cases and controls at the aggregated level attenuates to 0.2. The additive effect assumption fails to account for such an effect.

In comparison, if we incorporate $\mathbf{p}$ into the regression as multiplicative effects as in HIRE, we regress O_i on $x_i p_{1i}$, $x_i p_{2i}$ and p_{2i} for $i = 1, \ldots, 5$ with the intercept term:

$$O_i = \tilde{\alpha} + \beta_1 \cdot x_i p_{1i} + \beta_2 \cdot x_i p_{2i} + \mu p_{2i} + \epsilon_i. \tag{2}$$

β_1 and β_2 are the effects of the disease status on cell type 1 and cell type 2, respectively, and we want to test if they are zero or not. It turns out that the p-value for β_1 is 3.22×10^{-16} and the p-value for β_2 is 0.18. Thus, we are able to identify that the CpG site is at risk in cell type 1 but not in cell type 2, recovering the underlying truth. This successful identification is due to the multiplicative effect assumption acknowledging that the methylation difference from cell type 1 is modified by a factor of p_{1i} at the aggregated level. Consequently, HIRE rescues the risk-CpG site missed by the additive effect model.

Therefore, this example demonstrates that incorporating cellular compositions into the regression as multiplicative effects can not only detect the risk-CpG site, which might be missed by the additive effect regression, but can also enable cell-type-specific association detection.

Supplementary Methods

Let us assume that we have measured the methylation levels of m CpG sites for n samples. Throughout the paper, we refer to the methylation level as the beta-value, defined as the ratio of the methylated probe intensity and the overall probe intensity—the sum of the methylated and unmethylated probe intensities, which is between zero and one [1]. For a collection of cells containing different cell types, the observed beta-value is a weighted average of the cell-type-specific beta-values. Therefore, we focus on the beta-value rather than its transformation in our modeling as any nonlinear transformation can break the relationship between the aggregated-level measurement and the cell-type-specific methylation levels. Consequently, we use the "beta-value" and the "methylation level" exchangeably thereinafter.

We denote the observed methylation measurement of CpG site j in sample i by O_{ji}. We further assume that the collection of cells from sample i contain K cell types, and we represent the proportion of type k cells in sample i by p_{ki} $(1 \leq k \leq K)$. The baseline beta-value of CpG site j in cell type k is notated by μ_{jk}. Consequently, we have the following equation:

$$O_{ji} = \sum_{k=1}^{K} \mu_{jk} p_{ki} + \epsilon_{ji}, \tag{3}$$

where O_{ji} is the sum of the weighted average of cell-type-specific methylation levels $\{\mu_{jk} : k = 1, \ldots, K\}$ and ϵ_{ji} is the Gaussian-distributed measurement error with mean zero and standard deviation $\sigma_{\epsilon,j}$.

Equation (3) models the influence of cell-type heterogeneity on the methylation level, but it does not consider each sample's characteristics such as age, gender, and disease status. When there exist other sample attributes, Rahmani et al. [2] recommends first regressing O_{ji} on the covariates and then using the residuals to fit the model in Equation (3). However, the first stage regression tends to induce many putative associations between the methylation levels and the phenotype in the second stage. To jointly incorporate the phenotypes of interest and other known confounding factors, we replace μ_{jk} with u_{ijk} in Equation (3) and connect u_{ijk} to sample-specific covariates $\mathbf{x}_i = (x_{i1}, \ldots, x_{iq})^T$, which leads to the following HIRE model:

$$O_{ji} = \sum_{k=1}^{K} u_{ijk} p_{ki} + \epsilon_{ji}, \tag{4}$$

$$u_{ijk} \sim N(\mu_{jk} + \sum_{\ell=1}^{q} \beta_{jk\ell} x_{i\ell}, \sigma_{jk}^2). \tag{5}$$

In Equation (4), both u_{ijk} which depends on sample i's attributes and the cellular composition p_{ki} contribute to the methylation level O_{ji}. Equation (5) further assumes a linear model for u_{ijk} and covariates. The coefficient $\beta_{jk\ell}$ reflects the influence of attribute ℓ on CpG site j in cell type k. We can rewrite Equations (4) and (5) with matrices:

$$O_{ji} \sim N(\mathbf{u}_{ij}^T \mathbf{p}_i, \sigma_{\epsilon,j}^2), \tag{6}$$

$$\mathbf{u}_{ij} \sim N(\boldsymbol{\mu}_j + \mathbf{B}^{(j)} \mathbf{x}_i, \Sigma_j), \tag{7}$$

where $\mathbf{u}_{ij} = (u_{ij1}, \ldots, u_{ijK})^T$, $\mathbf{p}_i = (p_{1i}, \ldots, p_{Ki})^T$, $\boldsymbol{\mu}_j = (\mu_{j1}, \ldots, \mu_{jK})^T$, $\mathbf{B}^{(j)} = (\beta_{jk\ell})_{K \times q}$, and $\Sigma_j = diag(\sigma_{j1}^2, \ldots, \sigma_{jK}^2)$. The notation $diag(v_1, \ldots, v_p)$ represents a diagonal matrix with diagonals $v_1, \ldots, v_p$. Combining Equations (6) and (7) leads to

$$O_{ji} \sim N(\mathbf{p}_i^T \boldsymbol{\mu}_j + \mathbf{p}_i^T \mathbf{B}^{(j)} \mathbf{x}_i, \mathbf{p}_i^T \Sigma_j \mathbf{p}_i + \sigma_{\epsilon,j}^2). \tag{8}$$

Algorithm: GEM Algorithm for Finding MLE of HIRE Model (6) & (7).

Input: the observed methylation data $\mathbf{O}$, attributes $\mathbf{X}$, and tolerance δ.
Initialize: $\mathbf{\Theta}^{(0)}$, $t \leftarrow 0$.
Repeat
$t \leftarrow t+1$;
(E-step) calculate $Q(\mathbf{\Theta}|\mathbf{\Theta}^{(t-1)}) := E[l_c(\mathbf{\Theta}|\mathbf{O},\mathbf{u})|\mathbf{O},\mathbf{\Theta}^{(t-1)}]$;
(M-step) apply one-step coordinate ascent algorithm to $Q(\mathbf{\Theta}|\mathbf{\Theta}^{(t-1)})$
to obtain new estimates $\mathbf{\Theta}^{(t)}$.
Until $|l_o(\mathbf{\Theta}^{(t)}) - l_o(\mathbf{\Theta}^{(t-1)})| \leq \delta$.
Output:$\mathbf{\Theta}^{(t)}$.

Let $\mathbf{O} = \{O_{ji} : 1 \leq j \leq m, 1 \leq i \leq n\}$ denote the observed data and $\mathbf{\Theta} = \{\mathbf{p}_i, \boldsymbol{\mu}_j, \mathbf{B}_j, \Sigma_j, \sigma^2_{\epsilon,j} : 1 \leq j \leq m, 1 \leq i \leq n\}$ be the set of unknown parameters. The observed-data log-likelihood function, l_o, is

$$l_o(\mathbf{\Theta}|\mathbf{O}) = \sum_{i=1}^{n}\sum_{j=1}^{m} logN(O_{ji} : \mathbf{p}_i^T\boldsymbol{\mu}_j + \mathbf{p}_i^T\mathbf{B}^{(j)}\mathbf{x}_i, \mathbf{p}_i^T\Sigma_j\mathbf{p}_i + \sigma^2_{\epsilon,j}), \tag{9}$$

where $N(O_{ji} : z_1, z_2)$ represents the evaluation of the Gaussian density with mean z_1 and variance z_2 at point O_{ji}. The maximum likelihood estimates (MLE) of $\mathbf{\Theta}$ can be obtained by maximizing l_o with respect to $\mathbf{\Theta}$.

It is noteworthy that the direct maximization of l_o is intractable. Therefore, we employ the generalized expectation-maximization (GEM) algorithm [3]. When we augment the missing data $\mathbf{u} = \{\mathbf{u}_{ij} : 1 \leq i \leq n, 1 \leq j \leq m\}$ to the observed data $\mathbf{O}$, the complete-data log-likelihood function has a tractable form:

$$l_c(\mathbf{\Theta}|\mathbf{O},\mathbf{u}) = \sum_{i=1}^{n}\sum_{j=1}^{m}\{ -\frac{1}{2}log\sigma^2_{\epsilon,j} - \frac{(O_{ji} - \mathbf{u}_{ij}^T\mathbf{p}_i)^2}{2\sigma^2_{\epsilon,j}} - \frac{1}{2}\sum_{k=1}^{K} log\sigma^2_{jk} - \frac{1}{2}(\mathbf{u}_{ij} - \boldsymbol{\mu}_j - \mathbf{B}^{(j)}\mathbf{x}_i)^T\Sigma_j^{-1}(\mathbf{u}_{ij} - \boldsymbol{\mu}_j - \mathbf{B}^{(j)}\mathbf{x}_i)\} + Constant.$$

Accordingly, we can iterate between the E-step and the M-step. In the E-step, we take expectations for the complete-data log-likelihood function with respect to the missing data $\mathbf{u}_{ij}$ conditional on the observed data $\mathbf{O}$ and the estimates of the unknown parameters $\mathbf{\Theta}^{(t-1)}$ from the last iteration. In the M-step, we find new estimates $\mathbf{\Theta}^{(t)}$ which increases $E[l_c(\mathbf{\Theta}|\mathbf{O},\mathbf{u})|\mathbf{O},\mathbf{\Theta}^{(t-1)}]$ from its value in the last iteration. In fact, we can prove that $l_o(\mathbf{\Theta}^{(t)})$ increases after each iteration. We summarize the algorithm in the above box.

In the following, we elaborate the E-step and the M-step. In the E-step, the conditional density

of $\mathbf{u}_{ij}$ given O_{ji} and $\mathbf{\Theta}^{(t-1)}$ is

$$\begin{aligned} f(\mathbf{u}_{ij}|O_{ji},\mathbf{\Theta}^{(t)}) &\propto f(O_{ji}|\mathbf{u}_{ij},\mathbf{\Theta}^{(t)})f(\mathbf{u}_{ij}|\mathbf{\Theta}^{(t)}) \\ &\propto e^{-\frac{(O_{ji}-\mathbf{u}_{ij}^T\mathbf{p}_i^{(t)})^2}{2\sigma_{\epsilon,j}^{(t)2}}} e^{-\frac{1}{2}(\mathbf{u}_{ij}-\boldsymbol{\mu}_j^{(t)}-\mathbf{B}^{(j),(t)}\mathbf{x}_i)^T\Sigma_j^{(t)-1}(\mathbf{u}_{ij}-\boldsymbol{\mu}_j^{(t)}-\mathbf{B}^{(j),(t)}\mathbf{x}_i)} \\ &\propto e^{-\frac{1}{2}\left[\mathbf{u}_{ij}^T\left(\frac{\mathbf{p}_i^{(t)}\mathbf{p}_i^{(t)T}}{\sigma_{\epsilon,j}^{(t)2}}+\Sigma_j^{(t)-1}\right)\mathbf{u}_{ij}-2\left(\frac{O_{ji}\mathbf{P}_i^{(t)T}}{\sigma_{\epsilon,j}^{(t)2}}+(\boldsymbol{\mu}_j^{(t)}+\mathbf{B}^{(j),(t)}\mathbf{x}_i)^T\Sigma_j^{(t)-1}\right)\mathbf{u}_{ij}\right]}. \end{aligned}$$

Therefore, the conditional distribution of $\mathbf{u}_{ij}$ is $N(\boldsymbol{\mu}_{ij}^{(t)},\Sigma_{ij}^{(t)})$, where $\Sigma_{ij}^{(t)-1} := \frac{\mathbf{p}_i^{(t)}\mathbf{p}_i^{(t)T}}{\sigma_{\epsilon,j}^{(t)2}} + \Sigma_j^{(t)-1}$ and $\boldsymbol{\mu}_{ij}^{(t)T}\Sigma_{ij}^{(t)-1} := \frac{O_{ji}\mathbf{p}_i^{(t)T}}{\sigma_{\epsilon,j}^{(t)2}} + (\boldsymbol{\mu}_j^{(t)} + \mathbf{B}^{(j),(t)}\mathbf{x}_i)^T\Sigma_j^{(t)-1}$. In the matrix theory, when we invert a matrix with a form $A+B$ where A and $A+B$ are invertible and B has rank 1, we have $(A+B)^{-1} = A^{-1} - \frac{1}{1+g}A^{-1}BA^{-1}$ where $g = tr(BA^{-1})$. Therefore, $g = \frac{\sum_{k=1}^K \mathbf{p}_{ik}^{(t)2}\sigma_{jk}^{(t)2}}{\sigma_{\epsilon,j}^{(t)2}}$ and $\Sigma_{ij}^{(t)} = \Sigma_j^{(t)} - \frac{1}{1+g}\Sigma_j^{(t)}\frac{\mathbf{p}_i^{(t)}\mathbf{p}_i^{(t)T}}{\sigma_{\epsilon,j}^{(t)2}}\Sigma_j^{(t)}$, which does not involve any complicated matrix inversion. Subsequently, we can calculate:

$$\begin{aligned} &E\left[(O_{ji}-\mathbf{u}_{ij}^T\mathbf{p}_i)^2|O_{ji},\mathbf{\Theta}^{(t)}\right] = \mathbf{p}_i^T\Sigma_{ij}^{(t)}\mathbf{p}_i + (O_{ji}-\boldsymbol{\mu}_{ij}^{(t)T}\mathbf{p}_i)^2, \\ &E\left[(\mathbf{u}_{ij}-\boldsymbol{\mu}_j-\mathbf{B}^{(j)}\mathbf{x}_i)^T\Sigma_j^{-1}(\mathbf{u}_{ij}-\boldsymbol{\mu}_j-\mathbf{B}^{(j)}\mathbf{x}_i)|O_{ji},\mathbf{\Theta}^{(t)}\right] \\ &\quad = (\boldsymbol{\mu}_{ij}^{(t)}-\boldsymbol{\mu}_j-\mathbf{B}^{(j)}\mathbf{x}_i)^T\Sigma_j^{-1}(\boldsymbol{\mu}_{ij}^{(t)}-\boldsymbol{\mu}_j-\mathbf{B}^{(j)}\mathbf{x}_i) + \sum_{k=1}^K \frac{\sigma_{ij,kk}^{(t)2}}{\sigma_{jk}^2}, \end{aligned}$$

where $\sigma_{ij,kk}^{(t)2}$ is the kth diagonal element of matrix $\Sigma_{ij}^{(t)}$. In the M-step, we use the coordinate ascent algorithm to update each parameter one by one. The detailed procedures are as follows.

- Update $\mu_{jk}, \beta_{jk\ell}(\ell = 1,\ldots,q)$:

$$\begin{aligned} \mu_{jk}^{(t+1)} &= \frac{\sum_{i=1}^n(\mu_{ijk}^{(t)} - \sum_{\ell=1}^q \beta_{jk\ell}^{(t)}x_{i\ell})}{n}, \\ \beta_{jk\ell}^{(t+1)} &= \frac{\sum_{i=1}^n y_{ijk\ell}x_{i\ell}}{\sum_{i=1}^n x_{i\ell}^2}, \end{aligned}$$

  where $y_{ijk\ell} = \mu_{ijk}^{(t)} - \mu_{jk}^{(t+1)} - \sum_{s=1}^{\ell-1}\beta_{jks}^{(t+1)}x_{is} - \sum_{s=\ell+1}^q \beta_{jks}^{(t)}x_{is}$.

- Update $\mathbf{p}_i$:

$$\begin{aligned} &\min_{\mathbf{p}_i} \ \frac{1}{2}\mathbf{p}_i^T\left(2\sum_{j=1}^m \frac{\Sigma_{ij}^{(t)}+\boldsymbol{\mu}_{ij}^{(t)}\boldsymbol{\mu}_{ij}^{(t)T}}{\sigma_{\epsilon,j}^{2(t)}}\right)\mathbf{p}_i - \left(2\sum_{j=1}^m \frac{O_{ji}\boldsymbol{\mu}_{ij}^{(t)}}{\sigma_{\epsilon,j}^{(t)2}}\right)^T \mathbf{p}_i \\ &s.t. \ \ p_{ik} \geq 0, \quad \sum_{k=1}^K p_{ik} = 1. \end{aligned}$$

  This is a quadratic programming problem, which can be solved efficiently.

- Update $\sigma^2_{\epsilon,j}$:

$$\sigma^{(t+1)2}_{\epsilon,j} = \frac{1}{n}\left[\sum_{i=1}^{n} \mathbf{p}_i^{(t+1)}\Sigma_{ij}^{(t)}\mathbf{p}_i^{(t+1)} + \sum_{i=1}^{n}(O_{ji} - \boldsymbol{\mu}_{ij}^{(t)T}\mathbf{p}_i^{(t+1)})^2\right].$$

- Update σ^2_{jk}:

$$\sigma^{(t+1)2}_{jk} = \frac{1}{n}\left[\sum_{i=1}^{n}(\mu^{(t)}_{ijk} - \mu^{(t+1)}_{jk} - \sum_{\ell=1}^{q}\beta^{(t+1)}_{jk\ell}x_{i\ell})^2 + \sum_{i=1}^{n}\sigma^{(t)2}_{ij,kk}\right].$$

So far, we have assumed that the number of cell types K is known. In HIRE, we select K based on a variant of the penalized Bayesian information criterion ($pBIC$) criterion [4]. The $pBIC(\alpha)$, which depends on the family-wise error rate (FWER) control level α, is defined as follows,

$$pBIC(\alpha) := -2l_o(\hat{\mathbf{\Theta}}) + log(n)\cdot(d - d_0(\alpha)),$$

where $\hat{\mathbf{\Theta}}$ are the estimates from the algorithm, d is the number of parameters, and $d_0(\alpha)$ is the number of $\beta_{jk\ell}$s whose null hypotheses $H_0 : \beta_{jk\ell} = 0$ are not rejected at the FWER control level α. In detail, $d = (K-1)n + m(1+2K+qK)$ and $d_0(\alpha) = \#\{\beta_{jk\ell} : (p-value)_{jk\ell} > \alpha/(mKq)\}$. Throughout the paper, we set α to 0.01.

Supplementary References

[1] Pan Du, Xiao Zhang, Chiang-Ching Huang, Nadereh Jafari, Warren A Kibbe, Lifang Hou, and Simon M Lin. Comparison of Beta-value and M-value methods for quantifying methylation levels by microarray analysis. *BMC Bioinformatics*, 11(1):587, 2010.

[2] Elior Rahmani, Noah Zaitlen, Yael Baran, Celeste Eng, Donglei Hu, Joshua Galanter, Sam Oh, Esteban G Burchard, Eleazar Eskin, James Zou, et al. Sparse PCA corrects for cell type heterogeneity in epigenome-wide association studies. *Nature Methods*, 13(5):443–445, 2016.

[3] Arthur P Dempster, Nan M Laird, and Donald B Rubin. Maximum likelihood from incomplete data via the EM algorithm. *Journal of the Royal Statistical Society. Series B (Methodological)*, pages 1–38, 1977.

[4] Wei Pan and Xiaotong Shen. Penalized model-based clustering with application to variable selection. *Journal of Machine Learning Research*, 8(May):1145–1164, 2007.
