# Supplementary material for: Detection of cell-type-specific risk-CpG sites in epigenome-wide association studies
Source: Nat Commun. 2019 Jul 15;10:3113. doi: 10.1038/s41467-019-10864-z (PMC6629651; doi:10.1038/s41467-019-10864-z)
Supplement: Supplementary file 4 — Source Data [file 41467_2019_10864_MOESM4_ESM.zip › Source Data/Comparison R code with otherEWASmethods/src/FastLmmC.Manual.pdf]

User Manual

FaST-LMM

Factored Spectrally Transformed Linear Mixed Models

Version 2.07

Microsoft Research

Nov 1, 2013

Introduction

FaST-LMM, which stands for *Fa*ctored *S*pectrally *T*ransformed *L*inear *M*ixed *M*odels is a program for performing univariate genome-wide association studies (GWAS) on large data sets. It runs on both Windows and Linux systems, and has been tested on data sets with over 120,000 individuals.

This software is released under the Microsoft Research License Agreement, ("MSR-LA" or the "License"); you may not use the software except in compliance with the License. You can find a copy of the License in the file LICENSE.TXT accompanying this file. Links to updated versions can be found at: http://research.microsoft.com/en-us/um/redmond/projects/MSCompBio/fastlmm.

Versions of this software may also rely on additional libraries and code distributed under their respective licenses. To re-compile or build this code or derivatives thereof, you may be required to individually download and appropriately license some or all of these additional libraries for your specific use. See the file NOTICE.TXT, accompanying this file for more details.

For help with the software, please contact

Christoph Lippert, christoph.a.lippert@gmail.com
Jennifer Listgarten, jennl@microsoft.com
Carl Kadie, carlk@microsoft.com
Bob Davidson, bobd@microsoft.com
David Heckerman, heckerma@microsoft.com

Citing FaST-LMM

If you use FaST-LMM in any published work, please cite both the software (using the link http://research.microsoft.com/en-us/um/redmond/projects/MSCompBio/fastlmm) and the manuscript describing it:

C. Lippert, J. Listgarten, Y. Liu, C.M. Kadie, R.I. Davidson, and D. Heckerman. FaST Linear Mixed Models for Genome-Wide Association Studies. *Nature Methods* **8**: 833-835, Oct 2011 (doi:10.1038/nmeth.1681).

Also, we would appreciate it if you let us know that you are citing it.

Installing FaST-LMM

FaST-LMM is available as a .zip file that exacts to these directories:

`fastlmm/Bin`	contains the compiled executable files
`fastlmm/Cpp`	contains C++ source and project files
`fastlmm/Data/DemoData`	contains sample data and command script
`fastlmm/Doc`	contains project documentation
`fastlmm/Externals`	contains other code FaST-LMM depends on

There are executables for Windows (64bit), and for Ubuntu Linux (64bit) under the `fastlmm\Bin` directory and all required .dll files are included in the respective directories. These executables use the MKL math library, which is optimized for Intel processors but also runs on AMD processors. If one of these options is suitable, please skip ahead to section "Data Preparation" to see how to run FaST-LMM on your data. If not, please see the next section.

Compiling FaST-LMM[1]

In addition to the source code, the following external dependencies must be installed and met in order to build FaST-LMM:

Building for Windows

C++ of versions FaST-LMM are built with Visual Studio 2012 (VS).
Any version of VS (Express through Universal) is capable of building FaST-LMM. If you do not already have a copy of Visual Studio, the Visual Studio 2012 Express edition can be freely downloaded from
http://www.microsoft.com/express/downloads

- For the C++ version
  FaST-LMMC uses a 3rd party math library for advanced math functions and performance. `fastlmmc` can use either Intel's MKL or AMD's ACML math libraries. Once you have installed the appropriate library, use the Visual Studio IDE to select the appropriate configuration from the solution and build. ACML requires an additional step to tell Visual Studio where it is located. You must set the environment variable ACML_ROOT to point to your install location or libraries will not be located—for example,

  ```
  C>set ACML_ROOT=C:\AMD\acml4.4.0
  ```

  You can find more about the math libraries at their respective web sites:
  http://software.intel.com/en-us/articles/intel-mkl
  http://developer.amd.com/libraries/acml/pages/default.aspx

  With a math library installed, no additional libraries are required to compile the C++ version of FaST-LMM (`fastlmmc`). Double-click the `fastlmmc.sln` file to load Visual Studio and then build the solution associated with your library.

Building the C++ version of FaST-LMM for Linux

FaST-LMM is primarily developed and tested on Windows although we are able to build the C++ version for Linux. We provide a simple script file that uses the GNU toolset with the 3rd party math library to compile the sources in a Linux environment.

[1] When building the C++ version of FaST-LMM the build host machine name is captured and used in the program banner to help identify which version of the code is being run. If you build fastlmmc.exe and you do not want the machine name captured, you should modify the banner string in Splash.cpp to remove it .

- `fastlmmc` uses a 3rd party math library for advanced math functions and performance. The program has been run on Ubuntu Linux and can use either Intel's MKL or AMD's ACML math libraries for Linux. Once you have selected and installed the appropriate library, you can then build using the appropriate script file located in the `Cpp` directory. Review of the two files, `DoMKL_linux` and `DoAcml_linux`, will show very simple scripts to compile the program using g++ and then link the `.o` files with the appropriate math library. The `*.o` files are written to version specific directories, so it is necessary to create the appropriate directory prior to running the script. For more details, see the script.

  You can find more about the math libraries for Linux at their respective web sites:
  http://software.intel.com/en-us/articles/intel-mkl
  http://developer.amd.com/libraries/acml/pages/default.aspx

Data preparation

FaST-LMM uses four input files containing (1) the SNP data to be tested, (2) the SNP data used to determine the genetic similarities between individuals (which can be different from 1), (3) the phenotype data, and (4, optionally) a set of covariates.

When the realized relationship matrix (RRM) is used for genetic similarity, and when the number of SNPs used to construct the RRM is less than the number of individuals, the runtime and memory footprint of FaST-LMM scales linearly in the number of individuals in the data. When this condition is not met, the runtime and memory footprint of FaST-LMM are cubic and quadratic in the number of individuals, respectively.

All input files should be in ASCII.

Both SNP files (1 and 2 above) should be in PLINK format (`ped/map`, `tped/tfam`, `bed/bim/fam`, or `fam/dat/map`). For the most speed, use the binary format in SNP major order. The phenotype entries in these files must be set to some dummy value and will be ignored (our software uses a separate phenotype file). Sex should be encoded as a single digit. See the PLINK manual http://pngu.mgh.harvard.edu/~purcell/plink/[1] for further details. Missing SNP values will be mean imputed. Dosages files are also allowed (see the end of this section).

The required file containing the phenotype (3 above) uses the PLINK alternate phenotype format. It should have at least three columns: `<familyID>, <individualID>,` and any number of `<phenotype value>.` The columns are delimited by whitespace (<tab> or <space>). The default option is to test the first phenotype only. A missing value should be denoted by `-9`, but this can be changed (see options below). The first column, `<familyID>`, is joined with the second column `<individualID>` to create a unique key for the individual that matches an entry for an individual in the PLINK files above.

Example phenotype file for two phenotypes
(FaST-LMM/Data/DemoData/pheno.txt):

```
cid0P0      cid0P0      -0.183707553
cid1P0      cid1P0      0.648195255
cid2P0      cid2P0      -0.186926506
cid3P0      cid3P0      0.320979314
cid4P0      cid4P0      -0.550997213
...
```

Optionally, the phenotype file may also have a header row, for example, as follows:

```
FID   IID   MyPheno    YourPheno
```

The optional file containing covariates should have at least three columns: <familyID>, <individualID>, and any number of <covariate value>. The columns should be tab delimited. The token for missing values must be the same as that used in the phenotype file. All covariates are processed. Covariate files are nearly identical to phenotype files in form, but covariate files cannot have a header row.

Example covariate file (FaST-LMM/Data/DemoData/covariate.txt):

```
cid0P0      cid0P0      0.815745271
cid1P0      cid1P0      0.096974038
cid2P0      cid2P0      0.584075558
cid3P0      cid3P0      -0.288810245
cid4P0      cid4P0      -0.366616848
...
```

Instead of SNP data from which genetic similarities are computed, the user may provide the genetic similarities directly using the -sim <filename> option. The file containing the genetic similarities should be tab delimited and have both row and column labels for the IDs (family ID and individual ID separated by a space). The value in the top-left corner of the file should be var.

FaST-LMM supports dosage files in PLINK formats 1 and 2. The files may be uncompressed or compressed (with a .gz extension). Use -dfile1 or -dfile2 followed by the file prefix to load test SNPs in format 1 or 2, respectively. Use -dfile1Sim or -dfile2Sim followed by the file prefix to load similarity SNPs in format 1 or 2, respectively. As described in the PLINK manual, the .map file is optional and will be loaded if present.

Running FaST-LMM

Once you have prepared the files in the proper format, you can run FaST-LMM. Here is a sample call on the synthetic data provided in the `DemoData` folder:

```
C:\> fastlmmc -verboseOutput -bfile snps -bfilesim snps -pheno pheno.txt
-out snps.dummy.txt
```

You should see something similar to the following output on the screen:

```
FastLmmC v2.07.20131029 - Factored Spectrally Transformed Linear Mixed Models [Release]
  Copyright Microsoft Corporation -- Licensed Only for Non-Commercial use.
  Compiled Oct 29 2013 at 21:28:56 by ****** for Windows
  using MKL v11.00.04 - Build: 20130517

 ++    Start Processing CommandLine:
 --      End Processing CommandLine:

 ++      Start Loading FastLmm Data:
  ++  Start Loading Covariance Data:
   ++      Processing PLINK fileset: [snps]
   ReadBinaryFiles4()* elapsed time: 242.838 ms
     Number of Individuals Selected:    3000
               Number of Phenotypes:       1
                Number of SNPs Read:   10100
                Number of SNPs Used:   10100
   --  End Processing PLINK fileset: [snps]
  --     End Loading Covariance Data:
  ++         Start Loading Test Data:
   ++      Processing PLINK fileset: [snps]
   ReadBinaryFiles4()* elapsed time: 207.551 ms
     Number of Individuals Selected:    3000
               Number of Phenotypes:       1
                Number of SNPs Read:   10100
                Number of SNPs Used:   10100
   --  End Processing PLINK fileset: [snps]
  --           End Loading Test Data:

 --        End Loading FastLmm Data:

              Compute/Load EigenSym:
Warning : The kernel has a few Eigenvalues that are a tiny bit smaller
           than zero and are considered to be numerically zero.
Warning : Make the kernel positive semi definite.
Warning : Setting negative eigenvalues to zero.
             Compute GWAS using LMM:
                  GWAS elapsed time:  7.833 sec
                  Write output file: [snps.out.txt]
                 Total elapsed time: 16.042 sec
```

When the output file `[snps.dummy.txt]` is loaded in Excel, it should look as follows:

	A	B	C	D	E	F	G	H	I	J	K	L
1	SNP	Chromosome	GeneticDistance	Position	Phenotype	Pvalue	Qvalue	N	DOF	NullLogLike	AltLogLike	SNPWeight
2	snp26250_m0_.19m1_.19	1	15	15	Phenotype_001	1.20E-25	1.22E-21	3000	1	-2.41E+03	-2.35E+03	-1.06E-01
3	snp82500_m0_.28m1_.28	1	17	17	Phenotype_001	7.22E-20	3.64E-16	3000	1	-2.41E+03	-2.37E+03	-9.28E-02
4	snp63751_m0_.23m1_.23	1	63	63	Phenotype_001	3.57E-19	1.20E-15	3000	1	-2.41E+03	-2.37E+03	-9.09E-02
5	snp48753_m0_.4m1_.4	1	183	183	Phenotype_001	1.14E-14	2.88E-11	3000	1	-2.41E+03	-2.38E+03	-7.88E-02
6	snp45001_m0_.26m1_.26	1	71	71	Phenotype_001	3.98E-13	8.04E-10	3000	1	-2.41E+03	-2.38E+03	-7.42E-02
7	snp52500_m0_.05m1_.05	1	39	39	Phenotype_001	1.89E-09	3.19E-06	3000	1	-2.41E+03	-2.39E+03	-6.14E-02

	M	N	O	P	Q	R	S	T	U	V	W	X	Y
1	SNPWeightSE	OddsRatio	WaldStat	NullLogDelta	NullGeneticVar	NullResidualVar	NullBias	LogDelta	GeneticVar	ResidualVar	Bias	SNPIndex	SNPCount
2	1.01E-02	8.29E-01	1.12E+02	5.63E-01	1.07E-01	1.89E-01	-6.61E-02	6.82E-01	9.56E-02	1.89E-01	-6.61E-02	8	10100
3	1.01E-02	8.67E-01	8.44E+01	5.63E-01	1.07E-01	1.89E-01	-6.61E-02	6.92E-01	9.58E-02	1.91E-01	-6.61E-02	9	10100
4	1.01E-02	8.57E-01	8.12E+01	5.63E-01	1.07E-01	1.89E-01	-6.61E-02	6.67E-01	9.75E-02	1.90E-01	-6.61E-02	32	10100
5	1.02E-02	8.91E-01	6.02E+01	5.63E-01	1.07E-01	1.89E-01	-6.61E-02	6.53E-01	9.92E-02	1.91E-01	-6.61E-02	92	10100
6	1.02E-02	8.87E-01	5.31E+01	5.63E-01	1.07E-01	1.89E-01	-6.61E-02	5.63E-01	1.06E-01	1.85E-01	-6.61E-02	36	10100
7	1.02E-02	8.15E-01	3.63E+01	5.63E-01	1.07E-01	1.89E-01	-6.61E-02	6.23E-01	1.02E-01	1.90E-01	-6.61E-02	20	10100

The standard and `-verboseOut` columns are:

`SNP`

The rs# or SNP identifier for the SNP tested. Taken from the PLINK file.

`Chromosome`

The chromosome identifier for the SNP tested or 0 if unplaced. Taken from the PLINK file.

`Genetic Distance`

The location of the SNP on the chromosome. Taken from the PLINK file. Any units are allowed, but typically centimorgans or morgans are used.

`Position`

The base-pair position of the SNP on the chromosome (bp units). Taken from the PLINK file.

`Phenotype [under -verboseOut]`

The name of the phenotype as specified in the header of the phenotype file. `NoName` means that no header row was specified.

`Pvalue`

The p-value computed for the SNP tested

`Qvalue`

The *q*-value computed for the SNP tested estimated from the *p*-values of all test-SNPs in the PLINK file using the procedure of Benjamini and Hochberg

`N`

The sample size or number of individuals that have a been used for this analysis

`NumSNPsExcluded [under -excludeByGeneticDistance]`

`IndexExclusionStart [under -excludeByGeneticDistance]`

`DOF [under -verboseOut]`

The degrees of freedom of the statistical test

`NullLogLike`

The log likelihood of the null model

`AltLogLike`

The log likelihood of the alternative model

`SnpWeight`

The fixed-effect weight of the SNP

`SnpWeightSE`

The standard error of the SnpWeight

`OddsRatio`

The odds ratio of the SNP

`WaldStat`

The Wald statistic

`NullLogDelta`

The ratio between the residual variance and the genetic variance $\delta = \sigma_e^2/\sigma_g^2$ on the null model

`NullGeneticVar`

The genetic variance σ_g^2 on the null model

`NullResidualVar`

The residual variance σ_e^2 on the alternative model

`NullBias`

The offset term in the null model

`LogDelta [under -verboseOut]`

The ratio between the residual variance and the genetic variance $\delta = \sigma_e^2/\sigma_g^2$ on the alternative model

`geneticVar [under -verboseOut]`

The genetic variance σ_g^2 on the alternative model

`ResidualVar [under -verboseOut]`

The residual variance σ_e^2 on the alternative model

`Bias [under -verboseOut]`

The offset term in the alternative model

`SNPIndex`

The column index of the SNP tested in the PLINK file starting at 1

`SNPCount`

The number of SNPs tested

SNP standardization

By default, each SNP is standardized to have mean zero and standard deviation one across all individuals. Another standardization method that can be used is to scale SNP values by multiplying by the beta(MAF,a,b) probability density function. This method is called using the `beta` flag, which takes a and b as parameters. If a and b are not specified, then a and b are set to 1 and 25, respectively, as recommended in ref[2]. If one parameter is specified, then both parameters must be specified.

Avoiding proximal contamination

To understand proximal contamination, first note that a LMM with no fixed effects, using a realized relationship matrix (RRM) for genetic similarities, is mathematically equivalent to linear regression of the SNPs on the phenotype, with weights integrated over independent Normal distributions having the same variance.[3] That is, a LMM using a given set of SNPs for genetic similarity is equivalent to a form of linear regression using those SNPs as covariates to correct for confounding. This equivalence implies that, when testing a given SNP, that SNP (and SNPs physically close to it) should be excluded from the computation of genetic similarity. If not, when testing a particular SNP, we would also be using that same SNP as a covariate, making the log likelihood of the null model higher than it should be, thus leading to deflation of the test statistic and loss of power. We call this phenomenon *proximal contamination.*[4,5]

To combat proximal contamination, we need to exclude the SNP we are testing from the genetic similarity matrix and also those SNPs in close proximity to it. Doing so in a naïve way, however, is extremely computationally expensive. FaST-LMM, however, can

do this exclusion efficiently.[5] To use this feature, either genetic distances or positions must be included in the PLINK files specifying both the test SNPs and the SNPs used for genetic similarity. Furthermore, the SNPs must be in non-decreasing order according to chromosome and the distance used. To perform the exclusion, use either the option `-excludeByGeneticDistance` or `-excludeByPosition` followed by a parameter describing the distance (centimorgans or nucleotide position). In practice, we have found the values 2 centimorgans or 2 million positions to work well. The units of the parameters for these flags are the same as the units provided in the SNP files (which is user-determined). That is, if the SNP genetic distances are specified in Morgans, for example, then the option `-excludeByGeneticDistance 0.02` will exclude SNPs within 0.02 Morgans.

FaST-LMM-Select: SNP selection

Note: an unpublished improved method, implemented in Python, is available on the FaST-LMM download page (see FaST-LMM-Select), and we recommend using this approach for SNP selection. We leave this section and associated functionality here for reproducibility of results in our older papers, but no longer advocate using it.

The equivalence between LMMs and linear regression also suggests another improvement. Regardless of what form of regression we use for GWAS, the measure of SNP-phenotype association (*e.g.*, a *P* value) should be determined by conditioning on exactly those SNPs that are associated with the phenotype. These SNPs include causal SNPs or SNPs that tag causal SNPs, and SNPs that are associated by way of confounding (*e.g.*, population structure). By conditioning on causal or tagging SNPs, we reduce the noise in the assessment of the association.[6] By conditioning on SNPs associated by virtue of confounding, we control for such confounding.[7,8] Moreover, if a SNP is unrelated to the phenotype, it should not be in the conditioning set. In the particular case where we are using linear regression for GWAS, the inclusion in the genetic similarity matrix of SNPs that are unrelated to the phenotype or the exclusion of related SNPs leads to model misspecification, which in turn leads to inflated test statistics and reduced power.

To identify those SNPs which should be used for genetic similarity as just specified, the following approach involving cross validation has successfully been used. First, partition the data across individuals into k blocks (*e.g.*, k=10). This partitioning defines k folds for cross validation, where the ith fold has test data equal to the ith block and training data equal to the remaining data. For each fold, use the training data to order SNPs by their linear-regression *P* values in increasing order. Then, again on the training data, construct genetic similarity matrices with an increasing number of SNPs according to this ordering, and use the resulting model to predict the test data (note, such predictions are done via a Gaussian process corresponding to the mixed model). Next, identify the number m of SNPs that maximizes the out-of-sample log likelihood (or minimizes the mean-squared error) summed over all folds. Finally, use the full data to order SNPs by their linear-regression *P* values in increasing order, and select the first m SNPs. *Note that, when performing variable selection, it is especially important to avoid proximal contamination.*

This procedure is called by using the `-autoSelect` option. The call outputs the select SNPs to a file, which can then be used in a subsequent call to FaST-LMM to perform GWAS using the `-extractSim` option. Here is an example of calls to perform selection followed by GWAS:

```
> fastlmmc -autoselect ASout -autoSelectSearchValues "0 1 2 4 8 16 32 64 128
256 512 1024 2048 10100" -randomSeed 1 -autoselectFolds 10 -bfilesim snps -
pheno pheno.txt -covar covariate.txt

> fastlmmc -bfile snps -bfilesim snps -extractSim ASout.snps.txt -pheno
pheno.txt -covar covariate.txt -out snps.insample.txt -excludebyPosition 1
```

In the first call, `ASout` is the file prefix for the output of AutoSelect, consisting of a list of snps (`ASout.snps.txt`) and the statistics of the search (`ASout.xval.txt`). The option `-autoSelectSearchValues` is optional and requires a white-space delimited list of number-of-SNPs to try in the search. This list can be included in double quotes on the command line as above, or the list can be in a separate file specified on the command line. We recommend that you first try 1, 2, 4, …, 2024, and all SNPs. Note that you will typically see two local maxima in prediction accuracy, one at a relatively small number of SNPs, and one at all SNPs. For the maxima corresponding to a relatively small number of SNPs, we recommend that you refine the search once you identify the rough location of the peak. If the two maxima yield similar prediction accuracies, you could perform GWAS using each set of SNPs and select the analysis that yields the most conservative (i.e., smallest) genomic control factor, λ.

In the second call, `-extractSim` is used to select the SNPs listed in `ASout.snps` from the full set of SNPs in `snps`.

Speed vs. accuracy considerations

Please ignore these comments if you are using the new selection method in Python.

The FaST-LMM analysis involves a search over the ratio δ of genetic and environmental variances. As this step represents a non-convex optimization FaST-LMM performs an optimization procedure over several intervals on a logarithmic scale, invoking iterative calls to the likelihood function. The total run-time of this step scales linear in the sample size times a constant that approximately equals the number of intervals considered for the search.

The command line option `-simLearnType Full` is set by default to perform “exact” LMM inference that avoids this potential loss of power by refitting the ratio δ of variances for every SNP tested.

Use the command line option `-simLearnType Once` to gain a constant factor speedup. Using this option, the ratio δ is found on the null-model only and is fixed to that value throughout the testing procedure. Note, though, that on some data sets this could lead to slight loss of power when SNPs with a large effect are tested.

Additionally, the number and coarseness of the search intervals can be adjusted via the command line options `-brentStarts <int>` for the number of intervals,

`-brentMinLogVal <double>` for the minimum of the search scope of log-δ values, and
`-brentMaxLogVal <double>`, for the maximum of the search scope of log-δ values.

By default the search is set conservatively to span 100 intervals over δ values between $\ln(-5)$ and $\ln(5)$.

Epistasis

FaST-LMM supports the analysis of epistatic interactions for pairs of SNPs[9]. Interaction analysis is activated through use of the `-snpPairs` option. Data preparation for a SNP pair run is the same as for univariate analysis.

An example analysis is called as follows:

```
> fastlmmc -snpPairs -bfile snps -bfileSim snps -extractSim ASout.snps.txt -
pheno pheno.txt -covar covariate.txt -out snps.pairs.txt
```

This call will produce an analysis of each SNP pair in `-bfile`. Note that proximal contamination is not a concern when running epistatic runs.

Pairwise analysis of *m* SNPs in `-bfile` will perform *m *(m -1)/2* tests, which can lead to extremely large output. In this case, with *m* of 10100, the number of pairs tested is 50,999,950. FaST-LMM has the ability to restrict the analysis to a subset of the SNPs in your data. This is done with the `-extract` option:

```
> fastlmmc -snpPairs -bfile snps -extract snps.subset.txt -bfileSim snps -
extractSim ASout.snps.txt -pheno pheno.txt -covar covariate.txt -out
snps.subset.pairs.txt
```

If you want to explore the whole space, FaST-LMM has the ability to partition the work into subtasks making it easy to run a parametric sweep on a cluster and merge the results. The `-task` option takes two integer parameters `number_of_tasks` and `current_task` as follows:

```
> fastlmmc -snpPairs -bfile snps -bfileSim snps -extractSim ASout.snps.txt -
pheno pheno.txt -covar covariate.txt -out snps.pairs.txt -tasks 250 0
```

Note that `fastlmmc` creates tasks of near identical size to spread the work evenly and perform the *m*(m-1)/2* tests. However, it is not possible to guarantee the work divides evenly into the task count. In this case (where tasks=250 and *m*=10100), `fastlmmc` divides the 10100 SNPs into blocks of 480 or 481 and the work will be completed by task 231. You can still enter tasks from 232 to 249 and they will produce a valid output file with 0 SNP pairs in the file.

The output columns are as follows:

`snpID_1`
: Unique SNP identifier for first SNP

`snpID_2`
: Unique SNP identifier for second SNP

`pvalue`
: The p-value computed for the SNP pair tested

`SnpPair_Wgt`
: The fixed-effect weight of the SNP pair tested

`SnpPair_OddsRatio`
: The odds ratio of the SNP pair tested

Command line options

`-file basefilename`
: basename for PLINK's `.map` and `.ped` files

`-bfile basefilename`
: basename for PLINK's binary `.bed`, `.fam`, and `.bin` files

`-tfile basefilename`
: basename for PLINK's transposed `.tfam` and `.tped` files

`-dfile1 basefilename`
: basename for PLINK's `.dat`, `.fam`, and (optionally) `.map` files, format=1

`-dfile2 basefilename`
: basename for PLINK's `.dat`, `.fam`, and (optionally) `.map` files, format=2

`-noDosageRangeCheck`
: disables range checking for dosage files. Default: `false`.

`-beta <a b>`
: scales values for each SNP across individuals by dividing by beta(a,b). Default values for a and b are 1. If either is specified, both must be specified.

`-pheno filename`
: name of phenotype file

`-mpheno index`
: index for phenotype in -pheno file to process, starting at 1 for the first phenotype column. Cannot be used together with `-pheno-name`. Default: 1.

`-pheno-name name`
: phenotype name for phenotype in `-pheno` file to process. If this option is used, the phenotype name must be specified in the header row. Cannot be used together with `-mpheno`.

`-fileSim basefilename`
: basename for PLINK's `.map` and `.ped` files for computing genetic similarity

`-bfileSim basefilename`
: basename for PLINK's binary `.bed`, `.fam`, and `.bin` files for building genetic similarity

`-tfileSim basefilename`
: basename for PLINK's transposed `.tfam` and `.tped` files for building genetic similarity

`-dfile1Sim basefilename`
: basename for PLINK's `.dat`, `.fam`, and (optionally) `.map` files for building genetic similarity, format=1

`-dfile2Sim basefilename`
: basename for PLINK's `.dat`, `.fam`, and (optionally) `.map` files for building genetic similarity, format=2

`-sim filename`
: specifies that genetic similarities are to be read directly from this file

`-simOut filename`

specifies that genetic similarities are to be written to this file

`-linreg`

specifies that linear regression will be performed. When this option is used, no genetic similarities should be specified.

`-logreg`

specifies that logistic regression will be performed. When this option is used, no genetic similarities should be specified.

`-covar filename`

optional file containing the covariates

`-missingPhenotype <dbl>`

identifier for missing values. If the phenotype for an individual is missing, then the individual is ignored. If a covariate value for an individual is missing, then it is mean imputed. Default: `-9`.

`-out filename`

the name of the output file. Default value is `[basefilename].out.txt`. If the extension `.csv` is used, then the output is comma separated. Otherwise, the output is tab separated.

`-simLearnType [Full/Once]`

if set to `Once`, then delta, the ratio of residual to genetic covariance, is optimized only for the null model and used for each alternate model. If set to `Full` (the default), then the ratio is re-estimated for each alternative model.

`-simType [RRM/COVARIANCE]`

if set to `RRM` (the default), then the RRM is used for genetic similarity. If set to `COVARIANCE`, then the empirical SNP covariance matrix is used.

`-ML`

use maximum likelihood parameter learning (default is `REML`)

`-REML`

use restricted maximum likelihood parameter learning. `REML` will automatically invoke the F-test.

`-Ftest`

use F-test (with `ML` or `REML`).

`-brentStarts <int>`

number of interval boundary points for optimization of delta (see Section 2.1 of the Supplemental Information). Default: `100`.

`-brentMaxIter <int>`

maximum number of iterations per interval for the optimization of delta. Default: `1e5`.

`-brentMinLogVal <double>`

lower interval threshold for (log) delta optimization. Default: `-5`.

`-brentMaxLogVal <double>`

upper interval threshold for (log) delta optimization. Default: `5`.

-brentTol <double>

convergence tolerance of Brent's method used to optimize delta. Default: 1e-6.

-runGwasType [RUN/NORUN]

run GWAS or exit after computing the spectral decomposition of the genetic similarity matrix. Use NORUN, to cache the spectral decomposition. This option, in combination with the next, is useful for parallelizing the tests of many SNPs. Default: RUN.

-eigen [directoryname]

load the spectral decomposition object from the directory name. The computations leading to the spectral decomposition of the genetic similarity matrix are skipped (note that that SNP file specifying the genetic similarities must still be given).

-eigenOut [directoryname]

save the spectral decomposition object to the directory name. Can be used with −runGwasType option.

-numJobs <int>

partition the SNPS into <int> groups and run FaST-LMM on the partition specified by -thisjob.

-thisJob <int>

specifies which partition of SNPS created by -numjobs to process for this run of FaST-LMM.

-extract filename

this is a SNP filter option. FaST-LMM will only analyze the SNPs explicitly listed in the 'filename' (no header, one SNP per line, where the SNP is indicated by the rs# or snp identifier).

-extractSim filename

this is a genetic similarity SNP filter option. FaST-LMM will only use SNPs explicitly listed in the 'filename' for computing genetic similarity.

-extractSimTopK filename <int>

similar to –extractSim, this is a genetic similarity SNP filter option. FaST-LMM will only use the first <int> SNPs explicitly listed in the 'filename' for computing genetic similarity.

-verboseOut

enable a more detailed and verbose output file with more columns. (See output)

-setOutputPrecision <int>

FastLmmC uses doubles for computation and has a default output precision of 16 digits after the decimal point. When working with large numbers of SNPs, writing full 16 digit precision can produce output that is quite large and this output precision may not be necessary. You can reduce the digits written in the output and reduce the file size using the -SetOutputPrecision <int> option. The parameter is restricted to the range 3 <= <int> <= 18.

`-pValuePrintThreshold <dbl>`

this option sets a threshold filter to restrict the report output to include only those SNPs that have a p-value less than the specified value `<dbl>`. When large datasets are used, PvaluePrintThreshold produces smaller and more manageable output files.

The parameter is restricted to the range `0.0 < <dbl> <= 1.0`.

`-maxThreads <int>`

the option is passed to the MKL math libraries to ‘suggest’ the level of parallelism to use. Assigning a number larger than the number of cores on your machine may cause the program to run slower. Assigning a number less than the number of cores on your machine may allow your computer to run FastLmmC without consuming all the CPU resources in different phases of the program.

The MaxThreads option is currently ignored when using ACML math libraries.

`-excludeByGeneticDistance <dbl>`

excludes the SNP tested and those within this distance from the genetic similarity matrix. To use this feature genetic distances must be included in the PLINK files specifying both the test SNPs and the SNPs used for genetic similarity. The SNPs must be in non-decreasing order according to chromosome and the distance used.

`-excludeByPosition <int>`

excludes the SNP tested and those within this distance from the genetic similarity matrix. To use this feature positions must be included in the PLINK files specifying both the test SNPs and the SNPs used for genetic similarity. The SNPs must be in non-decreasing order according to chromosome and the distance used.

`-autoSelect filename`

determines the SNPs to include in the similarity matrix (see description in main text). When this option is used, GWAS is not run. SNPs are written to `filename.snps.txt`, and statistics of the SNP search are written to `filename.xval.txt`

`-autoSelectSearchValues <int_list>|<filename>`

when running AutoSelect, this specifies which number-of-SNPs to use for the search. The <argument> is either a “list-of-integers” on the command line or the name of a file containing a “list-of-integers.” A “list-of-integers” is a string of integers separated by a whitespace or comma. If you use a list on the command line, it should be enclosed in double quotes. The default values are “0 1 2 3 4 5 6 7 8 9 10 20 30 40 50 60 70 80 90 100 125 160 200 250 320 400 500 630 800 1000”.

`-autoSelectCriterionLL`

directs AutoSelect to use out-of-sample log likelihood for the selection criterion (default).

`-autoSelectCriterionMSE`

directs AutoSelect to use out-of-sample mean-squared error for the selection criterion.

`-autoSelectFolds <int>`

specifies the number of folds to be used in AutoSelect. The default is 10.

`-randomSeed <int>`

specifies the random seed used in AutoSelect.

`-topKbyLinReg <int>`

directs AutoSelect to use only the top `<int>` SNPs, as determined by linear regression, while selecting SNPs.

`-memoryFraction <dbl>`

specifies the fraction of memory to use when invoking `-topKByLinReg`. Default is 0.2.

`-snpPairs`

perform GWAS using SNP pairs rather than individual SNPs. SnpPairs forces the use of the -ML option.

`-tasks <int1> <int2>`

breaks the total work into multiple tasks and runs one. The first argument sets the number of tasks to create and the second number the task to run. The task index is zero based. There will likely be 'empty' files produced by the last few tasks.

References

1. Purcell, S. *et al.* PLINK: a tool set for whole-genome association and population-based linkage analyses. *American Journal of Human Genetics* **81,** 559–75 (2007).

2. Wu, M. C. *et al.* Rare-variant association testing for sequencing data with the sequence kernel association test. *American journal of human genetics* **89,** 82–93 (2011).

3. Hayes, B. J., Visscher, P. M. & Goddard, M. E. Increased accuracy of artificial selection by using the realized relationship matrix. 47–60 (2009). doi:10.1017/S0016672308009981

4. Lippert, C. *et al.* FaST linear mixed models for genome-wide association studies. *Nature methods* **8,** 833–5 (2011).

5. Listgarten, J. *et al.* Improved linear mixed models for genome-wide association studies. *Nature methods* **9,** 525–6 (2012).

6. Hoggart, C. J., Whittaker, J. C., De Iorio, M. & Balding, D. J. Simultaneous analysis of all SNPs in genome-wide and re-sequencing association studies. *PLoS genetics* **4,** e1000130 (2008).

7. Setakis, E., Stirnadel, H. & Balding, D. J. Logistic regression protects against population structure in genetic association studies Logistic regression protects against population structure in genetic association studies. *Genome Research* 290–296 (2006). doi:10.1101/gr.4346306

8. Listgarten, J., Lippert, C. & Heckerman, D. FaST-LMM-Select for addressing confounding from spatial structure and rare variants. *Nature Genetics* **In press,** (2013).

9. Lippert, C. *et al.* An exhaustive epistatic SNP association analysis on expanded Wellcome Trust data. *Scientific reports* **3,** 1099 (2013).

Revision History

Date	Author(s)	Description of Changes
12/2/2011	Heckerman<br>Davidson	Update for v1.04<br>add -dosage support
3/13/2012		Update for v1.08<br>document new output formats option -`verboseOut`<br>document new similarity option -`extractSim`
6/1/2012		Update for v2.00<br>license and numerous other changes<br>Add warning about hostname usage during build process
8/31/2012		Update for V2.01<br>various bug fixes<br>document new output formats option -`SetOutputPrecision`
10/1/2012		Update for v2.02<br>various bug fixes<br>fix -`LinReg` output<br>document new -`PvaluePrintThreshold` option
10/25/2012		Update for v2.03<br>various bug fixes<br>document -`AutoSelect` options<br>gzip support of dosage files
2/21/2013		Update for v2.04<br>various bug fixes<br>document -`LogReg` option
3/28/2013		Update for v2.05<br>various bug fixes<br>add documentation for Epistatic analysis
7/12/2013		Update for v2.06<br>various bug fixes<br>Update VisualStudio compiler and MKL
11/1/2013		Update for v2.07<br>various bug fixes<br>separate build so fastlmmc can run on RHEL w/ v2.x kernel & tools<br>changes to autoselect<br>added -beta option
