# Supplementary material for: Detection of cell-type-specific risk-CpG sites in epigenome-wide association studies
Source: Nat Commun. 2019 Jul 15;10:3113. doi: 10.1038/s41467-019-10864-z (PMC6629651; doi:10.1038/s41467-019-10864-z)
Supplement: Supplementary file 4 — Source Data [file 41467_2019_10864_MOESM4_ESM.zip › Source Data/Rpackage HIREewas/HIREewas_1.2.0.tar.gz!/HIREewas/inst/doc/HIREewas.pdf]

HIREewas: Detection of Cell-Type-Specific Risk-CpG Sites in EWAS User's Guide

***Xiangyu Luo*[*], *Can Yang* [†], *and Yingying Wei* [‡]**

[*]xyluo1991@gmail.com The Chinese University of Hong Kong [†]The Hong Kong University of Science and Technology [‡]The Chinese University of Hong Kong

May 2, 2019

Contents

1 Introduction 2

2 Data Preparation 3

3 Model Application 7

4 Visualization 15

HIREewas: Detection of Cell-Type-Specific Risk-CpG Sites in EWAS User's Guide

1 Introduction

In epigenome-wide association studies (EWAS), as samples are measured at the bulk level rather than at the single-cell level, the obtained methylome for each sample shows the signals aggregated from distinct cell types [1, 2, 3]. The cellular heterogeneity leads to two main challenges for analyzing EWAS data.

On the one hand, the cell type compositions differ between samples and can be associated with phenotypes [1, 3]. Both binary phenotypes, such as the diseased or normal status [1], and continuous phenotypes, for example, age [3], have been found to affect the cell type compositions. As a result, ignoring the cellular heterogeneity in EWAS can lead to a large number of spurious associations [3, 4, 5, 6]. On the other hand, the phenotype may change the methylation level of a CpG site in some but not all of the cell types. Identifying the exact cell types that carry the risk-CpG sites can deepen our understandings of disease mechanisms. Nevertheless, such identification is challenging because we can only observe the aggregated-level signals.

However, to the best of our knowledge, no existing statistical method for EWAS can detect cell-type-specific associations despite the active research on accounting for cell-type heterogeneity. The existing approaches can be categorized into two schools [7]: "reference-based" and "reference-free" methods. As the method names indicate, the reference-based methods [2, 8] require the reference methylation profiles for each cell type to be known a priori, while the reference-free methods do not depend on any known methylation reference by employing matrix decomposition techniques [9] or extracting surrogate variables including principle components as a special case [10, 11, 6, 4].

Although all of the existing methods aim to address the cellular heterogeneity problem in EWAS and claim whether a CpG site is associated with phenotypes at the *aggregated level*, none of them can identify the risk-CpG sites for each *individual cell type*, thus missing the opportunity to obtain finer-grained results in EWAS.

We propose a hierarchical model HIRE [**?**] to identify the association in EWAS at an unprecedented HIgh REsolution: detecting whether a CpG site has any associations with the phenotypes in each cell type. HIRE not only substantially improves the power of association detection at the aggregated level as compared to the existing methods but also enables the

detection of risk-CpG sites for individual cell types. HIRE is applicable to EWAS with binary phenotypes, continuous phenotypes, or both.

The user's guide provides step-by-step instructions for the *HIREewas* R package. We believe that, by helping biology researchers understand in which cell types the CpG sites are affected by a disease using *HIREewas*, HIRE can ultimately facilitate the development of epigenetic therapies by targeting the specifically affected cell types.

2 Data Preparation

We first introduce the input data format. The input data consists of the methylation values and the covariates. The methylation values should be organized into a matrix `Ometh`, where each row represents a CpG site and each column corresponds to a sample. In other words, the (i, j) element of `Ometh` is the methylation value for sample j in CpG site j. The covariate data are also arranged in a matrix `X`. Each row of `X` denotes one covariate, so the row number is equal to the number of covariate types. Columns of `X` represent samples. Therefore, the (ℓ, j) element of `X` is the covariate ℓ information of sample j.

For demonstration, we then generate a dataset following the simulation steps in [**?**].

```
################################################################
#Generate the EWAS data
################################################################

set.seed(123)
###define a function to draw samples from a Dirichlet distribution
rDirichlet <- function(alpha_vec){
        num <- length(alpha_vec)
        temp <- rgamma(num, shape = alpha_vec, rate = 1)
        return(temp / sum(temp))
}

n <- 180     #number of samples
n1 <- 60    #number of controls
```

```
n2 <- 120    #number of cases

m <- 2000   #number of CpG sites
K <- 3       #underlying cell type number

###simulate methylation baseline profiles
#assume cell type 1 and cell type 2 are from the same lineage
#cell type 1
methy1 <- rbeta(m,3,6)
#cell type 2
methy2 <- methy1 + rnorm(m, sd=0.01)
ind <- sample(1:m, m/5)
methy2[ind] <- rbeta(length(ind),3,6)

#cell type 3
methy3 <- rbeta(m,3,6)
mu <- cbind(methy1, methy2, methy3)

#number of covariates
p <- 2

###simulate covariates / phenotype (disease status and age)
X <- rbind(c(rep(0, n1),rep(1, n2)), runif(n, min=20, max=50))

###simulate phenotype effects
beta <- array(0, dim=c(m,K,p))

#control vs case
m_common <- 10
max_signal <- 0.15
min_signal <- 0.07

#we allow different signs and magnitudes
signs <- sample(c(-1,1), m_common*K, replace=TRUE)
beta[1:m_common,1:K,1] <- signs * runif(m_common*K, min=min_signal, max=max_signal)

m_seperate <- 10
signs <- sample(c(-1,1), m_seperate*2, replace=TRUE)
```

```
    beta[m_common+(1:m_seperate),1:2,1] <- signs *
                                            runif(m_seperate*2, min=min_signal, max=max_signal)

    signs <- sample(c(-1,1), m_seperate, replace=TRUE)
    beta[m_common+m_seperate+(1:m_seperate),K,1] <- signs *
                                            runif(m_seperate, min=min_signal, max=max_signal)

    #age
    base <- 20
    m_common <- 10
    max_signal <- 0.015
    min_signal <- 0.007
    signs <- sample(c(-1,1), m_common*K, replace=TRUE)
    beta[base+1:m_common,1:K,2] <- signs *
                                            runif(m_common*K, min=min_signal, max=max_signal)

    m_seperate <- 10
    signs <- sample(c(-1,1), m_seperate*2, replace=TRUE)
    beta[base+m_common+(1:m_seperate),1:2,2] <- signs *
                                            runif(m_seperate*2, min=min_signal, max=max_signal)

    signs <- sample(c(-1,1), m_seperate, replace=TRUE)
    beta[base+m_common+m_seperate+(1:m_seperate),K,2] <- signs *
                                            runif(m_seperate, min=min_signal, max=max_signal)

    ###generate the cellular compositions
    P <- sapply(1:n, function(i){
                                    if(X[1,i]==0){ #if control
                                            rDirichlet(c(4,4, 2+X[2,i]/10))
                                    }else{
                                            rDirichlet(c(4,4, 5+X[2,i]/10))
                                    }
                            })

    ###generate the observed methylation profiles
    Ometh <- NULL
    for(i in 1:n){
            utmp <- t(sapply(1:m, function(j){
```

```
                                        tmp1 <- colSums(X[ ,i] * t(beta[j, , ]))
                                        rnorm(K,mean=mu[j, ]+tmp1,sd=0.01)
                                })) 
        tmp2 <- colSums(P[ ,i] * t(utmp))
        Ometh <- cbind(Ometh, tmp2 + rnorm(m, sd = 0.01))
}

#constrain methylation values between 0 and 1
Ometh[Ometh > 1] <- 1

Ometh[Ometh < 0] <- 0
```

Here we simulated a methylation matrix `Ometh` with 2,000 CpG sites and 180 samples as well as a covariate matrix `X` with one binary covariate (case/control) in the first row and one continuous variable (age) in the second row. We can further look into the details.

```
#the class of the methylation matrix
class(Ometh)

## [1] "matrix"

#the values in the methylation matrix
head(Ometh[,1:6])

##           [,1]      [,2]      [,3]      [,4]      [,5]      [,6]
## [1,] 0.3202140 0.3440603 0.3619823 0.2854539 0.3861594 0.3808383
## [2,] 0.2839342 0.3131437 0.3168782 0.3031998 0.3207638 0.3062112
## [3,] 0.5767125 0.5339113 0.5143695 0.6126050 0.5041764 0.5092417
## [4,] 0.3597017 0.2931941 0.3259443 0.3294055 0.3200657 0.2760185
## [5,] 0.3632531 0.3303706 0.3678126 0.3569870 0.3607428 0.3345626
## [6,] 0.3645631 0.3281156 0.3308156 0.4126270 0.3089588 0.3112898

#the class of the covariate matrix
class(X)

## [1] "matrix"

#the values in the covariate matrix
X[ ,1:6]

##          [,1]     [,2]     [,3]     [,4]    [,5]     [,6]
```

```
## [1,]  0.00000  0.00000  0.00000  0.00000  0.0000  0.00000
## [2,] 30.46081 44.20032 40.51409 25.41305 27.6765 41.67172
```

3 Model Application

Once we have prepared the data `Ometh` and `X` described before, we can use the R function `HIRE` to carry out the HIRE model in a convenient way.

```
library(HIREewas)
ret_list <- HIRE(Ometh, X, num_celltype=K, tol=10^(-5), num_iter=1000, alpha=0.01)

##  Initialization Done.

##  Implementing EM algorithm...

## Iteration: 1  observed-data log likelihood: 786977.047009
## Iteration: 2  observed-data log likelihood: 845597.548734
## Iteration: 3  observed-data log likelihood: 890628.061074
## Iteration: 4  observed-data log likelihood: 928582.504293
## Iteration: 5  observed-data log likelihood: 961110.843322
## Iteration: 6  observed-data log likelihood: 988776.683578
## Iteration: 7  observed-data log likelihood: 1011852.503606
## Iteration: 8  observed-data log likelihood: 1030600.549852
## Iteration: 9  observed-data log likelihood: 1045380.540807
## Iteration: 10  observed-data log likelihood: 1056672.531894
## Iteration: 11  observed-data log likelihood: 1065043.271409
## Iteration: 12  observed-data log likelihood: 1071085.718678
## Iteration: 13  observed-data log likelihood: 1075356.124045
## Iteration: 14  observed-data log likelihood: 1078337.083847
## Iteration: 15  observed-data log likelihood: 1080414.137330
## Iteration: 16  observed-data log likelihood: 1081876.180194
## Iteration: 17  observed-data log likelihood: 1082929.295159
## Iteration: 18  observed-data log likelihood: 1083713.865411
## Iteration: 19  observed-data log likelihood: 1084323.512061
## Iteration: 20  observed-data log likelihood: 1084818.855742
## Iteration: 21  observed-data log likelihood: 1085238.175662
```

```
## Iteration: 22  observed-data log likelihood: 1085605.540460
## Iteration: 23  observed-data log likelihood: 1085936.061253
## Iteration: 24  observed-data log likelihood: 1086239.303418
## Iteration: 25  observed-data log likelihood: 1086521.422498
## Iteration: 26  observed-data log likelihood: 1086786.482879
## Iteration: 27  observed-data log likelihood: 1087037.259105
## Iteration: 28  observed-data log likelihood: 1087275.724644
## Iteration: 29  observed-data log likelihood: 1087503.363960
## Iteration: 30  observed-data log likelihood: 1087721.317017
## Iteration: 31  observed-data log likelihood: 1087930.498253
## Iteration: 32  observed-data log likelihood: 1088131.662261
## Iteration: 33  observed-data log likelihood: 1088325.445237
## Iteration: 34  observed-data log likelihood: 1088512.391991
## Iteration: 35  observed-data log likelihood: 1088692.974167
## Iteration: 36  observed-data log likelihood: 1088867.603854
## Iteration: 37  observed-data log likelihood: 1089036.642900
## Iteration: 38  observed-data log likelihood: 1089200.410566
## Iteration: 39  observed-data log likelihood: 1089359.189746
## Iteration: 40  observed-data log likelihood: 1089513.232186
## Iteration: 41  observed-data log likelihood: 1089662.762934
## Iteration: 42  observed-data log likelihood: 1089807.984191
## Iteration: 43  observed-data log likelihood: 1089949.078641
## Iteration: 44  observed-data log likelihood: 1090086.212307
## Iteration: 45  observed-data log likelihood: 1090219.536998
## Iteration: 46  observed-data log likelihood: 1090349.192376
## Iteration: 47  observed-data log likelihood: 1090475.307698
## Iteration: 48  observed-data log likelihood: 1090598.003281
## Iteration: 49  observed-data log likelihood: 1090717.391728
## Iteration: 50  observed-data log likelihood: 1090833.578968
## Iteration: 51  observed-data log likelihood: 1090946.665130
## Iteration: 52  observed-data log likelihood: 1091056.745286
## Iteration: 53  observed-data log likelihood: 1091163.910086
## Iteration: 54  observed-data log likelihood: 1091268.246292
## Iteration: 55  observed-data log likelihood: 1091369.837229
## Iteration: 56  observed-data log likelihood: 1091468.763175
## Iteration: 57  observed-data log likelihood: 1091565.101671
## Iteration: 58  observed-data log likelihood: 1091658.927794
## Iteration: 59  observed-data log likelihood: 1091750.314375
```

```
## Iteration: 60  observed-data log likelihood: 1091839.332178
## Iteration: 61  observed-data log likelihood: 1091926.050047
## Iteration: 62  observed-data log likelihood: 1092010.535025
## Iteration: 63  observed-data log likelihood: 1092092.852448
## Iteration: 64  observed-data log likelihood: 1092173.066019
## Iteration: 65  observed-data log likelihood: 1092251.237871
## Iteration: 66  observed-data log likelihood: 1092327.428610
## Iteration: 67  observed-data log likelihood: 1092401.697354
## Iteration: 68  observed-data log likelihood: 1092474.097229
## Iteration: 69  observed-data log likelihood: 1092544.688201
## Iteration: 70  observed-data log likelihood: 1092613.525653
## Iteration: 71  observed-data log likelihood: 1092680.663110
## Iteration: 72  observed-data log likelihood: 1092746.152664
## Iteration: 73  observed-data log likelihood: 1092810.045014
## Iteration: 74  observed-data log likelihood: 1092872.389487
## Iteration: 75  observed-data log likelihood: 1092933.234059
## Iteration: 76  observed-data log likelihood: 1092992.625380
## Iteration: 77  observed-data log likelihood: 1093050.608794
## Iteration: 78  observed-data log likelihood: 1093107.228354
## Iteration: 79  observed-data log likelihood: 1093162.526843
## Iteration: 80  observed-data log likelihood: 1093216.545797
## Iteration: 81  observed-data log likelihood: 1093269.325517
## Iteration: 82  observed-data log likelihood: 1093320.905095
## Iteration: 83  observed-data log likelihood: 1093371.322433
## Iteration: 84  observed-data log likelihood: 1093420.614259
## Iteration: 85  observed-data log likelihood: 1093468.816150
## Iteration: 86  observed-data log likelihood: 1093515.962553
## Iteration: 87  observed-data log likelihood: 1093562.086806
## Iteration: 88  observed-data log likelihood: 1093607.221156
## Iteration: 89  observed-data log likelihood: 1093651.396786
## Iteration: 90  observed-data log likelihood: 1093694.643833
## Iteration: 91  observed-data log likelihood: 1093736.991415
## Iteration: 92  observed-data log likelihood: 1093778.467649
## Iteration: 93  observed-data log likelihood: 1093819.099683
## Iteration: 94  observed-data log likelihood: 1093858.913713
## Iteration: 95  observed-data log likelihood: 1093897.935015
## Iteration: 96  observed-data log likelihood: 1093936.187963
## Iteration: 97  observed-data log likelihood: 1093973.696061
```

```
## Iteration: 98  observed-data log likelihood: 1094010.481965
## Iteration: 99  observed-data log likelihood: 1094046.567509
## Iteration: 100  observed-data log likelihood: 1094081.973727
## Iteration: 101  observed-data log likelihood: 1094116.720880
## Iteration: 102  observed-data log likelihood: 1094150.828478
## Iteration: 103  observed-data log likelihood: 1094184.315299
## Iteration: 104  observed-data log likelihood: 1094217.199415
## Iteration: 105  observed-data log likelihood: 1094249.498208
## Iteration: 106  observed-data log likelihood: 1094281.228391
## Iteration: 107  observed-data log likelihood: 1094312.406027
## Iteration: 108  observed-data log likelihood: 1094343.046546
## Iteration: 109  observed-data log likelihood: 1094373.164764
## Iteration: 110  observed-data log likelihood: 1094402.774902
## Iteration: 111  observed-data log likelihood: 1094431.890604
## Iteration: 112  observed-data log likelihood: 1094460.524958
## Iteration: 113  observed-data log likelihood: 1094488.690519
## Iteration: 114  observed-data log likelihood: 1094516.399329
## Iteration: 115  observed-data log likelihood: 1094543.662944
## Iteration: 116  observed-data log likelihood: 1094570.492457
## Iteration: 117  observed-data log likelihood: 1094596.898527
## Iteration: 118  observed-data log likelihood: 1094622.891408
## Iteration: 119  observed-data log likelihood: 1094648.480970
## Iteration: 120  observed-data log likelihood: 1094673.676734
## Iteration: 121  observed-data log likelihood: 1094698.487892
## Iteration: 122  observed-data log likelihood: 1094722.923335
## Iteration: 123  observed-data log likelihood: 1094746.991677
## Iteration: 124  observed-data log likelihood: 1094770.701273
## Iteration: 125  observed-data log likelihood: 1094794.060240
## Iteration: 126  observed-data log likelihood: 1094817.075117
## Iteration: 127  observed-data log likelihood: 1094839.753146
## Iteration: 128  observed-data log likelihood: 1094862.101817
## Iteration: 129  observed-data log likelihood: 1094884.128484
## Iteration: 130  observed-data log likelihood: 1094905.840368
## Iteration: 131  observed-data log likelihood: 1094927.244549
## Iteration: 132  observed-data log likelihood: 1094948.347974
## Iteration: 133  observed-data log likelihood: 1094969.157448
## Iteration: 134  observed-data log likelihood: 1094989.679641
## Iteration: 135  observed-data log likelihood: 1095009.921083
```

```
## Iteration: 136  observed-data log likelihood: 1095029.888172
## Iteration: 137  observed-data log likelihood: 1095049.585515
## Iteration: 138  observed-data log likelihood: 1095069.002001
## Iteration: 139  observed-data log likelihood: 1095088.159459
## Iteration: 140  observed-data log likelihood: 1095107.064927
## Iteration: 141  observed-data log likelihood: 1095125.724658
## Iteration: 142  observed-data log likelihood: 1095144.144466
## Iteration: 143  observed-data log likelihood: 1095162.329879
## Iteration: 144  observed-data log likelihood: 1095180.286223
## Iteration: 145  observed-data log likelihood: 1095198.018669
## Iteration: 146  observed-data log likelihood: 1095215.532251
## Iteration: 147  observed-data log likelihood: 1095232.831881
## Iteration: 148  observed-data log likelihood: 1095249.922348
## Iteration: 149  observed-data log likelihood: 1095266.808323
## Iteration: 150  observed-data log likelihood: 1095283.494359
## Iteration: 151  observed-data log likelihood: 1095299.984889
## Iteration: 152  observed-data log likelihood: 1095316.284229
## Iteration: 153  observed-data log likelihood: 1095332.396576
## Iteration: 154  observed-data log likelihood: 1095348.326012
## Iteration: 155  observed-data log likelihood: 1095364.076505
## Iteration: 156  observed-data log likelihood: 1095379.651909
## Iteration: 157  observed-data log likelihood: 1095395.055968
## Iteration: 158  observed-data log likelihood: 1095410.292316
## Iteration: 159  observed-data log likelihood: 1095425.364480
## Iteration: 160  observed-data log likelihood: 1095440.275884
## Iteration: 161  observed-data log likelihood: 1095455.029347
## Iteration: 162  observed-data log likelihood: 1095469.627390
## Iteration: 163  observed-data log likelihood: 1095484.074210
## Iteration: 164  observed-data log likelihood: 1095498.372868
## Iteration: 165  observed-data log likelihood: 1095512.526315
## Iteration: 166  observed-data log likelihood: 1095526.537406
## Iteration: 167  observed-data log likelihood: 1095540.408905
## Iteration: 168  observed-data log likelihood: 1095554.143492
## Iteration: 169  observed-data log likelihood: 1095567.743767
## Iteration: 170  observed-data log likelihood: 1095581.212253
## Iteration: 171  observed-data log likelihood: 1095594.551396
## Iteration: 172  observed-data log likelihood: 1095607.763570
## Iteration: 173  observed-data log likelihood: 1095620.851077
```

```
## Iteration: 174  observed-data log likelihood: 1095633.816151
## Iteration: 175  observed-data log likelihood: 1095646.660961
## Iteration: 176  observed-data log likelihood: 1095659.387609
## Iteration: 177  observed-data log likelihood: 1095671.998136
## Iteration: 178  observed-data log likelihood: 1095684.494522
## Iteration: 179  observed-data log likelihood: 1095696.878691
## Iteration: 180  observed-data log likelihood: 1095709.152509
## Iteration: 181  observed-data log likelihood: 1095721.317788
## Iteration: 182  observed-data log likelihood: 1095733.376288
## Iteration: 183  observed-data log likelihood: 1095745.329718
## Iteration: 184  observed-data log likelihood: 1095757.179741
## Iteration: 185  observed-data log likelihood: 1095768.927969
## Iteration: 186  observed-data log likelihood: 1095780.575973
## Iteration: 187  observed-data log likelihood: 1095792.125278
## Iteration: 188  observed-data log likelihood: 1095803.577368
## Iteration: 189  observed-data log likelihood: 1095814.933686
## Iteration: 190  observed-data log likelihood: 1095826.195637
## Iteration: 191  observed-data log likelihood: 1095837.364358
## Iteration: 192  observed-data log likelihood: 1095848.439878
## Iteration: 193  observed-data log likelihood: 1095859.424424
## Iteration: 194  observed-data log likelihood: 1095870.319297

##  Done!

##  Calculating p-values...

##  Done!
```

Among the arguments of the `HIRE`, `num_celltype` is the number of cell types specified by the user, which can be decided by prior knowledge or the penalized BIC criterion [12]. `tol` is the relative tolerance to determine when `HIRE` stops. Specifically, when the ratio of the log observed-data likelihood difference to the log observed-data likelihood at last iteration in the absolute value is smaller than `tol`, then the HIRE functions stops. The default is 10e-5. `num_iter` is the maximum number that `HIRE` iterates with default 1000. `alpha` is a threshold parameter used in the Bonferroni correction to claim a significant cell-type-specific CpG site and to calculate the penalized BIC, and its default is 0.01.

The return value `ret_list` is an R list that consists of all parameter estimates of our interest.

```
# the class of ret_list
class(ret_list)

## [1] "list"

#the estimated cellular compositions
ret_list$P_t[ ,1:6]

##           [,1]       [,2]      [,3]       [,4]      [,5]       [,6]
## [1,] 0.1901593 0.03852461 0.2354742 0.00000000 0.2950168 0.09693634
## [2,] 0.6427212 0.57371963 0.3283484 0.94800234 0.2241097 0.32122424
## [3,] 0.1671195 0.38775577 0.4361773 0.05199766 0.4808736 0.58183942

#the estimated cell-type-specific methylation baseline profiles
head(ret_list$mu_t)

##           [,1]      [,2]      [,3]
## [1,] 0.3750383 0.2229805 0.4659452
## [2,] 0.3238664 0.2794961 0.3271067
## [3,] 0.5034966 0.6631053 0.4279233
## [4,] 0.4161573 0.3685475 0.2394163
## [5,] 0.3904720 0.4048575 0.2589418
## [6,] 0.3655453 0.4189415 0.2260232

#the estimated phenotype effects
head(ret_list$beta_t)

## [1]  0.14249392 -0.08631466 -0.10966325 -0.13608425 -0.18016851  0.01040779

#the penalized BIC value
ret_list$pBIC

## [1] -2116536

#the estimated p-values to claim whether a CpG site is at risk
#in some cell type for a covariate

#p value matrix for case/control
head(ret_list$pvalues[ ,1:3])

##           x_matr1      x_matr2      x_matr3
## [1,] 1.548811e-27 1.340803e-11 4.383043e-36
## [2,] 3.684765e-09 3.898512e-26 1.837266e-29
```

```
## [3,] 1.638936e-15 5.006148e-22 4.659029e-20
## [4,] 1.945476e-18 3.138753e-18 6.131076e-32
## [5,] 5.492153e-34 3.812436e-16 2.041123e-35
## [6,] 5.732404e-01 9.423626e-14 5.271416e-36

#p value matrix for age
head(ret_list$pvalues[ ,4:6])

##         x_matr4   x_matr5   x_matr6
## [1,] 0.9309509 0.8547646 0.4038134
## [2,] 0.7983960 0.9283010 0.4595236
## [3,] 0.9590815 0.7141966 0.8647336
## [4,] 0.1197502 0.3686029 0.7868304
## [5,] 0.6731682 0.9416187 0.9164448
## [6,] 0.8241503 0.8953657 0.1928233
```

`ret_list$P_t` is the estimated cell proportion matrix with its rows denoting cell types and columns representing samples. We can also compare the estimates with the underlying truth (see the following code). Since the deconvolution technique is unsupervised, the label-switching problem exists. Therefore, we use (2,1,3) to index `ret_list$P_t` instead of (1,2,3). `ret_list$mu_t` is the estimated cell-type-specific methylation baselines in a matrix form, where CpG sites in rows and cell types in cloumn. `ret_list$beta_t` is a three dimensional array where `ret_list$beta_t[i,k,ell]` is the estimated phenotype ell effect on CpG site i in cell type k. The penalized BIC score can be obtained by `ret_list$pBIC`.

The approximate p values are stored in the matrix `ret_list$pvalues`. Its dimension is m (the CpG site number) by Kq (the cell type number K times the phenotype number q). In the p-value matrix, one row is a CpG site. The first K columns correspond to the p-value matrix of the phenotype 1, the second K columns corresponds to the p-value matrix of the phenotype 2, and so forth.

```
#estimated cell compositions vs the truth
par(mfrow=c(1,3))
plot(ret_list$P_t[2, ], P[1, ], xlim=c(0,1), ylim=c(0,1))
abline(a=0, b=1, col="red")

plot(ret_list$P_t[1, ], P[2, ], xlim=c(0,1), ylim=c(0,1))
abline(a=0, b=1, col="red")
```

```
plot(ret_list$P_t[3, ], P[3, ], xlim=c(0,1), ylim=c(0,1))
abline(a=0, b=1, col="red")
```

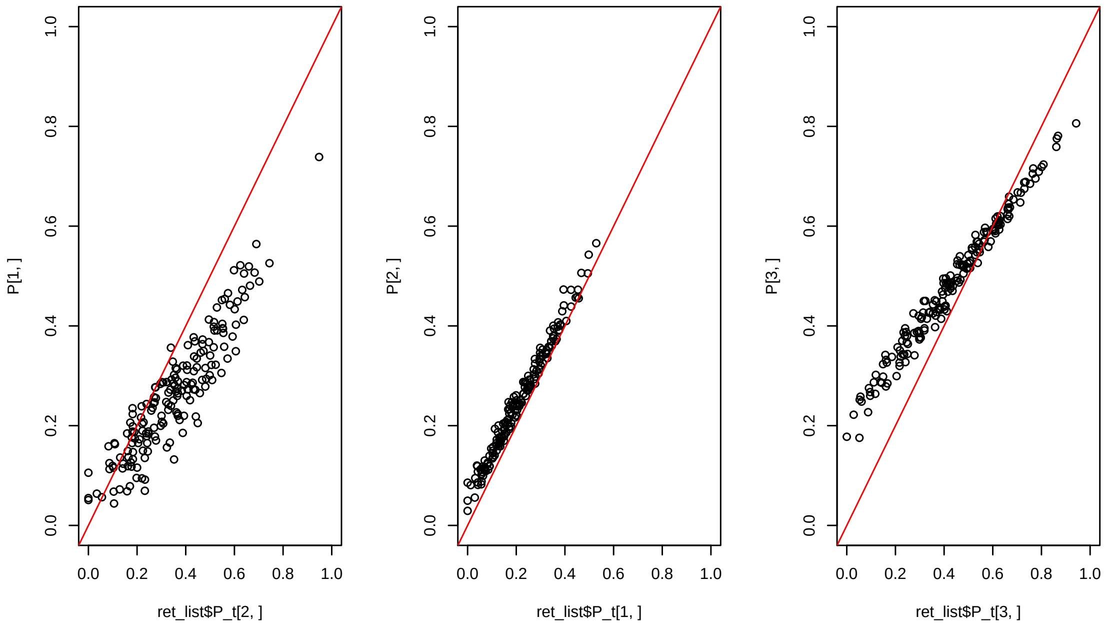

4 Visualization

We can visualize the detected association in a cell-type-specifc way using `riskCpGpattern` as follows.

```
riskCpGpattern(ret_list$pvalues[1:100, K+c(2,1,3)],
                main_title="Detected association pattern\n with age", hc_row_ind = FALSE)
```

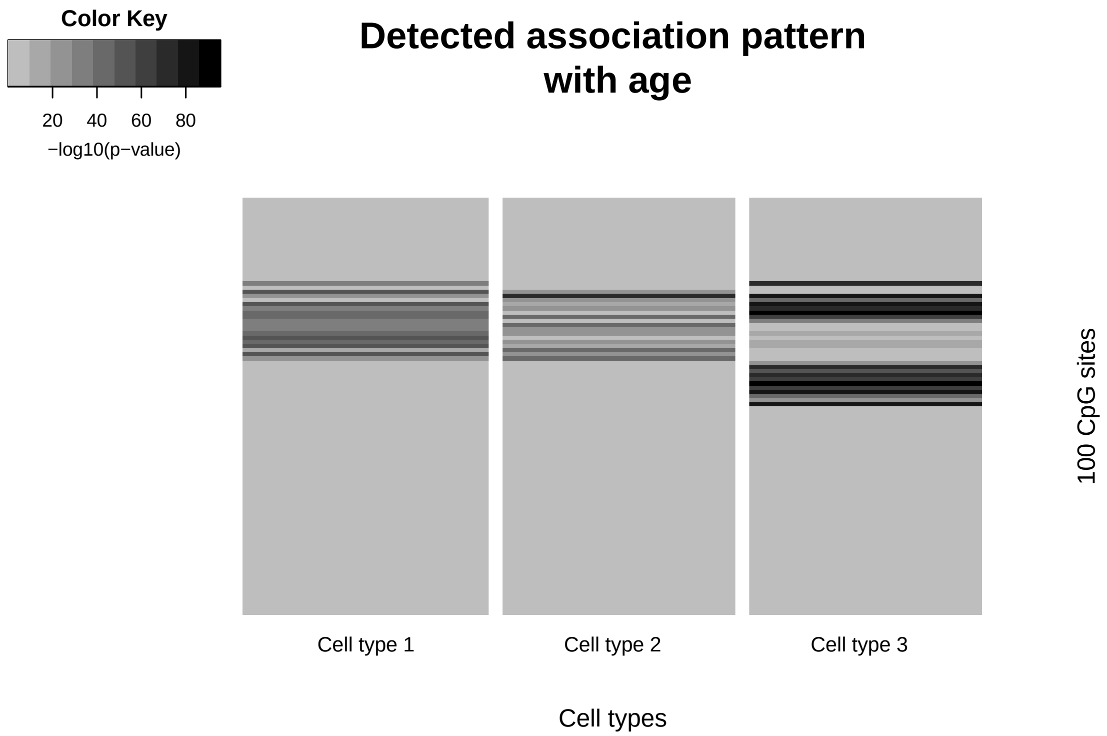

Here, for a good visualization, only the p-values for the first 100 CpG sites were demonstrated. `main_title` is used to specify the title of the association figure.`hc_row_ind` is an argument indicating whether the rows should be hierarchically clustered.

References

[1] Yun Liu, Martin J Aryee, Leonid Padyukov, M Daniele Fallin, Espen Hesselberg, Arni Runarsson, Lovisa Reinius, Nathalie Acevedo, Margaret Taub, Marcus Ronninger, et al. Epigenome-wide association data implicate DNA methylation as an intermediary of genetic risk in rheumatoid arthritis. *Nature Biotechnology*, 31(2):142–147, 2013.

[2] Eugene Andres Houseman, William P Accomando, Devin C Koestler, Brock C Christensen, Carmen J Marsit, Heather H Nelson, John K Wiencke, and Karl T Kelsey. DNA methylation arrays as surrogate measures of cell mixture distribution. *BMC Bioinformatics*, 13(1):86, 2012.

[3] Andrew E Jaffe and Rafael A Irizarry. Accounting for cellular heterogeneity is critical in epigenome-wide association studies. *Genome Biology*, 15(2):R31, 2014.

[4] James Zou, Christoph Lippert, David Heckerman, Martin Aryee, and Jennifer Listgarten. Epigenome-wide association studies without the need for cell-type composition. *Nature Methods*, 11(3):309–311, 2014.

[5] Kevin McGregor, Sasha Bernatsky, Ines Colmegna, Marie Hudson, Tomi Pastinen, Aurélie Labbe, and Celia MT Greenwood. An evaluation of methods correcting for cell-type heterogeneity in DNA methylation studies. *Genome Biology*, 17(1):84, 2016.

[6] Elior Rahmani, Noah Zaitlen, Yael Baran, Celeste Eng, Donglei Hu, Joshua Galanter, Sam Oh, Esteban G Burchard, Eleazar Eskin, James Zou, et al. Sparse PCA corrects for cell type heterogeneity in epigenome-wide association studies. *Nature Methods*, 13(5):443–445, 2016.

[7] Andrew E Teschendorff and Caroline L Relton. Statistical and integrative system-level analysis of DNA methylation data. *Nature Reviews Genetics*, 2017.

[8] William P Accomando, John K Wiencke, E Andres Houseman, Heather H Nelson, and Karl T Kelsey. Quantitative reconstruction of leukocyte subsets using DNA methylation. *Genome Biology*, 15(3):R50, 2014.

[9] Eugene Andres Houseman, Molly L Kile, David C Christiani, Tan A Ince, Karl T Kelsey, and Carmen J Marsit. Reference-free deconvolution of DNA methylation data and mediation by cell composition effects. *BMC Bioinformatics*, 17(1):259, 2016.

[10] Jeffrey T Leek and John D Storey. Capturing heterogeneity in gene expression studies by surrogate variable analysis. *PLoS Genetics*, 3(9):e161, 2007.

[11] Eugene Andres Houseman, John Molitor, and Carmen J Marsit. Reference-free cell mixture adjustments in analysis of DNA methylation data. *Bioinformatics*, 30(10):1431–1439, 2014.

[12] Wei Pan and Xiaotong Shen. Penalized model-based clustering with application to variable selection. *Journal of Machine Learning Research*, 8(May):1145–1164, 2007.
