# Supplementary material for: Detection of cell-type-specific risk-CpG sites in epigenome-wide association studies
Source: Nat Commun. 2019 Jul 15;10:3113. doi: 10.1038/s41467-019-10864-z (PMC6629651; doi:10.1038/s41467-019-10864-z)
Supplement: Supplementary file 4 — Source Data [file 41467_2019_10864_MOESM4_ESM.zip › Source Data/Rpackage HIREewas/HIREewas_Reference_Manual.pdf]

Package 'HIREewas'

May 6, 2019

Type Package

Title Detection of cell-type-specific risk-CpG sites in epigenome-wide association studies

Version 1.2.0

Date 2018-06-11

Author Xiangyu Luo <xyluo1991@gmail.com>, Can Yang <macyang@ust.hk>, Yingying Wei <yweicuhk@gmail.com>

Maintainer Xiangyu Luo <xyluo1991@gmail.com>

Description In epigenome-wide association studies, the measured signals for each sample are a mixture of methylation profiles from different cell types. The current approaches to the association detection only claim whether a cytosine-phosphate-guanine (CpG) site is associated with the phenotype or not, but they cannot determine the cell type in which the risk-CpG site is affected by the phenotype. We propose a solid statistical method, HIgh REsolution (HIRE), which not only substantially improves the power of association detection at the aggregated level as compared to the existing methods but also enables the detection of risk-CpG sites for individual cell types. The ``HIREewas'' R package is to implement HIRE model in R.

Depends R (>= 3.5.0)

Imports quadprog, gplots, grDevices, stats

VignetteBuilder knitr

Suggests BiocStyle, knitr, BiocGenerics

biocViews DNAMethylation, DifferentialMethylation, FeatureExtraction

LazyLoad yes

License GPL (>= 2)

git_url https://git.bioconductor.org/packages/HIREewas

git_branch RELEASE_3_9

git_last_commit ebc8dad

git_last_commit_date 2019-05-02

Date/Publication 2019-05-05

R topics documented:

HIREewas-package 2
HIRE 2
riskCpGpattern 6

Index **7**

HIREewas-package *Detection of cell-type-specific risk-CpG sites in epigenome-wide association studies*

Description

In epigenome-wide association studies, the measured signals for each sample are a mixture of methylation profiles from different cell types. The current approaches to the association detection only claim whether a cytosine-phosphate-guanine (CpG) site is associated with the phenotype or not, but they cannot determine the cell type in which the risk-CpG site is affected by the phenotype. We propose a solid statistical method, HIgh REsolution (HIRE), which not only substantially improves the power of association detection at the aggregated level as compared to the existing methods but also enables the detection of risk-CpG sites for individual cell types. The "HIREewas" R package is to implement HIRE model in R.

Author(s)

Xiangyu Luo <xyluo1991@gmail.com>, Can Yang <macyang@ust.hk>, Yingying Wei <yweicuhk@gmail.com>

Maintainer: Xiangyu Luo <xyluo1991@gmail.com>

References

Xiangyu Luo, Can Yang, Yingying Wei. Detection of cell-type-specific risk-CpG sites in epigenome wide association studies.

Examples

```
#Please see each function's example as well as the vignette.
```

HIRE *Detection of cell-type-specific risk-CpG sites in epigenome-wide association studies*

Description

The HIRE function provides parameter estimates in the HIRE model and the p-values for the association of CpG sites with one phenotype in each individual cell type.

Usage

```
HIRE(Ometh, X, num_celltype, tol=10^(-5), num_iter=1000, alpha=0.01)
```

Arguments

Ometh	The observed methylation matrix (with continuous values between 0 and 1), where one row corresponds to a CpG site and one column represents a sample.
X	The observed phenotypes, where one row corresponds to a phenotype and one column represents a sample.
num_celltype	The cell type number (an integer) that needs to be specified by the users.

`tol`	The relative tolerance used to determine when the HIRE stops. When the ratio of the log observed-data likelihood difference to the log observed-data likelihood at last iteration in the absolute value is smaller than `tol`, then the HIRE functions stops. The default is 10^(-5).
`num_iter`	The maximum number of iterations (an integer). The default is 1000.
`alpha`	One threshold parameter in the Bonferroni correction to claim a significant cell-type-specific CpG site (a float point between 0 and 1, usually set less than 0.05). The default is 0.01.

Details

HIRE used the generalized EM algorithm, so the log observed-data likelihood value increases after each iteration.

Value

HIRE returns a list with seven components.

`P_t`	the cellular composition matrix, where one row corresponds to a cell type and one column represents a sample.
`mu_t`	the baseline cell-type-specific methylation profiles, where one row is a CpG site and one column is a cell type.
`beta_t`	the phenotype coefficient array with dimensionality being m (the CpG number) by K (the cell type number) by q (the phenotype number). The first dimension corresponds to CpG sites, the second dimension to cell types, and the third dimension to phenotypes.
`sig_sqErr_t`	the error variance vector withlength equal to the CpG site number.
`sig_sqTiss_t`	the cell-type-specific variance matrix, where one row is a CpG site and one column represents a cell type.
`pBIC`	the penalized BIC value, where the number of phenotype coefficients being zero depends on the parameter `alpha`. If the p-value of one phenotype coefficient is greater than `alpha`/(m*K*q), we treat the phenotype coefficient to be zero.
`pvalues`	the p-value matrix, whose dimension is m (the CpG site number) by K*q (the cell type number times the phenotype number). In the p-values matrix, one row is a CpG site. The first K columns correspond to the p-value matrix of the phenotype 1, the second K columns corresponds to the p-value matrix of the phenotype 1, and so forth.

Author(s)

Xiangyu Luo

Examples

```
##########################################################################################
#Generate the EWAS data
##########################################################################################
set.seed(05222018)

#define a function to draw samples from a Dirichlet distribution
rDirichlet <- function(alpha_vec){
num <- length(alpha_vec)
```

```
temp <- rgamma(num, shape = alpha_vec, rate = 1)
return(temp / sum(temp))
}

n <- 180     #number of samples
n1 <- 60    #number of controls
n2 <- 120    #number of cases

#################################################################################################
# K=3
#################################################################################################
m <- 2000   #number of CpG sites
K <- 3       #underlying cell type number

#methylation profiles
#assume cell type 1 and cell type 2 are from the same lineage

#cell type 1
methy1 <- rbeta(m,3,6)

#cell type 2
methy2 <- methy1 + rnorm(m, sd=0.01)
ind <- sample(seq_len(m), m/5)
methy2[ind] <- rbeta(length(ind),3,6)

#cell type 3
methy3 <- rbeta(m,3,6)
mu <- cbind(methy1, methy2, methy3)

#number of covariates
p <- 2

#covariates / phenotype
X <- rbind(c(rep(0, n1),rep(1, n2)), runif(n, min=20, max=50))

#set risk-CpG sites under each cell type for each phenotype
beta <- array(0, dim=c(m,K,p))

#control vs case
m_common <- 10
max_signal <- 0.15
min_signal <- 0.07
signs <- sample(c(-1,1), m_common*K, replace=TRUE)
beta[seq_len(m_common),seq_len(K),1] <- signs * runif(m_common*K, min=min_signal, max=max_signal)

m_seperate <- 10
signs <- sample(c(-1,1), m_seperate*2, replace=TRUE)
beta[m_common+(seq_len(m_seperate)),seq_len(2),1] <- signs *
runif(m_seperate*2, min=min_signal, max=max_signal)

signs <- sample(c(-1,1), m_seperate, replace=TRUE)
beta[m_common+m_seperate+(seq_len(m_seperate)),K,1] <- signs *
runif(m_seperate, min=min_signal, max=max_signal)

#age
base <- 20
m_common <- 10
```

```
max_signal <- 0.015
min_signal <- 0.007
signs <- sample(c(-1,1), m_common*K, replace=TRUE)
beta[base+seq_len(m_common),seq_len(K),2] <- signs *
runif(m_common*K, min=min_signal, max=max_signal)

m_seperate <- 10
signs <- sample(c(-1,1), m_seperate*2, replace=TRUE)
beta[base+m_common+seq_len(m_seperate),seq_len(2),2] <- signs *
runif(m_seperate*2, min=min_signal, max=max_signal)

signs <- sample(c(-1,1), m_seperate, replace=TRUE)
beta[base+m_common+m_seperate+seq_len(m_seperate),seq_len(K),2] <- signs *
runif(m_seperate, min=min_signal, max=max_signal)

#generate the cellular compositions
P <- vapply(seq_len(n), function(i){
if(X[1,i]==0){ #if control
rDirichlet(c(4,4, 2+X[2,i]/10))
}else{
rDirichlet(c(4,4, 5+X[2,i]/10))
}
}, FUN.VALUE = rep(-1, 3))

#generate the observed methylation profiles
Ometh <- NULL
for(i in seq_len(n)){
utmp <- t(vapply(seq_len(m), function(j){
tmp1 <- colSums(X[ ,i] * t(beta[j, , ]))
rnorm(K,mean=mu[j, ]+tmp1,sd=0.01)
}, FUN.VALUE = rep(-1, K)))
tmp2 <- colSums(P[ ,i] * t(utmp))
Ometh <- cbind(Ometh, tmp2 + rnorm(m, sd = 0.01))
}

sum(Ometh > 1)
Ometh[Ometh > 1] <- 1

sum(Ometh < 0)
Ometh[Ometh < 0] <- 0


##############################################################################################
#Apply HIRE to the simulated EWAS data
##############################################################################################

#return list by HIRE
ret_list <- HIRE(Ometh, X, num_celltype=K)

#case vs control
#Visualize the association pattern with the case/control status in the first 100 CpG sites
riskCpGpattern(ret_list$pvalues[seq_len(100), c(2,1,3)],
main_title="Detected association pattern\n with disease status", hc_row_ind = FALSE)
#c(2,1,3) was used because of the label switching

#age
#Visualize the association pattern with the age in the first 100 CpG sites
```

```
riskCpGpattern(ret_list$pvalues[seq_len(100), K+c(2,1,3)],
main_title="Detected association pattern\n with age", hc_row_ind = FALSE)
```

---

riskCpGpattern *Plot the detected association pattern with one phenotype using heatmap*

Description

The detected association pattern is a way to visualize the p-values providied by the HIRE function.

Usage

```
riskCpGpattern(pval_matr, main_title = "Detected association pattern", hc_row_ind = FALSE)
```

Arguments

pval_matr	the p-value matrix for one phenotype, where one row represents a CpG site and one column indicates one cell type.
main_title	the title name. The default is "Detected association pattern".
hc_row_ind	whether we conduct hierarchical clustering in the row. The default is FALSE.

Details

This function depends on the heatmap.2 function in the gplots R package.

Value

return a heatmap

Author(s)

Xiangyu Luo

Examples

```
#a p-value matrix from the uniform distribution
pvalues <- matrix(runif(600), 100, 6)

#Visualize this p-value matrix
riskCpGpattern(pvalues,
main_title="An example", hc_row_ind = FALSE)
```

Index

∗Topic **HIRE**
HIRE, 2
riskCpGpattern, 6
∗Topic **riskCpGpattern**
HIRE, 2
riskCpGpattern, 6

HIRE, 2
HIREewas *(*HIREewas-package*)*, 2
HIREewas-package, 2

riskCpGpattern, 6
